# Active Monitoring for AtriaL FIbrillation (AMALFI): Rationale, protocol, and pilot for a pragmatic, randomized, controlled trial of remote screening for asymptomatic atrial fibrillation

**DOI:** 10.1016/j.ahj.2025.07.004

**Published:** 2025-07-16

**Authors:** Rohan Wijesurendra, Guilherme Pessoa-Amorim, Georgina Buck, Charlie Harper, Richard Bulbulia, Nicholas R. Jones, Christine A’Court, Rijo Kurien, Karen Taylor, Barbara Casadei, Louise Bowman

**Affiliations:** a Clinical Trial Service and Epidemiological Studies Unit, Nuffield Department of Population Health, University of Oxford, Oxford, UK; b Nuffield Department of Primary Care Health Sciences, University of Oxford, Oxford, UK; c National Heart and Lung Institute, Faculty of Medicine, Imperial College London, London, UK

## Abstract

**Objectives:**

Screening for asymptomatic atrial fibrillation (AF) might reduce cardioembolic strokes and screening for asymptomatic AF is recommended by some international guidelines. However, any impact of AF screening on clinical outcomes depends on a sustained increase in AF detection and anticoagulation use over time than would have occurred with routine care alone, highlighting the importance of long-term studies to generate the evidence needed to justify establishing formal screening programs.

AMALFI aims to establish the long-term efficacy and cost-effectiveness of remote screening for asymptomatic AF in older individuals at increased risk of stroke using a noninvasive 14-day continuous ECG monitoring patch in UK primary care. This paper describes the study protocol and baseline characteristics of included participants.

**Methods:**

AMALFI (ISCRTN 15544176) recruited individuals aged ≥65 years with CHA_2_DS_2_-VASc score ≥3 (men) or ≥4 (women) with no previous diagnosis of AF/atrial flutter from 27 UK primary care practices. Participants were randomized to ECG monitoring (Zio XT, iRhythm Technologies; intervention) or usual care (control). Those allocated to ECG monitoring were sent and returned the patch by mail. After wear, participants returned the patch to the device manufacturer where ECG data were analyzed via a deep-learned AI algorithm and confirmed by qualified cardiographic technicians. A final report was sent to study investigators, and those indicating AF or other arrhythmias considered by the study team to be clinically actionable were communicated to general practitioners (GPs) immediately by secure email. Additionally, GPs were notified by mail of the presence or absence of AF episodes ≥30 seconds, and of the burden of AF for each of their participants who wore a patch. The letter included signposting to relevant guidelines and findings were managed at the GP’s discretion. Participants allocated to the control group were not required to undertake any action.

The primary study outcome is the rate of new AF detection at 2.5 years, with secondary outcomes including time spent with a known AF diagnosis at 5 years of follow-up, and analyses of these outcomes by predefined age and sex subgroups. Exploratory outcomes will assess randomized assessments of time to AF detection within 2.5 and 5 years after randomization, time spent with a known AF diagnosis up to 2.5 years from randomization, and anticoagulation exposure within 2.5 and 5 years after randomization. Other exploratory long-term assessments include randomized comparisons of numbers and proportions of hospitalizations (total and cardiovascular), ischemic stroke, major bleed, and death (all-cause and cardiovascular) in both groups.

**Results:**

Between 2019 and 2022, AMALFI randomized 5,040 people in England to screening versus usual care using mail-based invitations. Participant mean age was 77 ± 6 years, with 2360 (46.8%) female; median CHA_2_DS_2_-VASc score was 4 (IQR 3-5). Follow-up data on AF diagnosis, other clinical diagnoses, prescription of oral anticoagulation and other medications, primary care encounters, referrals for secondary care, and clinical events are currently being collected from primary care practices, complemented via linkage to national-level databases including dispensing data, hospital admissions, and death records. AF detection rates will be assessed at 2.5 and 5 years after randomization, and long-term cost-effectiveness will be analyzed.

**Conclusion:**

AMALFI will provide randomized evidence on the actionable time window of opportunity for intervention generated by remote screening for asymptomatic AF in the UK using a noninvasive long-term continuous monitoring ECG patch, and the cost-effectiveness of this approach. Such data may further elucidate existing patterns of routine AF diagnosis and management, and provide important insights to guide future discussions into nationwide AF screening in the UK. AMALFI results will be reported in 2025 and 2027. (Am Heart J 2025;290:310–324.)

## Background

### Asymptomatic atrial fibrillation

Atrial fibrillation (AF) is the most common sustained cardiac arrhythmia (> 1.5 million people in England and > 59M worldwide). ^1^ The prevalence of clinically detected AF is expected to double by 2050 due to the rising prevalence of AF risk factors such as increasing age, diabetes, hypertension, and obesity. ^2,3^

Individuals with AF have a 5-fold increased risk of stroke, with embolic strokes due to AF representing approximately 25% to 33% of all ischaemic strokes. ^4,5^Moreover, embolic strokes due to AF are more commonly fatal or disabling, and associated with higher healthcare costs than nonembolic events. ^6–8^ In people with clinically-detected AF with additional cardiovascular risk factors, oral anticoagulation reduces stroke risk by about two-thirds and all-cause mortality by a quarter. ^9^ AF can be asymptomatic and occur intermittently (i.e. paroxysmal AF), and in many individuals is not clinically evident. ^10,11^ As a result, a stroke may be the initial presentation of AF and indeed in 25% of patients with an AF-associated stroke, the arrhythmia is first diagnosed at the time of admission. ^5,12–14^ Asymptomatic or undiagnosed AF has been estimated to affect 1% to 2% of the population over 65 years, with the prevalence of diagnosed AF increasing with the duration of cardiac monitoring and the presence of comorbidities. ^15^ Importantly, the presence of AF symptoms does not affect either stroke risk or the efficacy of anticoagulation in AF, ^16,17^ and anticoagulation treatment recommendations do not take symptomatic status into account. ^18,19^

### Screening for asymptomatic AF

Asymptomatic AF screening has been proposed as a way to prevent cardioembolic strokes. ^11,20^ It fulfils many of the Wilson and Jungner criteria ^21^ for a good screening program: asymptomatic AF is a common and growing public health problem; there are available, accurate, and acceptable detection approaches; and AF is an established risk factor for stroke with effective treatment options. Opportunistic AF screening (with pulse-taking or a 12-lead electrocardiogram (ECG) during a consultation for other reasons) has been recommended by some international guidelines. ^19^ However, this approach is likely to miss many short or infrequent asymptomatic AF cases, and has also been shown to be performed inconsistently in routine practice. ^22^ With the advent of new monitoring technology (e.g. single-lead ECGs, wearables, implantable loop recorders) it has been suggested that screening for AF for longer durations in relevant populations could improve health outcomes. ^11^ However, both the UK National Screening Committee (in 2019) and the United States Preventive Services Task Force (in 2022) have recommended against systematic AF screening due to a lack of high-quality evidence supporting the effectiveness of anticoagulation in screen-detected AF, and because of uncertainty regarding the best screening method. ^23^ Whilst the European Society of Cardiology Atrial Fibrillation guidelines give a class IIA recommendation that “population-based screening for AF using a prolonged noninvasive ECG-based approach should be considered in individuals aged ≥75 years, or ≥65 years with additional CHA_2_DS_2_-VA risk factors to ensure earlier detection of AF”, these guidelines also highlight the need for more research into which populations might benefit from screening, and to determine the optimal duration and burden of AF that is clinically relevant. ^24^

### Randomized assessment of AF screening

#### Screening approaches

Multiple studies (including randomized controlled trials [RCT]s) have addressed the question of how best to detect asymptomatic AF. ^25^ These have shown that screening for AF systematically and for longer durations (using a range of screening devices) will generally increase AF detection, at least in the short term. ^15^ However, in the VITAL-AF (Boston, US) and the IDEAL-MD and D2AF (Netherlands) trials, which respectively included 30,175, 17,107 and 17,976 people aged 65 years or more without a diagnosis of AF, opportunistic AF screening with a 30 second (30s) single-lead ECG did not increase new AF rates at 1 year compared with usual care. ^26–28^ These findings suggest that systematic screening using new monitoring technology (single-lead handheld-ECG) alone is not sufficient to impact clinical outcomes, and that AF screening programmes may need to include longer ECG monitoring durations in high-risk individuals in order to increase the AF detection yield.

Three US trials using a more intensive screening strategy (14-day continuous noninvasive ECG) found increased rates of newly-diagnosed AF within short follow-up periods. mSToPS randomized 2,659 people aged 75+, or 55+ males and 65+ females with an additional comorbidity, to immediate vs delayed (by 4-months) screening with a 14-day ECG patch (the iRhythm Zio XT) worn twice within a period of 3 months, and found a 4-fold increase in newly-diagnosed AF at 4 months (3.9% in the active group vs 0.9% in the control group). ^29^ SCREEN-AF randomized 856 people aged 75+ with hypertension to screening with a 14-day iRhythm Zio XT, worn twice within 3 months, versus usual care, and found a 10-fold increase in new diagnoses of AF at 6 months (5.3% in the active group vs 0.5% in the control group). ^30^ GUARD-AF randomized US participants aged 70 years or older to continuous, noninvasive, single-lead 14-day monitoring with an ECG patch vs usual care. ^31^ After a median of 15.3 months, there were 260 patients (5%) with a new diagnosis of AF in the screening group compared to 175 (3.3%) in the control group. Taken together, these trials confirm that more intensive screening can increase AF detection in the short term, but, crucially, they also suggest that the gain of AF detection obtained by screening may decrease rapidly with the duration of follow-up. In other words, short-term studies are likely to over-estimate the lifetime difference between screening and usual care by not capturing the rate of AF detection via usual care over time. This is exemplified by the REHEARSE-AF trial, which randomized 1001 UK patients aged 65 years or more with CHA_2_DS_2_VASc≥ 2 to intermittent twice-weekly 30s handheld-ECG monitoring over a year versus routine care, and found a 4-fold increase in the rate of new AF detection after 1 year (3.8% in the active group vs 1.0% in the control group). ^32^ A follow-up extension study showed that this difference had diluted to become nonstatistically significant after a median of 4.2 years (43 new diagnoses in the active group vs 31 in the control group). ^33^ Any impact of AF screening on clinical outcomes depends on a sustained increase in AF detection and anticoagulation use over time than would have occurred with routine care alone, highlighting the importance of long-term studies in generating the evidence needed to justify establishing formal screening programs.

#### Anticoagulation in device-detected AF

Two trials have addressed whether anticoagulation reduces the risk of clinical embolic events in patients with subclinical AF detected using implanted pacemakers or defibrillators (i.e., device-detected atrial high-rate episodes lasting at least 6 minutes). ^34,35^ NOAH-AFNET randomized 2536 patients aged 65 years or older with an additional risk factor for stroke, to edoxaban vs placebo. The trial was terminated early after a median follow-up of 21 months due to safety concerns, driven by a 2-fold increase in the risk of major bleeding in the active arm, and because of a tendency towards futility (after 184/220 planned primary efficacy events had accrued). The final results showed a nonstatistically significant 19% relative risk reduction (RRR) (hazard ratio [HR]: 0.81; 95% confidence interval [CI]: 0.60-1.08; *P* = .15) in the primary composite efficacy endpoint of stroke, systemic embolism, or cardiovascular death (and a nonsignificant 21% RRR in ischaemic stroke; HR 0.79 [0.45-1.39]). The reduction in systemic embolism in the active arm (HR 0.51; 95% CI 0.27-0.96) was largely driven by a lower rate of myocardial infarction (10/2589 [0.4%] versus 16/2524 [0.6%]) and pulmonary embolism (3/2589 [0.1%] versus 9/2533 [0.4%]). Conversely, there was a 31% relative increase (HR, 1.31 [1.02-1.67]; *P* = .03) in the primary composite safety endpoint of major bleeding or all-cause death, and a doubling of major bleeding rates (HR, 2.10 [1.30-3.38], *P* = .002).

ARTESIA randomized 4102 patients with CHA_2_DS_2_VASc ≥3 to apixaban vs aspirin. After a median of 3.5 years, there was a 37% RRR in stroke or systemic embolism (HR: 0.63 [0.45-0.88]; *P* = .007), and a 36% relative increase in major bleeding (HR: 1.36 [1.01-1.82], *P* = .04). Of note, aspirin does not provide any stroke reduction benefit in AF (as acknowledged by the ARTESIA authors), and use of a placebo-comparator would likely have resulted in larger differences in the rate of major bleeding between the groups (as seen in NOAH-AFNET).

Taken together, these trials suggest that the net clinical benefit of oral anticoagulation in device-detected AF is borderline, because the ~35% relative reduction in stroke or systemic embolism is offset by a 30-35% relative increase in major bleeding. Of note, the stroke event rates in these patients with short episodes of device-detected asymptomatic AF (0.9% to 1.2% per person/year) were much lower than in equivalent patients with clinically-detected AF. ^36,37^ This illustrates that ever more intensive screening, as advocated by some ^38^ will almost inevitably result in detection of individuals with lower AF burden. Although AF burden is not considered a risk factor for stroke in clinical guidelines, ^18,19^ the results of NOAH-AFNET and ARTESIA, as well as other studies, ^39^ suggest that a lower AF burden is associated with a lower risk of embolic events, and that the net clinical benefit of anticoagulation in these individuals is therefore much less clear compared to that in individuals with clinically-detected AF (who tend to have a higher AF burden).

#### Ongoing uncertainty and need for robust evidence

The selection of the screening population, duration of ECG monitoring, and the implementation and accessibility of the program are crucial elements in the design of AF screening initiatives. For AF screening to improve clinical outcomes it must not only increase AF detection, but any AF detected must also carry a similar risk of stroke or systemic embolism as that associated with AF detected clinically, and finally it must also increase anti-coagulation uptake long-term. Moreover, any incremental benefits from systematic AF screening also depend on the frequency with which individuals interact with health services (e.g., secondary to age/comorbidity) and uptake of opportunistic screening, which will vary between healthcare systems. In summary, the current evidence is insufficient to support systematic AF screening, ^23,40^and further trials are needed to assess its efficacy and cost-effectiveness. ^25^

#### Aim and anticipated value of the AMALFI trial

The Active Monitoring for AtriaL Fibrillation (AMALFI) trial is a streamlined randomized controlled trial of screening for AF in the UK using 14-day patch-based continuous ambulatory ECG monitoring delivered and returned by mail. This approach, involving no physical sites or study visits, was taken with a view to stream-lining delivery, reaching out to individuals who may be less likely to access care, and minimizing costs in any future national screening programme, whilst simultaneously making the study accessible to participants. AMALFI was designed to target recruitment of older individuals at moderate-to-high stroke risk, as this population is expected to have a higher prevalence of asymptomatic AF ^34,35^ and a thrombotic risk that would warrant consideration of anticoagulation if AF were detected.

AMALFI will provide randomized evidence on the actionable time window of opportunity for intervention generated by remote screening for asymptomatic AF in the UK using a noninvasive long-term continuous monitoring ECG patch, and the cost-effectiveness of this approach. Such data may further elucidate existing patterns of routine AF diagnosis and management and provide important insights to guide future discussions into nationwide AF screening in the UK. Here, we present the rationale, protocol, and baseline characteristics for the trial.

## Methods

### Objectives

**Figure 1:**
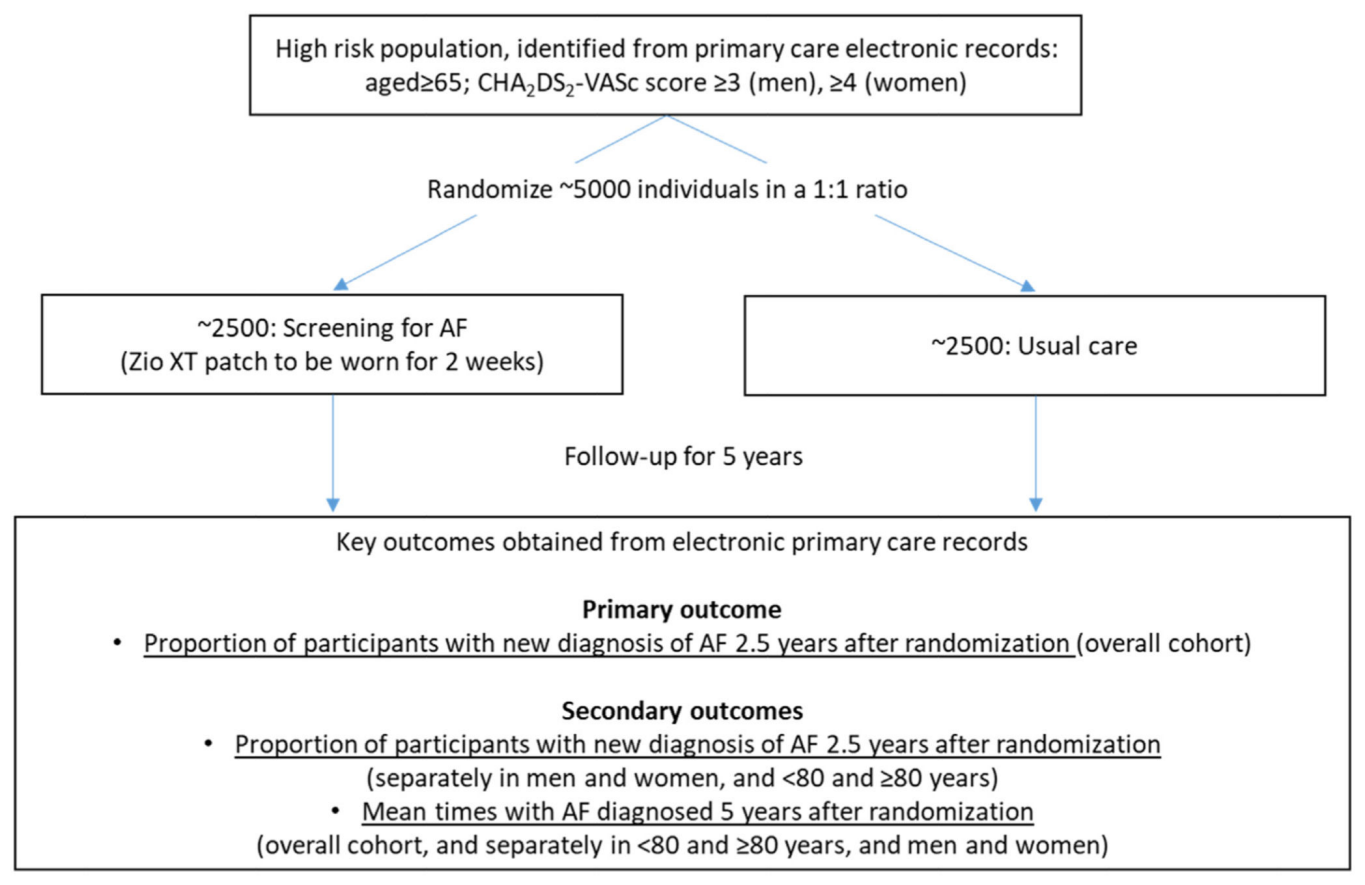
Study outline. AF, atrial fibrillation.

AMALFI aims to establish the long-term efficacy and cost-effectiveness of remote screening for asymptomatic AF in older individuals at increased risk of stroke using a noninvasive 14-day continuous ECG monitoring patch in UK primary care (Figure 1). The main study outcomes are new AF detection rates at 2.5 years and time spent with a known AF diagnosis at 5 years of follow-up. These outcomes will also be assessed in predefined age and sex subgroups, and exploratory outcomes will assess randomized assessments of time to AF detection within 2.5 and 5 years after randomization, time spent with a known AF diagnosis up to 2.5 years from randomization, and anticoagulation exposure within 2.5 and 5 years after randomization. Other exploratory long-term assessments include randomized comparisons of numbers and proportions of hospitalizations (total and cardiovascular), ischemic stroke, major bleed, and death (all-cause and cardiovascular) in both groups. Key features of the study design are described below, and the full study protocol is available in the Supplementary Material (Appendix S2).

### Study population

AMALFI recruited men or women aged 65 years or more with a CHA_2_DS_2_VASc score ≥3 in men or ≥4 in women. ^19^ Targeted recruitment of an older population with additional cardiovascular risk factors allowed selection of individuals with an increased likelihood of having asymptomatic AF, ^41^ whilst also having a thrombotic risk warranting consideration of anticoagulation if AF were found. The key exclusion criterion was a previous diagnosis of AF or atrial flutter.

### Study setup and funding

AMALFI is an investigator-initiated study coordinated by the Clinical Trial Service Unit (CTSU) at Oxford Population Health, University of Oxford (the trial sponsor). Funding is provided by the National Institute for Health and Care Research (NIHR) Oxford Biomedical Research Centre and the British Heart Foundation (CS/F/23/190062). iRhythm supported the study by providing Zio XT ambulatory monitoring patches, ECG analysis and cardiac technician data review at no charge. iRhythm had no role in study design, conduct, or reporting. Additional logistical support (namely identification of suitable primary care practices) was provided to the study by NIHR Clinical Research Networks (CRN) in Thames Valley and South Midlands, East Midlands, West Midlands, and West of England.

### Recruitment and intervention

#### Screening and invitation

The study population was identified via automated electronic health record searches performed in participating primary care practices, using a bespoke approach to identify qualifying risk factors. The resulting list was reviewed by general practitioners (GPs) in each practice for approval before invitation. Contact details were uploaded to a standard mailing service, merged with the study documentation, and mailed to potential participants. Eligibility was based on the electronic search, and no manual verification was required by either GPs or the study team. Those individuals willing to take part registered their interest by completing and returning a randomization questionnaire and consent form to the coordinating centre. The randomization questionnaire asked about the individual components of the CHA_2_DS_2_VASc score. Further information for participants and GPs was available on the study website, ^42^ along with support by email and a 24-hour Freephone telephone service.

#### Randomization

Participants were randomized 1:1 between intervention (patch) and control (usual care) groups by a central computer program. A minimization algorithm was used to ensure adequate balance between both groups with regards to important features associated with the presence of AF and stroke, namely age, gender, and residual CHA_2_DS_2_VASc score. ^43^

#### Intervention and control groups

AMALFI is testing a remote screening strategy using an external noninvasive ECG patch (Zio XT, iRhythm Technologies, San Francisco, CA, USA), which has been Food and Drug Administration (FDA)-cleared and UK Conformity Assessed (UKCA) approved. The Zio XT patch (here-after referred to as “patch” or “ECG monitor”) is a long-term continuous ambulatory ECG monitor applied to the chest that stores up to 14 days of continuous ECG data.

AMALFI used a 14-day monitoring strategy because studies have shown high diagnostic yield and lower retesting rates of 14-day continuous uninterrupted monitoring with Zio XT compared to shorter duration and intermittent monitoring strategies and devices. ^44–47^

Participants allocated to the intervention group were sent the patch by mail and asked to self-apply and wear it continuously for 14 days. Instructions were included with the patch and a short video was available on the study website, as well as further assistance via telephone or email. After the end of the 14-day period (or before, if the patch fell off or was removed), participants were asked to post the patch back to the manufacturer using a prepaid box. After a few weeks, a reminder letter was sent to those who had not yet returned their patch. Those allocated to the control group received a letter confirming their study allocation and were not required to undertake any further action. GPs were also informed of each participant’s inclusion in the trial and group allocation.

#### ECG monitoring data analysis

The recorded ECG data were analyzed by iRhythm using a validated proprietary deep-learning algorithm, which has been FDA-cleared and UKCA-approved, and is capable of detecting 13 arrhythmias (including AF), plus sinus rhythm and noise. The algorithm has previously shown high diagnostic performance when compared with a committee of certified cardiologists. ^48^ Briefly, the algorithm was trained on 91,232 ECG records and when tested on an independent dataset annotated by board-certified cardiologists, the deep neural network achieved an average receiver operating characteristic (ROC) of 0.97 and an F1 score (harmonic mean of positive predictive value and sensitivity) of 0.837, surpassing the average cardiologists’ score of 0.780. AF is defined as the presence of at least 30 seconds of continuous AF-compatible tracing during monitoring. Candidate findings were reviewed and classified by trained cardiovascular technicians at iRhythm. ^44^ A report describing rhythm findings was then generated and provided to the study team in Oxford using a secure web-based system. No manual classification or over-read was performed by the study team, and no further tests were required for confirmation of AF diagnoses (although GPs could perform additional testing if desired). Automated alert systems were in place for AF and other arrhythmias considered by the study team to be clinically actionable (i.e., atrial flutter; sustained ventricular tachycardia or fibrillation; pauses lasting over 6 seconds; complete heart block, Mobitz type II, or high-grade atrioventricular block), which were communicated to GPs immediately. Separately, the study team received regular structured data extracts from iRhythm including all ECG findings. A results letter was generated automatically from these data extracts and sent to GPs, with a copy to participants. This letter detailed the presence or absence of AF, along with duration, heart rate, and burden. Determination of AF burden using Zio XT has been previously validated against an atrial pacemaker lead demonstrating very high correlation (R-square = 0.9999). ^49^ Following the pilot study (see Results section), complete monitoring reports were not routinely provided to GPs unless there was a new diagnosis of AF or a clinically actionable finding (or at the GP’s or participant’s request).

#### Clinical management

Monitoring results were provided to the participants’ GP, who managed any findings at their discretion according to UK usual care. No clinical guidance on the management of either AF or incidental findings was provided by the study team, besides signposting to relevant guidelines. ^19,50^

### Data collection

AMALFI is an entirely remote study, with no physical study sites and no study visits. All communication with participants and their GPs is performed by mail, telephone, or email. Clinical data for baseline assessments and study outcomes are extracted using automated, batch data exports from primary care records in each participating GP practice. These extracts are focused on basic demographics, clinical diagnoses, prescribed medications, primary care encounters, and referrals for secondary care. These data are complemented via linkage to national-level databases held by National Health Service England (NHSE; this includes nationwide dispensing data, hospital admissions, and death records). Self-reported quality-of-life data are also being collected using standard EQ-5D-5L questionnaires. Details of the definition and derivation of baseline characteristics and outcomes are described in Supplementary Material (Appendix S3).

### Study outcomes

#### Primary outcome

**Figure 2:**
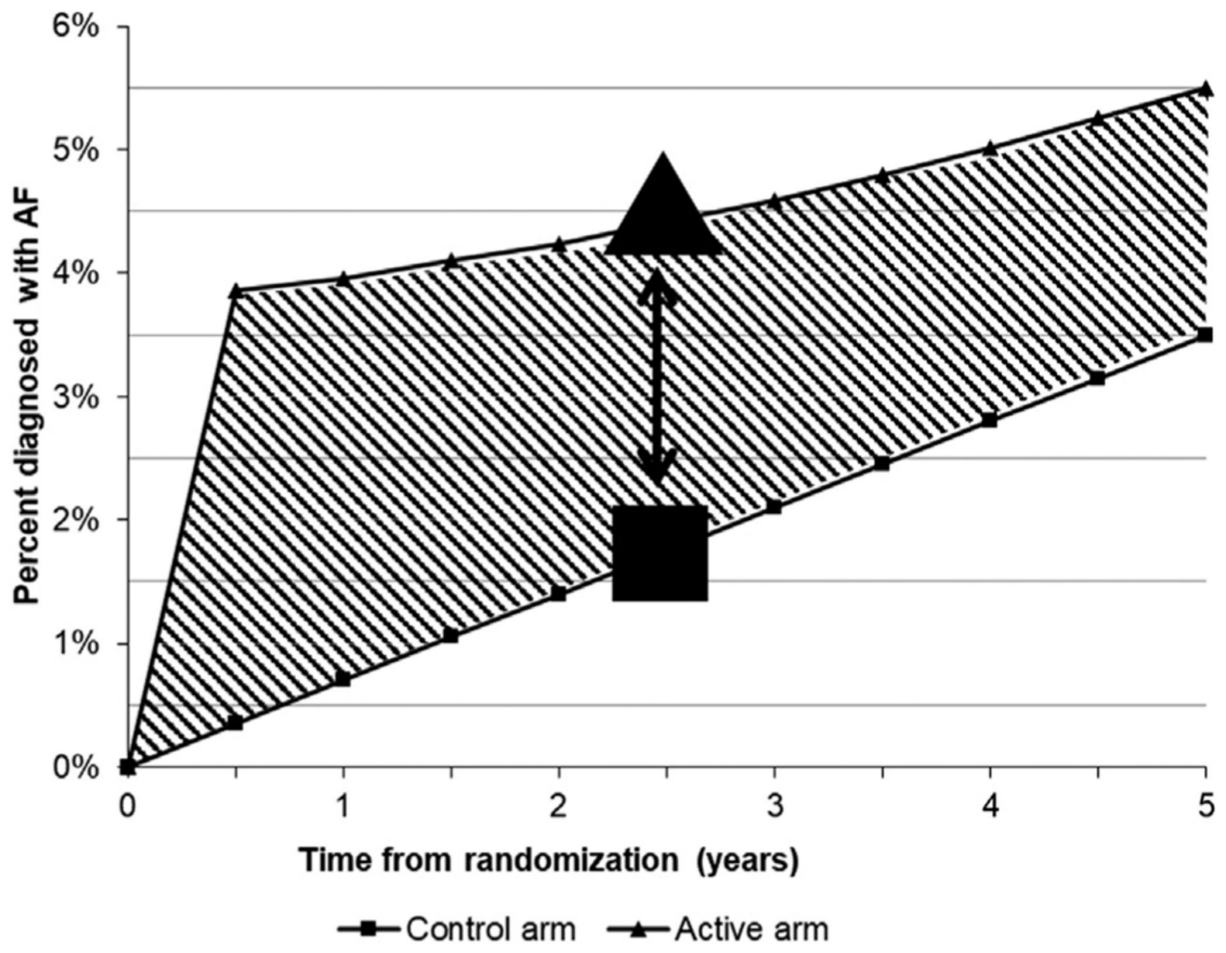
Estimated cumulative percent of participants diagnosed with AF. Each curve represents the cumulative proportion of new AF diagnoses in the active arm (small triangles) and control arm (small squares). The primary outcome is the proportion of people with AF recorded within primary care records within 2.5 years after randomization in each arm (large solid triangle and square). The difference between both is represented by the double-headed arrow. The secondary outcome is the mean time spent with an AF diagnosis over a period of 5 years after randomization (from the date of first postrandomization AF record until right-censoring at 5 years after randomization, or death/withdrawal if earlier); this is represented by the area-under-the-curve for each curve (active arm and control arm). The difference between both is the dashed area between the curves.

The main aim of AMALFI is to assess the long-term efficacy of AF screening. Conceptually, this may be interpreted as the actionable time window of opportunity for intervention resulting from screening. This window is represented by the difference in areas under the cumulative incidence curves – representing total time spent with an AF diagnosis - between the active and control groups, with those not having AF contributing zero time (Figure 2). The timeframe for such an assessment of long-term efficacy should represent a proposed time interval between screening events if translated into clinical practice—e.g., 5 years. Hence, the ideal primary outcome for this study would be the times with AF diagnosed at 5 years in each group.

However, an earlier assessment of the expected value of the proposed screening approach can be performed by assessing the proportion with AF detected in each arm within a period of 2.5 years from randomization—which, based on the statistical modelling performed of the expected AF rates during follow-up, is the largest driver of the expected mean time spent with a known diagnosis of AF within a period of 5 years from randomization. Therefore, the primary outcome assessment will involve an intention-to-treat comparison among all randomized participants allocated to screening versus usual care on the presence of a record of AF (as recorded in primary care data) occurring between randomization and 2.5 years after randomization (i.e., at around the mid-point of a proposed 5-year screening interval). Outcome ascertainment is described in detail in the Supplementary Material (Appendix S3).

#### Secondary outcomes

The secondary outcome assessments for the study will involve intention-to-treat comparisons among all randomized participants allocated to screening versus usual care on:

- The presence of a postrandomization record of AF within a period of 2.5 years from randomization (as in The primary outcome assessment) by subgroups of age (< 80 and ≥ 80 years) and, separately, sex; and
- Time spent with a known AF diagnosis within a period of 5 years from randomization (as described in the previous section), in the overall cohort and in the same subgroups mentioned above.

Both proposed subgroup analyses are of clinical interest—the yield of screen-detected AF (and stroke incidence) increase greatly with age, ^51^ but older age is also associated with higher bleeding risks from anticoagulation, competing causes of mortality other than stroke, and increased healthcare contact for other reasons (which increases the likelihood of AF detection via usual care). Similarly, AF is more frequent in men, with women usually developing clinically-evident AF 10 years later; ^52,53^ thus any potential benefits (and cost-effectiveness) of screening might also differ according to sex. ^54^

Additional detail on the assumptions underlying the expected mean times with AF are provided in Supplementary Material (Section S1). This outcome will be reported separately in due course.

#### Exploratory outcomes

Exploratory outcomes will assess randomized assessments of time to AF detection within 2.5 and 5 years after randomization, time spent with a known AF diagnosis up to 2.5 years from randomization, and anticoagulation exposure within 2.5 and 5 years after randomization (total number of participants exposed, time to initiation, and total exposure). Additionally, long-term assessments will also include randomized comparisons of numbers and proportions of hospitalizations (total and cardiovascular), ischemic stroke, major bleed, and death (all-cause and cardiovascular) in both groups. Finally, to explore how the mode of AF diagnosis may impact future management decisions, AF recording patterns and rates of anticoagulation will also be compared (in a nonrandomized fashion) between participants with AF detected via patch (screen-detected) versus clinical care (in both screening and control groups). Such data may further elucidate existing patterns of routine AF management and provide important insights to guide future discussions into nationwide AF screening in the UK.

### Planned statistical analyses

The main analyses will be performed following the intention-to-treat principle and including all randomized participants. The chi-square test will be used to assess the primary outcome of AF rates at 2.5 years of follow-up in the entire cohort, the secondary/exploratory assessments of the same outcome by sex and age subgroups (with a test for heterogeneity between subgroups), and the exploratory outcome of proportion of participants with postrandomization anticoagulation records. Outcomes of mean durations of time with AF diagnosed (after 2.5 and 5 years of follow-up) will be compared using a permutation test to assess statistical significance, in the overall cohort and by subgroups of sex and age (with a test for heterogeneity). ^55,56^ Those with no diagnosis of AF by the end of the study will contribute zero time with AF. The outcome of total exposure to anticoagulation postrandomization will also be assessed using a permutation test. Assessment of time to AF diagnosis and anticoagulation initiation will be performed using the log-rank test. A two-tailed *P*-value of < .05 will be used to determine statistical significance in all proposed analyses. The full statistical analysis plan is contained in the Supplementary Material (Appendix S4).

### Health economics

AMALFI will also inform a cost-effectiveness analysis aimed at capturing differences in healthcare resources (e.g., GP visits, medications, hospital visits), as well as differences in health-related quality-of-life (based on the EQ-5D questionnaire), and AF-related events. Details of these analyses will be described separately.

### Sample size calculations

The sample size for this study was initially specified as 2,500 but was later increased to 5,000. The underlying assumptions are as follows:

#### AF detection via usual care

A 2015 UK study on opportunistic AF screening during flu vaccinations (which are routinely offered to those aged 65 years of more in addition to other groups such as those with certain long-term health conditions) suggests that the yearly rate of asymptomatic AF detection through usual care is ~0.7% among those who receive a pulse check (approximately half those who had a flu vaccination). ^22^ Thus, if considering the entire population aged 65 years or more (of whom only some will have pulse checks), the rate of newly-detected AF might be expected to be half, at ~0.3% to 0.4%/year. As AMALFI participants will generally have a higher cardiovascular risk than the general population, the yearly AF detection rate in the usual care group in AMALFI was estimated to be ~0.7%/year. Over a 2.5-year period, the new AF detection rate in the usual care group is expected to be 1.75%, based on a time dependent Poisson process (*a* +bt) with parameter b slightly increasing with time.

#### AF detection via screening

In STROKESTOP, screening with twice-daily, 30 second ECG over 14 days in 75 year-olds detected new AF in 3.0%. ^57^ It is expected that continuous monitoring with the Zio XT during a similar period of time in AMALFI will result in higher AF detection rates. However, patch screening will not detect all AF cases in the active arm, due to noncompliance with the patch, false negatives, or AF episodes not occurring at the time of screening; thus, additional AF cases will continue to be detected through usual care in this arm. Taking into consideration a moderate level of nonadherence and false-negatives (i.e., AF missed during the monitoring period by chance), it was estimated that screening would lead to newly-detected AF in about 3.75% of individuals, with additional new AF cases found via usual care throughout the duration of study. Over a 2.5-year period, it is estimated that the total new AF detection rate in the active arm will be 4.4%. This estimate takes the same Poisson process used in the usual care arm (with expected annual AF incidence 0.7%) and assumes that 70% of individuals with AF that would have been detected in year 1 without screening were found by the patch, with corresponding figures of 60% for year 2, and 50% for year 3.

#### Sample size calculations

Based on these assumptions, an initial sample size of 2,500 individuals (1,250 in each arm) was chosen to provide > 90% power to detect the expected difference in proportions of newly-detected AF between arms at 2.5 years (1.75% versus 4.4%; ratio: approximately 2.5) at two-sided p (2p) < 0.05.

### Modifications to the sample size

As recruitment progressed, it became apparent that the trial would be able to reach its prespecified target without exhausting the available pool of interested GP practices, or the allocated funding. Based on an interim analysis of baseline characteristics and AF detection rates via the patch only in 1,250 randomized participants (but blinded to total AF detection rates in both arms), the study investigators decided to extend the target study size to 5,000 in June 2021. This extension allowed:

1. Increased statistical precision to detect the estimated 2.5-fold difference in AF proportions between the two groups at 2.5 years (> 90% power at 2*P* < .01);
2. Preserved statistical power if the observed difference between groups was smaller than expected (i.e., > 90% power at 2*P* < .01 for a 2-fold difference)—per example, due to higher new AF detection rates through usual care in the control arm;
3. Sufficient power to analyse the primary outcome for subgroups of sex and, separately, age at randomization (cut-off 80 years), with ~90% power at 2*P <* .01 in both assessments; and
4. Increased power to detect differences in self-reported quality-of-life (for health economics analyses), as well as other exploratory outcomes such as anticoagulation exposure, hospitalisations (all-cause and cardiovascular), ischaemic stroke, major bleeding, and death (all-cause and cardiovascular).

Further details are provided in the Supplementary materials (section S2 and Supplementary Tables 2-5).

## Results

### Ethics and regulatory approvals

The study protocol and subsequent modifications were approved by the University of Oxford (trial sponsor), the London Bromley Research Ethics Committee (reference 19/LO/0220), and the Health Research Authority (IRAS 234837). The trial is registered in the ISCRTN registry (15544176) and the NIHR portfolio (CPMS 41053).

### Pilot stage

A small pilot took place in May/June 2019 in 2 practices, with the main aim of testing the remote recruitment approach, response rates, and patch adherence. In total, 1,208 invites were sent, leading to 284 randomizations (24% response rate). Monitoring reports were received for 118 of 143 participants in the patch arm (82%), and AF was found in 5 individuals (3.5% of the active group). Taken together, these results supported the original study design. However, there was an unexpectedly high prevalence of incidental findings: 110 (93%) with supraventricular tachycardia and 47 (41%) with nonsustained ventricular tachycardia (among other findings). As the complete monitoring reports were being provided to GPs during the pilot stage (see Supplementary Appendix S1 for a sample), this generated significant additional workload to investigate all these incidental findings (only 4 [3%] of reports showed no AF or other incidental findings), including specialist cardiology referrals. The majority of these were of uncertain clinical significance in an older population with established cardiovascular risk factors, especially as any need for further investigation would generally depend on symptoms. It was therefore decided that, in order to preserve the feasibility of the study (and of a potential large-scale screening program), complete monitoring reports would not be provided to GPs, unless at their request or in the case of a clinically significant finding. Instead, a results letter focused only on any AF findings was provided following the pilot stage (see Methods section), in line with the approach in a similar study. ^58^ All participants were advised that they should seek medical care as usual if they felt unwell for some reason, regardless of their monitoring results.

### Recruitment

**Figure 3:**
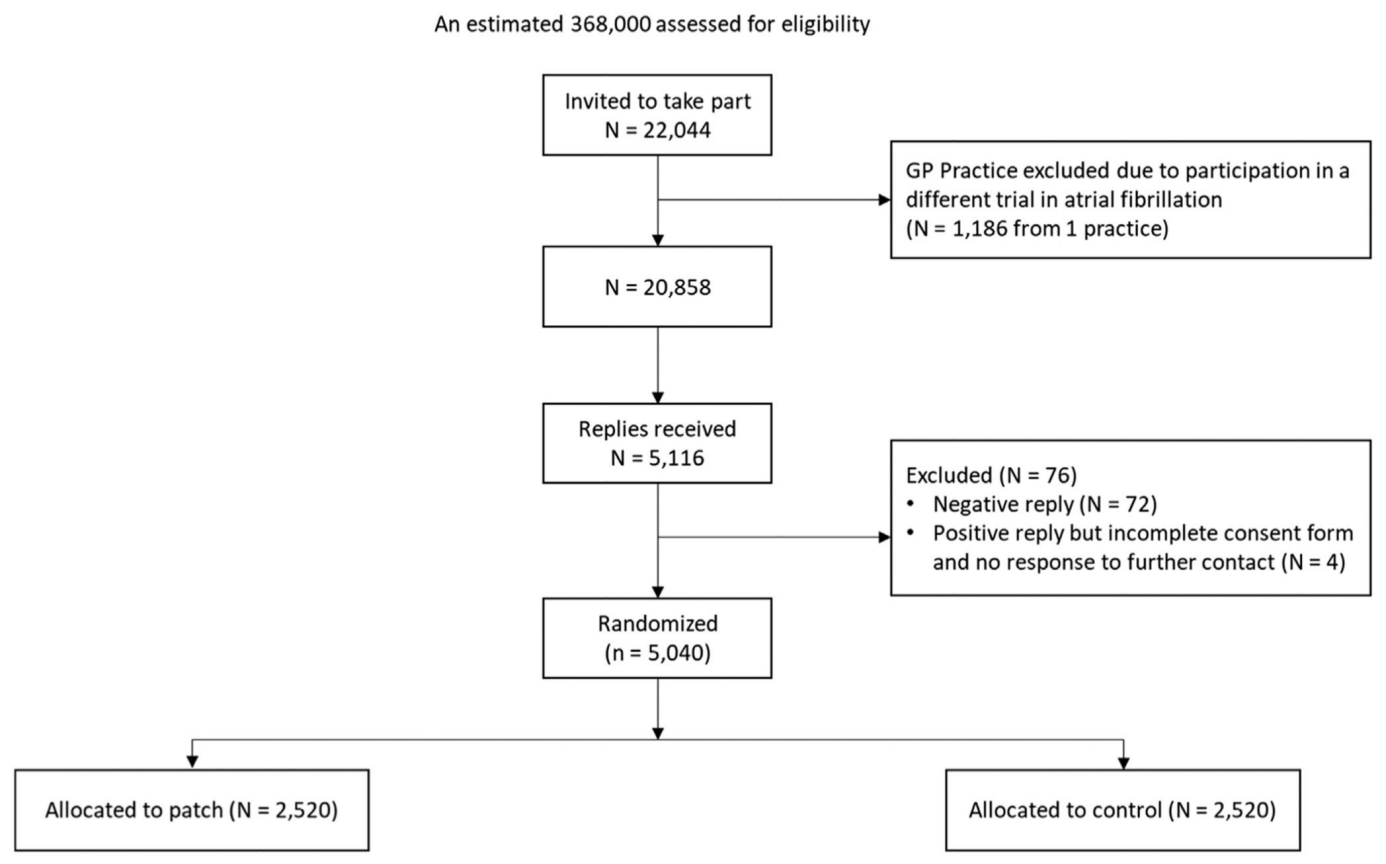
CONSORT diagram.

A CONSORT flowchart is provided in Figure 3. Twenty-eight GP practices identified participants for the trial, however 1 practice was later excluded due to concurrent participation in another AF screening trial. Due to the study design, the total number of individuals assessed for eligibility is unknown, but it includes all individuals registered at these practices regardless of age or medical history. However, based on a subset of participants (3,214) randomized in GP practices for whom the total list size is known (total = 234,633), it is estimated that approximately 368,000 individuals were electronically assessed for eligibility.

A total of 22,044 invites (6% of total estimated screened) were mailed to individuals considered eligible, (20,858 after exclusion of the ineligible practice) and replies were received from 5,116 individuals. A minority could not be included (Figure 3), with 5,040 eventually logged and randomized from 27 GP practices (enrolled-to-invited ratio 0.23; Supplementary Table 1). It was later detected that 3 individuals were randomized twice. This occurred due to two participants requesting a new invitation letter as they had lost the original one (but subsequently found it and returned both), and 1 participant moving house and registering at a new practice that was also taking part in the study (and therefore receiving a second invitation). In all 3 cases, the second randomization record was excluded; no participant whose first randomization was to the control group returned a patch, and no participant returned more than 1 patch.

### Baseline characteristics of randomized participants

**Figure 4:**
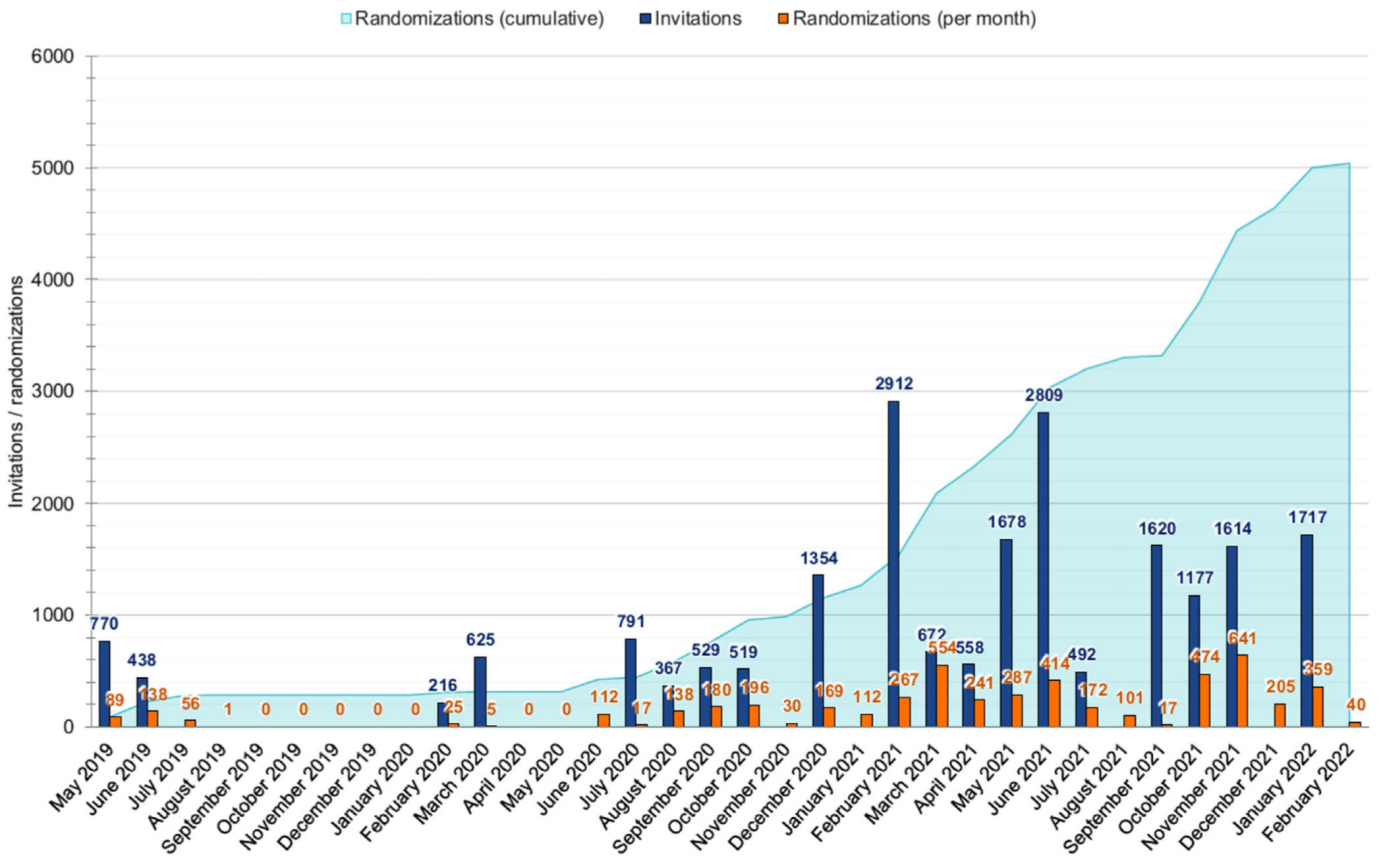
AMALFI recruitment.

AMALFI randomized 5,040 participants between May 2019 and February 2022 (22.9% of those invited). Recruitment was paused during the second half of 2019 (after the pilot stage) in order to gain ethics approval for the amended study processes required, and in early 2020 due to the COVID-19 pandemic (Figure 4). The initial recruitment target (2,500) was achieved in May 2021.

At randomization, AMALFI participants had a mean age of 78 years (standard deviation: 6 years), and 47% were female (Table 1). The majority (90%) had a prior history of hypertension and 19% had a prior stroke or TIA. A small proportion (7%) were taking oral anticoagulation prior to randomization, most likely for treatment or prevention of pulmonary embolism or deep vein thrombosis, as 12% had a history of a prior thromboembolism. Further characteristics of the study population are provided in Table 1. The minimized randomization achieved good balance of baseline prognostic factors for AF and other cardiovascular diseases.

### Intervention and postrandomization follow-up

Details on patch monitoring adherence as well as new AF detection rates by patch and incidental findings will be reported with the main study results. Follow-up for the primary outcome (2.5 years follow-up) was completed in August 2024, and is scheduled to continue until February 2027 for the secondary outcome (5 years follow-up). Data collection is ongoing from primary care records at the participating practices and through linkage to NHSE datasets.

## Discussion

Opportunistic, single-time point AF screening is recommended by several scientific societies, and is currently part of established clinical practice. ^24^ Novel ECG monitoring technology provides opportunities for expanded AF screening, but there is still significant uncertainty as to its benefits and harms. Importantly, the landmark SAFE trial, upon which current screening recommendations are made, assessed new AF diagnoses rather than stroke rates. ^59^ Moreover, several recent large randomized trials have failed to show an increase in new AF detection rates at 1 year using opportunistic screening with handheld-ECG devices when compared with usual practice, both in the US and the Netherlands. ^26–28^

Four trials have published results on clinical outcomes following AF screening. LOOP assessed the value of long-term (up to 3 years) AF screening with implantable loop recorders in 6,000 Danish people, aged 70 to 90 years, and with additional cardiovascular risk factors. ^60^ The LOOP investigators observed a nonsignificant 20% RRR in stroke or systemic arterial embolism over 5 years (HR 0.80 [0.61-1.05], *P* = .11). There was an unexpectedly high (4-fold greater) rate of new AF diagnosis in the control arm (12.2% compared to an anticipated 3%), possibly due to trial participation leading to altered behaviour in the control group, with high levels of opportunistic AF diagnosis in these high-risk individuals. The long and continuous monitoring in the active group might also have led to detection of mostly short AF episodes (the median AF burden was only 0.13% total over 3 years), ^61^ with an associated low stroke risk that might not definitively favour anticoagulation, in line with the results of NOAH-AFNET ^34^ and ARTESIA. ^35^

STROKESTOP assessed screening with twice-daily 30s single-lead ECG for 14 days by inviting all 28,768 people aged 75 or 76 years and living in the Halland and Stockholm regions in Sweden to participate, ^57^ and observed a 1.1% absolute reduction in the composite primary endpoint of stroke, systemic embolism, major bleeding, and all-cause death over 7 years (31.9% in the active group compared to 33.0% in the control group; HR: 0.96 [0.92-1.00], *P* =.045). The rate of participation in screening was 51%, and in a per-protocol analysis there was a 34% RRR in the secondary endpoint of ischemic stroke in individuals who participated in screening compared to the control group (HR: 0.66 [0.58-0.76], *P* < .0001), but there was no difference in the rate of ischemic stroke in an intention-to-treat analysis comparing the active and control groups (HR: 0.92 [0.83-1.01], *P* = .084). These results suggested that screening for AF may have a small net clinical benefit, but much larger trials would be needed to robustly demonstrate a reduction in ischemic stroke and to assess the extent to which this may be counter-balanced by any increase in bleeding.

The subsequent STROKESTOP-II trial randomized 28,712 individuals aged 75 or 76 years to risk-based screening (4x-daily 30s handheld-ECG for 14 days if point-of-care N-terminal pro-B-type natriuretic peptide [NT-proBNP] ≥125 ng/L, or a single 30s handheld-ECG if NT-proBNP <125 ng/L) or to a control group. ^62^ The participation rate in the intervention group was 49.2%, with no differences seen in AF rates or anticoagulation uptake either immediately after screening or at 5 years. There were also no differences in clinical outcomes, including the primary outcome of stroke or systemic embolism (HR: 0.96 [0.86-1.06], *P* = .412), ischemic stroke (HR: 0.94 [0.84-1.06, *P* = .324), or the same composite endpoint used in STROKESTOP (HR: 1.00 [0.95-1.05], *P* = .985). Importantly, the number of AF cases found was similar in both STROKESTOP studies. The authors noted lower stroke rates than expected, and suggest that longer monitoring duration and better screening uptake could have contributed to larger differences. However, more intense screening is unlikely to provide a meaningful clinical benefit (as now shown in LOOP, ARTESIA, and NOAH-AFNET). Moreover, the divergent results seen in both STROKESTOP studies despite similar AF rates further suggest that the clinical benefit seen in the original STROKESTOP trial may have been due to chance.

Finally, the GUARD-AF trial discussed previously had a target sample size of 52,000 and was powered to detect differences in hospitalization for stroke. ^31^ However, recruitment and follow-up were terminated early due to poor enrolment (following the COVID-19 pandemic) with 11,905 participants in the intention-to-treat population. Event rates were lower than expected, and only 71 out of 800 expected primary outcome events had accrued. After a median of 15.3 months, there was no difference in hospitalization for stroke (0.7% in screening arm vs 0.6% in usual care arm; HR: 1.10 [0.69-1.75]). This example highlights the importance of employing stream-lined recruitment and intervention approaches to preserve adequate statistical power and increase the likelihood of detecting what are likely to be small-to-moderate differences in AF detection and clinical event rates over the long-term. A streamlined and remote approach to undertaking AF screening has been trialled in the Norwegian Atrial Fibrillation self-screening pilot study, ^63^ and is being adopted in the ongoing NORSCREEN randomized trial, ^64^ which also obtains outcome data electronically from routine health records.

Taken together these results highlight that AF screening is not an intervention with proven benefits, and that further large RCTs are needed to establish whether systematic AF screening can prevent stroke. ^65^ The SAFER trial has completed randomization of 82,000 people aged 70 or older in primary care practices in England to screening with 4-times-daily single-lead ECG for 3 weeks versus usual care, and is expected to publish results on the primary outcome of stroke in 2027. However, given the small absolute event rates and risk reductions found in the STROKESTOP, ^57^ LOOP, ^60^ NOAH-AFNET, ^34^ ARTESIA, ^35^ and STROKESTOP II ^62^ trials, the statistical power required to conclusively demonstrate a clinically-relevant effect on clinical outcomes will probably need a larger and longer trial, along with pooling of all available randomized evidence, ^25^ and such a meta-analysis is already planned. ^20^ Such pooled data may also give sufficient statistical power to interrogate aspects of AF screening beyond stroke prevention, including other potential benefits (e.g. prevention of heart failure, risk factor modification) and potential harms (e.g. incidental findings, greater healthcare utilization).

If future studies are able to demonstrate a reduction in stroke risk, then systematic AF screening could be offered to large groups of patients (and is already being piloted in some healthcare settings). ^66,67^ Robust, randomized data will also be required to determine the most cost-effective screening approaches, which will be influenced by the incremental impact (on AF detection and costs) of a particular approach in a particular healthcare setting, and thus require separate dedicated assessments across multiple geographies. ^68^ Establishing cost-effectiveness will be key to further translation into clinical practice. ^69^ The development of artificial intelligence approaches to reporting ambulatory ECG data may help to reduce costs. ^70^

AMALFI is unlikely to have sufficient statistical power to assess the effects of systematic screening on stroke. However, it will generate randomized evidence on the long-term efficacy and cost-effectiveness of extended AF screening in UK clinical practice. So far, the trial has shown how a remote, streamlined, data-enabled approach can support recruitment and screening of thousands of patients (including doubling the initial sample size), and in the context of the global COVID-19 pandemic when another similar trial failed to recruit to target. ^31^ Moreover, the streamlined approach can also be easily scaled since all data needed for recruitment and evaluation are already collated centrally by NHSE. If efficacy and cost-effectiveness are confirmed, this methodology can be used to design a larger outcomes trial, and potentially form the basis of a future nationwide AF screening program.

## Conclusion

AF screening has been suggested as a potential way to reduce avoidable and debilitating cardioembolic strokes, but the available evidence is currently insufficient to recommend systematic AF screening. Data from randomized trials among different populations, with different screening schedules, and in diverse settings will be instrumental for any future recommendations. AMALFI will provide randomized evidence to establish the long-term efficacy and cost-effectiveness of remote AF screening with long-term continuous 14-day noninvasive ambulatory ECG monitoring among older patients at high cardiovascular risk in UK primary care practice.

## Supplementary Material

D&D_Appendix I

D&D_Appendix II

D&D_Appendix III

Definition&Derivation

Protocol

Sample_Report

SAP

Supplement

## Figures and Tables

**Table 1:** Baseline characteristics of randomized participants.

Characteristic	Overall (*N* = 5,040)	Randomized allocation	
		Patch (*N* = 2,520)	Control (*N* = 2,520)
Age at randomization (years)–n (%)			
*< 75*	1,366 (27.1%)	683 (27.1%)	683 (27.1%)
≥ 75, < 80	2,093 (41.5%)	1,032 (41.0%)	1,061 (42.1%)
≥ 80	1,581 (31.4%)	805 (31.9%)	776 (30.8%)
Mean (SD)	77.7 (6.0)	77.7 (5.9)	77.7 (6.0)
Female sex - n (%)	2,360 (46.8%)	1,180 (46.8%)	1,180 (46.8%)
Ethnicity – n (%) White	4,101 (81.4%)	2,045 (81.2%)	2,056 (81.6%)
Black	11 (0.2%)	2 (0.1%)	9 (0.4%)
Asian	56 (1.1%)	27 (1.1%)	29 (1.2%)
Mixed/Other	43 (0.9%)	21 (0.8%)	22 (0.9%)
Missing	829 (16.4%)	425 (16.9%)	404 (16.0%)
Body mass index (kg/m^2^)–n (%)			
< 25	1,003 (19.9%)	487 (19.3%)	516 (20.5%)
25 to < 30	1,534 (30.4%)	750 (29.8%)	784 (31.1%)
≥ 30	1,149 (22.8%)	573 (22.7%)	576 (22.9%)
Missing	1,354 (26.9%)	710 (28.2%)	644 (25.6%)
Mean (SD)	28.2 (5.2)	28.2 (5.2)	28.2 (5.2)
CHA_2_DS_2_VASc score–n (%)			
< 3	68 (1.3%)	33 (1.3%)	35 (1.4%)
3	1,359 (27.0%)	685 (27.2%)	674 (26.7%)
4	1,959 (38.9%)	983 (39.0%)	976 (38.7%)
≥ 5	1,654 (32.8%)	819 (32.5%)	835 (33.1%)
Median (IQR)	4 (3-5)	4 (3-5)	4 (3-5)
Prior diseases at randomization–n (%)			
Heart failure	503 (10.0%)	244 (9.7%)	259 (10.3%)
Hypertension	4,528 (89.8%)	2,255 (89.5%)	2,273 (90.2%)
Diabetes	1,432 (28.4%)	713 (28.3%)	719 (28.5%)
Stroke or TIA	963 (19.1%)	485 (19.2%)	478 (19.0%)
Thromboembolism	590 (11.7%)	271 (10.8%)	319 (12.7%)
Myocardial infarction	524 (10.4%)	269 (10.7%)	255 (10.1%)
Peripheral arterial disease	420 (8.3%)	214 (8.5%)	206 (8.2%)
Chronic kidney disease (stage ≥ 3)	901 (17.9%)	438 (17.4%)	463 (18.4%)
Medication prior to randomization–n (%)			
Oral anticoagulation	342 (6.8%)	174 (6.9%)	168 (6.7%)
Statin	3,476 (69.0%)	1,739 (69.0%)	1,737 (68.9%)
Aspirin or dipyridamole	1,379 (27.4%)	705 (28.0%)	674 (26.7%)
P2Y12 inhibition	683 (13.6%)	363 (14.4%)	320 (12.7%)
RAAS inhibition	3,144 (62.4%)	1,599 (63.5%)	1,545 (61.3%)
Beta-blockers	1,205 (23.9%)	619 (24.6%)	586 (23.3%)
Diuretics	1,422 (28.2%)	713 (28.3%)	709 (28.1%)
Calcium-channel blockers	2,489 (49.4%)	1,200 (47.6%)	1,289 (51.2%)
Insulin	230 (4.6%)	117 (4.6%)	113 (4.5%)
Proton-pump inhibitors or H2-antagonists	2,541 (50.4%)	1,265 (50.2%)	1,276 (50.6%)

HES-APC, hospital episode statistics admitted patient care; IQR, interquartile range; SD, standard deviation.

Age and sex from details on the randomization form. Ethnicity from primary care ethnicity/consultation records and HES-APC records. Body mass index from most recent record in the primary care past medical history records dated 0-3 years prior to randomization, ignoring extreme values <15 or >60kg/m^2^ . CHA_2_DS_2_VASc score based on age and sex (from the randomization form) and prior diseases at randomization, indicated by any prerandomization record of the disease in the primary care past medical history record or HES-APC record or self-reported on the randomization form. A small number of individuals (*n* = 68, 1.3%) have a reported CHA_2_DS_2_VASc score <3, likely due to changes to data coding in primary care between the time of the eligibility check and invitation (when Read codes were used) and the subsequent GP data extract (when SNOMED codes were used). Medication use at randomization indicated by any record of the medication in the national prescription record before or within the month of randomization, with insulin use also contributing to the definition of prerandomization diabetes. Only national data was available for 33 participants, only primary care data was available for 1 participant and no national or primary care linkage data was available for 7 participants.
