# Supplementary material for: Active Monitoring for AtriaL FIbrillation (AMALFI): Rationale, protocol, and pilot for a pragmatic, randomized, controlled trial of remote screening for asymptomatic atrial fibrillation
Source: Am Heart J. Author manuscript; Available in PMC 2026 Mar 10. (PMC7618845; doi:10.1016/j.ahj.2025.07.004)
Supplement: D&D_Appendix I [file EMS212696-supplement-D_D_Appendix_I.docx]

## Appendix I: Initial standardised electronic eligibility searches

Two eligibility searches were created on 4^th^ June 2019 for EMIS schema version 32.0.0 (one for males, one for females).

Purpose: to interrogate primary care data at participating GP practices with the aim of identifying potentially eligible participants.

Population exclusions (SNOMED, READV2 search codes in parentheses):

- Atrial fibrillation or flutter (300130013, G573)
- Senile and presenile organic psychotic conditions (401757012, E00)
- On gold standards palliative care framework (2534178018, 8CM1)
- On [V]Palliative care (460928017, ZV57C)
- Latex allergy (442112014, SN531)

Eligibility requires a CHA_2_DS_2_VASc score of 3 or more (for men) and 4 or more (for women). Therefore:

- those aged ≥75 years must score at least 1 point from the list of diseases below
- those aged 65-74 must score at least 2 points from the list of diseases below

Congestive heart failure (or Left ventricular systolic dysfunction) (1 point):

- Heart failure (139475013, G58)
- Left ventricular systolic (216207010, G5yy9)
- Echocardiogram shows left ventricular systolic dysfunction(2159197017, 585f)

Hypertension: blood pressure consistently above 140/90 mmHg (or treated hypertension on medication) (1 point):

- Hypertensive disease (64168014, G2)

Diabetes mellitus (1 point):

- Diabetes mellitus (121589010, C10)

Prior Stroke or TIA or thromboembolism (2 points):

- Cerebrovascular disease (104563015,G6), excluding
  - Subarachnoid haemorrhage (481028017, G60)
  - Intracerebral haemorrhage (744901000006114, G61)
  - Other and unspecified intracranial haemorrhage (300290016, G62)
  - Other cerebrovascular disease (300374018, G67)
- H/O: TIA (251692018, 14AB)
- Transient cerebral ischaemia (395788015, G65), excluding
  - Basilar artery syndrome (106392017, G650)
  - Vertebral artery syndrome (58046010, G651)
  - Subclavian steal syndrome (25897016, G652)
  - Transient global amnesia (95961000006119, G655)
  - Vertebrobasilar insufficiency (106394016, G656)
- Thromboembolism
  - H/O: thrombo-embolism (217601000000119, 14A8)
  - Thromboembolic pulmonary hypertension(350517010, G41y1)
  - Pulmonary embolism (98484016, G401)
  - [V] Personal history of pulmonary embolism (451479013, ZV129)
  - [RFC] Pulmonary embolism/pulmonary hypertension (905451000006118, HNG0019)
  - [RFC] Arterial embolism of limbs (905541000006119, HNG0028)
  - Cerebral embolism (125470015, G641)
  - Embolism and thrombosis of an arm or leg artery (300534011, G742), excluding
    - Post radiological embolism of upper limb artery (216711000006118, G742A)
    - Post radiological embolism of lower limb artery (216701000006116, G742B)

Vascular disease (e.g. peripheral artery disease, myocardial infarction, aortic plaque) (1 point)

- Acute myocardial infarction (94884017, G30)
- Old myocardial infarction (4031011, G32)
- Subsequent myocardial infarction (299808017, G35)
- Certain current complication follow acute myocardial infarct (543291000006110, G36)
- Postoperative myocardial infarction (208365015, G38)
- Acute coronary syndrome (1488382011, G3115)
- Other peripheral vascular disease (395791015, G73) excluding
  - Upper limb ischaemia (350540014, ESCTUP1)
  - Raynaud's syndrome (192131000006114, G730)
  - Thromboangiitis obliterans (87208017, G731)
  - HAVS - Hand-arm vibration syndrome (2263901000000118, G735)
- Peripheral vascular disease NOS (235911000006116, G73z) excluding
  - Spasm of peripheral artery(132581000006113, G73z1)
