# Supplementary material for: Active Monitoring for AtriaL FIbrillation (AMALFI): Rationale, protocol, and pilot for a pragmatic, randomized, controlled trial of remote screening for asymptomatic atrial fibrillation
Source: Am Heart J. Author manuscript; Available in PMC 2026 Mar 10. (PMC7618845; doi:10.1016/j.ahj.2025.07.004)
Supplement: Definition&Derivation [file EMS212696-supplement-Definition_Derivation.docx]

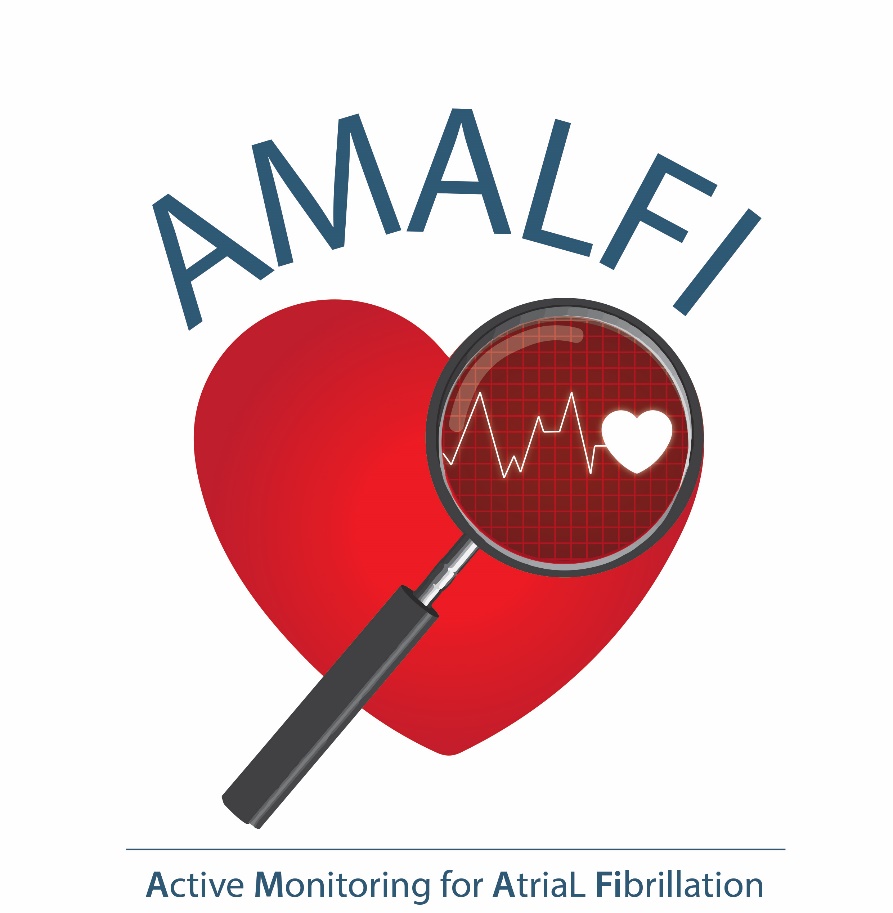


Definition and Derivation of Baseline Characteristics and Outcomes

Version 1.2

Date: 26 June 2025

Aligned with protocol v2.0 and statistical analysis plan v1.2

IRAS no: 234837

REC ref: 19/LO/0220


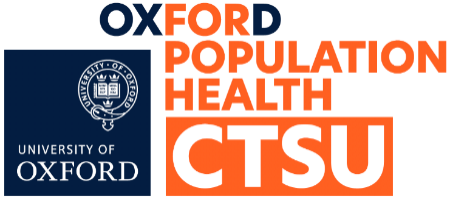
ISRCTN: 15544176

Contents

[1 Version 2](#_Toc195787176)

[2 Scope 3](#_Toc195787177)

[3 Abbreviations 3](#_Toc195787178)

[4 Data sources 3](#_Toc195787179)

[4.1 Randomisation questionnaire 3](#_Toc195787180)

[4.2 Zio XT patch data 4](#_Toc195787181)

[4.2.1 Urgent reports sent to GP 4](#_Toc195787182)

[4.3 Primary care data 4](#_Toc195787183)

[4.3.1 Dataset structure 6](#_Toc195787184)

[4.4 NHS datasets 8](#_Toc195787185)

[4.4.1 Office for National Statistics (ONS) Mortality data 8](#_Toc195787186)

[4.4.2 Medicines Dispensed in the Community \ NHS Business Services Authority (NHSBSA) data 9](#_Toc195787187)

[4.4.3 Hospital Episode Statistics Admitted Patient Care (HES-APC) 9](#_Toc195787188)

[4.4.4 HES Critical Care (HES-CC) 10](#_Toc195787189)

[4.4.5 HES Outpatient Care (HES-OP) 10](#_Toc195787190)

[4.4.6 Emergency Care Data Set (ECDS) 10](#_Toc195787191)

[4.5 Quality of life questionnaires (EQ-5D-5L) 10](#_Toc195787192)

[4.6 Withdrawal of consent 11](#_Toc195787193)

[5 Baseline characteristics 11](#_Toc195787194)

[6 Outcomes 12](#_Toc195787195)

[6.1 Atrial fibrillation diagnoses (primary, secondary, and exploratory outcomes) 12](#_Toc195787196)

[6.2 Oral anticoagulation (exploratory outcomes) 12](#_Toc195787197)

[6.3 Other clinical outcomes 13](#_Toc195787198)

[7 Other descriptive findings 13](#_Toc195787199)

[7.1 Adherence to patch intervention 13](#_Toc195787200)

[7.2 Cardiac monitoring findings (detected through patch) 13](#_Toc195787201)

[8 Length and completeness of follow-up 14](#_Toc195787202)

[8.1 Death (fact and date) 14](#_Toc195787203)

[8.2 Loss to follow-up in primary care records (known change of GP practice) 14](#_Toc195787204)

# Version

| **Date** | **Version** | **Comments** | **Authors** |
| --- | --- | --- | --- |
| 17/03/2025 | 1.0 | Initial version | Guilherme Pessoa-Amorim, Georgina Buck, Charlie Harper |
| 17/04/2025 | 1.1 | Revised version | Guilherme Pessoa-Amorim, Georgina Buck, Charlie Harper, Rohan Wijesurendra |
| 26/06/2025 | 1.2 | Revised version | Guilherme Pessoa-Amorim, Georgina Buck, Charlie Harper, Rohan Wijesurendra |

# Scope

This document describes the definition and derivation of baseline characteristics and of primary, secondary, and other outcomes of the AMALFI trial for published trial analyses. It should be read alongside the study protocol, which outlines the study design and provides a short definition of trial outcomes, as well as the Statistical Analysis Plan (SAP) which describes the statistical methods to be used to analyse the trial outcomes. The SAP refers to this document in Section 3.6.4.

# Abbreviations

| ACEi | Angiotensin-Converting-Enzyme inhibitors |
| --- | --- |
| AF | Atrial fibrillation |
| ARB | Angiotensin Receptor Blockers |
| ARNi | Angiotensin-receptor/neprilysin inhibitors (ARNi) |
| AVB | Atrioventricular block |
| BMI | Body mass index |
| DVT | Deep vein thrombosis |
| ECDS | Emergency Care Data Set |
| HES | Hospital Episode Statistics |
| HES-APC | HES Admitted Patient Care |
| HES-CC | HES Critical Care |
| HES-OP | HES Outpatients |
| GLP1RA | Glucagon-Like Peptide-1 receptor agonists |
| FCE | Finished Consultant Episode |
| GP | General practice |
| ICD-10 | International Classification of Diseases, Tenth Revision |
| NHS | National Health Service |
| NHSBSA | NHS Business Services Authority |
| ONS | Office for National Statistics |
| OPCS-4 | OPCS Classification of Interventions and Procedures version 4 |
| PE | Pulmonary embolism |
| RAAS | Renin-angiotensin-aldosterone system |
| SAP | Statistical analysis plan |
| SGLT2i | Sodium-glucose transporter 2 inhibitors |
| SVT | Supraventricular tachycardia |
| TIA | Transient ischaemic attack |
| VT | Ventricular tachycardia |

# Data sources

## Randomisation questionnaire

A standardised electronic search of the primary care data available at each participating GP practice (see Appendix I) was used to identify potentially eligible trial participants. Information on patient demographics and contact details were then extracted from electronic primary care records and used to generate and mail an invitation to the study to potentially eligible trial participants. Recipients could choose whether or not they wished to respond. The randomisation questionnaire that accompanied the study invitation was completed by those interested to take part and sent to the trial team (prior to randomisation). This questionnaire included information on self-reported medical history (focused on the components of the CHA_2_DS_2_VASc score and history of allergy to latex). Responses to the following medical history questions were used to inform the minimisation randomisation algorithm and will be used in combination with primary care and other linkage data sources to define baseline characteristics.

- Heart failure or inadequate heart pumping (not angina, stents or pacemakers)
- Hypertension or high blood pressure, or on treatment for your blood pressure
- Type 1 or type 2 diabetes (other than during pregnancy) or on treatment to control your blood sugar
- Confirmed (rather than suspected) stroke or mini-stroke (sometimes called TIA)
- Blood clot in your legs or lungs (known as DVT or PE)
- Told by a doctor that you have poor blood flow in the arteries in your legs (known as peripheral vascular disease)
- Heart attack

The details printed on the randomisation questionnaire (from data extracted from the primary care records) were used as an indirect source of patient demographics and contact details from primary care records. The following participant information was collected using this pipeline:

- First name, family name
- NHS number
- Date of birth
- Sex (male/female)
- GP practice

## Zio XT patch data

Participants in the active arm of the trial were mailed a cardiac monitoring device (Zio XT, iRhythm Technologies, San Francisco, CA, USA) to self-apply and wear for a target of 14 days. Data from the Zio XT were used to extract information on the presence and characteristics of atrial fibrillation (AF) episodes, other abnormal cardiac rhythm findings during the monitoring period and adherence to wearing the device.

### Urgent reports sent to GP

An urgent report was emailed to the participant’s GP by the study team in cases where AF or other findings considered to be clinically relevant (i.e. atrial flutter, sustained ventricular tachycardia or fibrillation; pauses lasting over 6s; complete heart block, Mobitz type II, or high grade atrioventricular block) were found.

## Primary care data

Focused extracts of primary care records will be extracted from each of the participating GP practices at two time points; firstly at least 2.5 years, and secondly at least 5 years, both after the date of randomisation of the last participant randomised at each practice. These extracts are performed using a standardised electronic search, which can be uploaded to the EMIS electronic medical record (used for all practices taking part in the trial). The search was developed and tested by two trial investigator GPs, and will be the same used across all practices. The search will be reviewed before extraction at each time point.

The primary care data extracts are split into 5 files covering distinct characteristics of interest, as follows:

- Past medical history (“Report on AMALFI PMH”):
  - Smoking
  - Body mass index (BMI)
  - Alcohol consumption
  - Atrial Fibrillation (AF)
  - Supraventricular tachycardia (SVT)
  - Ventricular tachycardia (VT)
  - Atrioventricular block (AVB)
  - Heart failure
  - Stroke or transient ischaemic attack (TIA)
  - Diabetes mellitus
  - Peripheral arterial disease
  - Myocardial infarction
  - Hypertension
  - Chronic kidney disease
  - Obstructive sleep apnoea
  - Chronic obstructive pulmonary disease
  - Deep vein thrombosis (DVT) or pulmonary embolism (PE)
  - Bleeding
  - Dementia
  - Death
- Medications 1 (“Report on AMALFI Meds, Anticoags to RAS Inhibs”):
  - Oral anticoagulation
  - Aspirin
  - Statins
  - Beta-blockers
  - Digoxin
  - Amiodarone
  - Flecainide
  - Non-dihydropyridine calcium channel blockers
  - Diuretics
  - Mineralcorticoid receptor antagonists
  - Angiotensin-receptor/neprilysin inhibitors (ARNi)
  - Sodium-glucose transporter 2 inhibitors (SGLT2i)
  - Renin-angiotensin-aldosterone system (RAAS) inhibitors (Angiotensin-Converting-Enzyme inhibitors [ACEi]/Angiotensin Receptor Blockers [ARB])
- Medications 2 (“Report on AMALFI Meds, Insulins to Vasodilators CCBs”):
  - Insulins
  - Oral antidiabetic drugs
  - Injectable hypoglycaemic medications (non-insulin) – i.e. injectable Glucagon-Like Peptide-1 receptor agonists (GLP1RA)
  - Ezetimibe
  - Proprotein convertase subtilisin/kexin type 9 inhibitors
  - Fibrates
  - P2Y12 receptor blockers
  - Nitrates
  - Dihydropyridine calcium channel blockers or nitrates
- Referrals/investigations/interventions/symptoms (“Report on AMALFI Referrals, Investigations, Interventions, Symptoms”):
  - Referrals: cardiology
  - Referrals: others
  - Investigations: echocardiography
  - Investigations: electrophysiological study
  - Interventions: direct current cardioversion
  - Interventions: cardiac ablation
  - Interventions: pacemaker or implantable cardioverter/defibrillator
  - Symptoms: angina
  - Symptoms: palpitations
  - Symptoms: dizziness
  - Symptoms: shortness of breath
  - Symptoms: fatigue
  - Symptoms: oedema
- Ethnicity/consultations (“Report on AMALFI Ethnicity, Consults”)
  - Ethnicity
  - Consultations

### Dataset structure

The print-screen below shows the typical structure of each extract file (past medical history in this case).


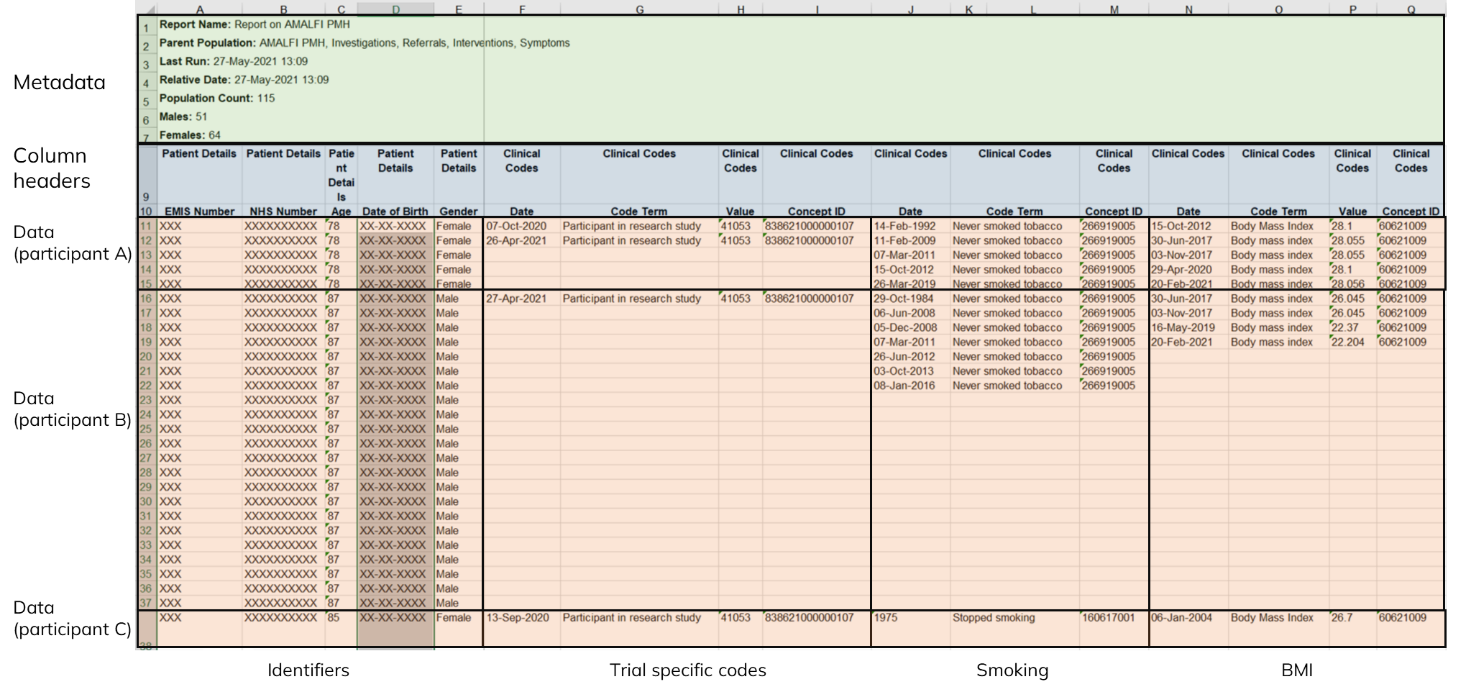


Participant 1

The first 8 rows show report metadata (row 8 is shrunk but present). An additional row may be present here for some practices where patient data was hidden from the export due to confidentiality reasons (this is a block created in the underlying electronic medical record which cannot be overcome). Row 9 shows “column group” (“Patient Details” for columns A-E and “Clinical Codes” for the remaining columns). Row 10 is the actual column header. Actual data starts from row 11 onwards. Each row includes participant’s EMIS number, NHS number, date-of-birth, age at the time of data extraction, and gender (columns A-E, some redacted in the above print-screen) (with these data only being used for linkage purposes). Data from 3 different participants is shown here.

Extracted data is included from column F onwards. Each “column group” - i.e. a sequence of 3-4 columns (5 for medication records) - depicts information for a characteristic of interest. These typically include record date (“Date”), SNOMED code description (“Code term”), and SNOMED code number (“Concept ID”) for each relevant record found in the primary care data (see below for lists of extracted codes). Some extract files include outputs in other formats (as further specified below). Some characteristics can also include an associated value (e.g. alcohol, BMI). Medication prescription records include full description, record date, number of individual items included in the prescription, number of prescriptions issued, and instructions for use (posology).

As shown above, columns F-I include the AMALFI study-specific codes added to the primary care record, which flagged an individual as taking part in the trial and allowed their inclusion in the data extract (SNOMED code 838621000000107, with description “Participant in research study”, and associated study-specific value of “41053”). Columns J-M include data on smoking (note the merged columns K-L). Columns N-Q include data on BMI. A link to the description of all columns is provided below.

The number of rows for each participant depends on the characteristic/medication/event with the largest number of records, as shown in the example print-screen below.


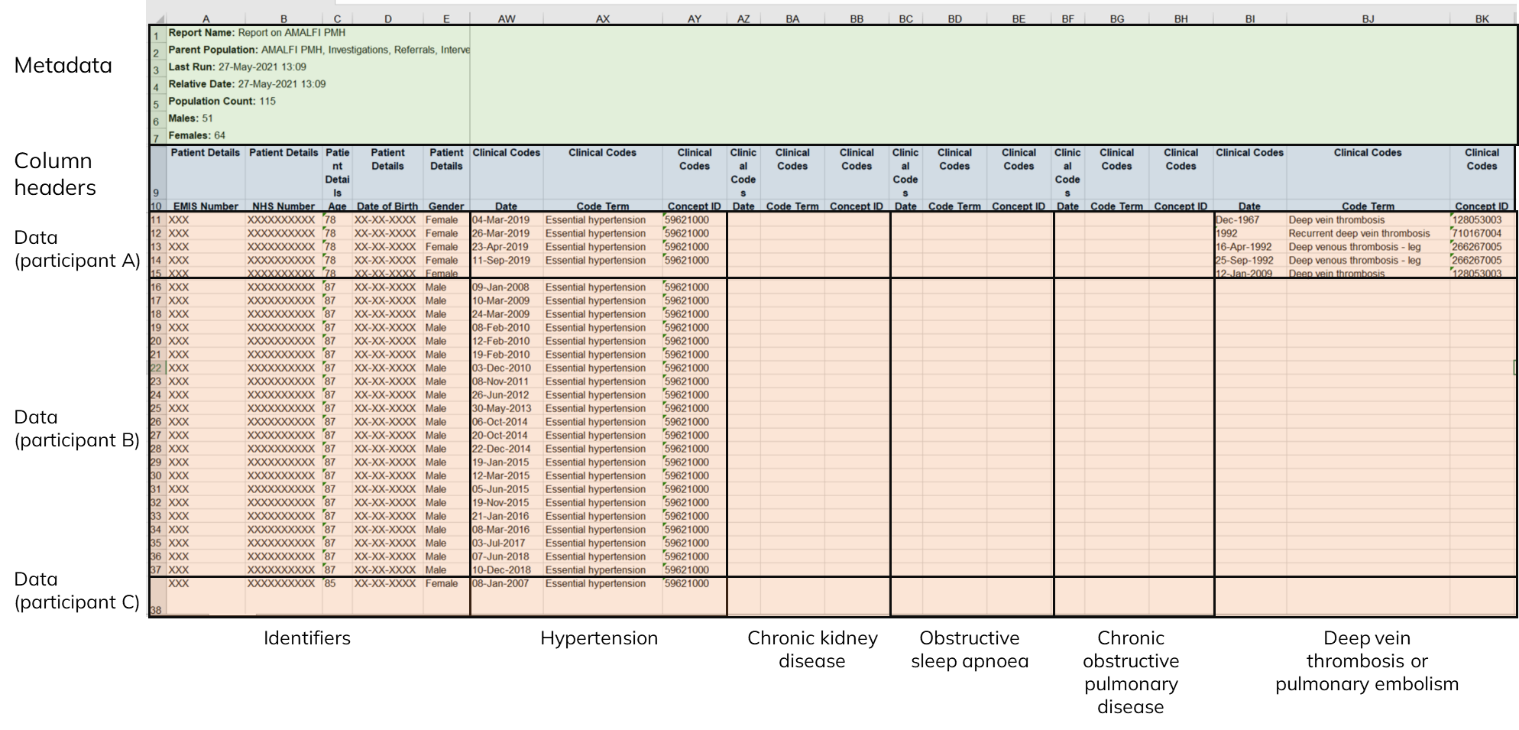


For participant A, the number of rows depends on the characteristic “Deep vein thrombosis or pulmonary embolism”, which has five records. This creates empty rows for characteristics for which there are no relevant records for this participant (e.g. “Hypertension”, “Chronic kidney disease”, “Obstructive sleep apnoea”), but which may hold data for other participants. Similarly, for participant B, the number of rows depends on the characteristic “Hypertension”.

Note that the meaning of each column group is not apparent from the data itself. While this can be inferred from the data included in each column group, this would not be possible in the absence of a relevant record (as exemplified above for “Chronic kidney disease”, “Obstructive sleep apnoea”, and “Chronic obstructive pulmonary disease”). For this purpose, machine-readable descriptions of each column included in the raw data extracts at 2.5 years are provided in Appendix II (tabs starting “GP Report”). These files should be used as guidance when applying any transformations to the raw data given the non-standard structures, which can vary from one extract type to the other, and include idiosyncrasies such as merged columns and different numbers of columns for different characteristics within the same file (but which are replicable across extracts from different GP practices and time points). As mentioned above, the number of metadata rows at the top of each file may also change, but the row containing actual column headers can be identified by looking for the row where column A is “EMIS Number”.

Dates can be provided as either day-month-year, month-year, or year only.

Machine-readable codelists depicting the codes (and respective terminologies) used to interrogate the primary care record using the electronic search and produce the output extracts at 2.5 years are provided in Appendix II (“GP extract codelist” tab). While most codes come from the SNOMED terminology, some characteristics may be coded using SNOMED-like codes (i.e. EMIS implementations using codes similar to SNOMED, for which no publicly available dictionaries are available) or not include any medical terminology code (i.e. only a description term). Therefore, depending on the characteristics of interest, record handling and interrogation must be performed using either a combination of SNOMED code + description, or description only.

Codes returned in the primary care record tend to be more specific than those used to interrogate the records. A complete list of the codes contained in the primary care data received at 2.5 years are provided in Appendix II (“GP received codes” tab). All returned codes will be reviewed at each extraction to confirm that they are relevant to confirming or refuting the presence of the characteristic of interest (for example if the characteristic of interest was heart failure a returned code of 414072005 [Echocardiogram shows normal left ventricular function] would not be relevant to confirming or refuting the diagnosis, despite it being of clinical relevance).

## NHS datasets

### Office for National Statistics (ONS) Mortality data

ONS mortality date (also known as “Civil Registrations”) includes data from official records for all deaths registered in England and Wales. The following data are provided:

- Date of death
- Date of death recording
- Underlying cause of death
- Contributory causes of death
- Other conditions recorded on the death certificate but not contributing to death
- Whether a post-mortem took place

ONS data will be the main data source used to identify date and cause of death. Clinical data are recorded using ICD-10 codes.

### Medicines Dispensed in the Community \ NHS Business Services Authority (NHSBSA) data

The Medicines Dispensed in the Community dataset is a data collection assembled by NHSBSA, containing all medications prescribed in the community setting in England, and submitted for reimbursement in England/ Scotland/ Wales/Isle of Man/ Jersey/ Guernsey/ Alderney since April 2018. Submission to NHSBSA by pharmacies and other dispensing bodies is compulsory for reimbursement. The underlying event captured is medication dispensing, and the following information is included:

- Calendar month when dispensing event was submitted for reimbursement (i.e. there are no exact dates)
- SNOMED and British National Formulary (BNF) codes and descriptions
- Quantity dispensed
- Strength of individual item dispensed (NB not daily dose)
- Formulation

The data include separate “paid” and “prescribed” columns. “Paid” fields are the original information captured by NHSBSA (as the data collection is built from reimbursement claims), with “prescribed” fields extracted directly from the electronic prescription (or inferred in some circumstances for paper prescriptions). Prescribed fields should be used for all analyses of medication use/exposure, with paid fields reserved for health economic analyses.

NHSBSA data will be used to identify background exposure to medications of interest prior to randomisation, and to identify exposure to oral anticoagulation during follow-up.

### Hospital Episode Statistics Admitted Patient Care (HES-APC)

HES-APC contains data on admissions to NHS hospitals in England, which aims to enable reporting and analyses to support the NHS in the delivery of healthcare services. These data are submitted on a regular basis by NHS hospital trusts at pre-arranged dates during the year and included in HES-APC after consolidation, validation, and cleaning. Each record contains data relating to a continuous period of care under one consultant known as a Finished Consultant Episode (FCE). FCEs can be grouped together to form ‘Spells’. Each spell is a continuous period of inpatient care within one hospital. Each FCE contains data about the patient (e.g. sex, ethnicity), the specialty providing the care (e.g. cardiology), ICD-10 diagnostic and OPCS-4 procedure codes, along with dates for each procedure and details about the admission and discharge and other data. HES-APC data is available since the 2014-2015 financial year.

HES-APC data will be used to extract information on ethnicity, to identify prior medical conditions using ICD-10 and OPCS-4 codes, and to identify clinical events of interest during follow-up (including stroke, bleeding, and death). Specifically, the following data will be used to define baseline characteristics/medical history, and also for the diagnosis of AF in sensitivity analyses:

- Ethnicity
- Start and end date of each FCE
- Diagnoses recorded during FCE (ICD-10 coded)
- Procedures performed during FCE (OPCS-4 coded) and corresponding dates

HES-APC data will also be used for health-economic analyses, which are outside the scope of this document and will be further specified separately.

### HES Critical Care (HES-CC)

HES-CC is composed from the same data submission pipelines that generate HES-APC, but includes information only on critical care admissions. These data include information on admission source and type, dates of admission and discharge, status at discharge, type of critical care unit, and number of days an individual received different types of critical support (e.g. cardiovascular, respiratory). HES-CC data are available for trial participants from the 2014-2015 financial year onwards.

HES-CC data are only planned to be used for health-economic analyses, which are outside the scope of this document and will be further specified separately.

### HES Outpatient Care (HES-OP)

Similarly to HES-CC, HES-OP is also composed using the same reporting infrastructure as HES-APC but is focused only on outpatient care (in secondary care facilities). This dataset has a similar structure to that of HES-APC. HES-OP data are available for trial participants from the 2014-2015 financial year onwards.

HES-OP data are only planned to be used for health-economic analyses, which are outside the scope of this document and will be further specified separately.

### Emergency Care Data Set (ECDS)

ECDS is a national dataset focused on urgent and emergency care, and replaces the previous Accident and Emergency Commissioning Data Set. The dataset includes information on comorbidities, diagnoses, treatments, and investigations recorded, and is available for trial participants since the 2017-2018 financial year.

ECDS data are only planned to be used for health-economic analyses, which are outside the scope of this document and will be further specified separately.

## Quality of life questionnaires (EQ-5D-5L)

Health-related quality of life data is being collected using standardised EQ-5D-5L questionnaires. These are to be mailed to all trial participants at the same time, and at two time points:

1. After trial recruitment ended (undertaken in September 2022)
2. When the main trial results are published (scheduled for August 2025)

These data will only be used for health-economic analyses, which are outside the scope of this document.

## Withdrawal of consent

Participants are able to notify the study team directly if they wish to withdraw from the trial. Four types of withdrawal are recorded in the study database.

- Complete withdrawal
- Withdrawal from primary care data sharing
- Withdrawal from NHS England data sharing
- Withdrawal from direct contact

Participants who withdraw their consent for primary care or NHS England data sharing, or who withdraw consent completely, will be censored at the date of withdrawal. Those who withdraw from direct contact only will not be contacted further by the study team, but their data will contribute to analyses.

# Baseline characteristics

Participant identifiers including sex and date of birth are extracted from the details printed on the randomisation questionnaire (i.e. they originate from the primary care record). Other baseline characteristics (at randomisation) are extracted from primary care, self-report on the randomisation questionnaire, HES-APC, and NHSBSA data. These will include:

- Age at randomisation
- Sex
- Ethnicity (split into White, Black, Asian, Mixed/Other, and missing)
- BMI (numeric value, and split by groups of <25, 25 to <30, ≥30 kg/m^2^, and missing)
- CHA_2_DS_2_VASc score
- CHA_2_DS_2_VASc components
- Chronic kidney disease (stage 3+)
- Use of oral anticoagulation (excluding rivaroxaban 2.5mg BD)
- Use of other relevant treatments:
  - Statins
  - Aspirin or dipyridamole
  - P2Y12 inhibition
  - RAAS inhibition (ACEi/ARB/ARNi)
  - Beta-blockers
  - Diuretics
  - Calcium-channel blockers (diltiazem, verapamil, dihydropyridines)
  - Insulin
  - Other anti-diabetes medications (excluding SGLT2i and GLP1RA)

In general terms, clinical conditions at baseline will be identified based on the presence of a relevant record at any time previous to randomisation in either primary care or HES-APC data, or if self-reported on the randomisation questionnaire. Diabetes at baseline will also include exposure to insulin at any time prior to or in the month of randomisation. BMI will be identified based on records within the 3 years prior to randomisation within primary care data, and considering only values >15 and <60 kg/m^2^. Ethnicity will be based on records at any time point prior to randomisation and up to 2.5 years after, within primary care or HES-APC data (codelists detailed in Appendix II [“Codelists ethnicity” tab], discrepancies managed using the procedure specified in Appendix III). Medication exposure prior to randomisation will be based on the presence of a relevant record in NHSBSA data only, between the commencement of the NHSBSA database in April 2018 up to and including the month of randomisation.

Incomplete dates can be used as long as they are clearly pre-randomisation (with day 15 imputed in records with missing day, and June imputed in records with missing month). In cases where an incomplete date is potentially post-randomisation an approximate date will be assigned based on clinician review (blinded to treatment arm) of the HES-APC, primary care diagnoses, and NHSBSA data.

The detailed implementation algorithm for derivation of each of these parameters is specified in Appendix III. Relevant codelists are detailed in Appendix II (“Codelists clinical conditions” tab and tabs starting “Codelist Med”).

# Outcomes

## Atrial fibrillation diagnoses (primary, secondary, and exploratory outcomes)

The primary and secondary trial outcomes, as well as some exploratory outcomes, are focused on post-randomisation recording of AF in primary care records. Extraction of this endpoint (fact and date of AF diagnosis) will be based on the presence of a record within the relevant columns in the raw primary care data, as specified in Appendix II (“GP report PMH” tab), and within the specific analysis period (i.e. within 913 days or 1826 days after randomisation for outcomes analysed at 2.5 and 5 years, respectively). Dates will be extracted from the relevant primary care record. The initial eligibility search excluded participants with a primary care record of AF, however it is possible for AF to be recorded in the window between the search and randomisation (and for records to be backdated to before randomisation). Participants with an AF record preceding randomisation will only be counted as having AF after randomisation if a distinct, subsequent post-randomisation record is also found. Counts of participants with a pre-randomisation record of AF in primary care records will also be computed. In cases where an incomplete date of AF is potentially post-randomisation, an approximate date will be assigned based on clinician review (blinded to treatment arm) of the HES-APC, primary care diagnoses and NHSBSA data.

Identification of AF records in HES-APC will be required for sensitivity analyses only, following the same approach outlined above (and further specified in Appendix III).

## Oral anticoagulation (exploratory outcomes)

Information on oral anticoagulation use is to be extracted from NHSBSA (Dispensing) data only. See Appendix II (“Codelist Med (Oral anticoag” tab) for the codelist of included oral anticoagulants. Details on the technical implementation of these outcomes are provided in Appendix III.

Exposure to oral anticoagulation (excluding rivaroxaban 2.5mg BD) is an exploratory outcome. Analyses of this outcome within the specific analysis period (i.e. within 30 months or 60 months after randomisation for outcomes analysed at 2.5 and 5 years respectively) will include three distinct estimates:

- Binary post-randomisation exposure (yes/no)
- Time to first post-randomisation record (in calendar months)
- Cumulative post-randomisation exposure (total number of distinct calendar months with evidence of likely exposure to oral anticoagulation, defined as any months with a relevant oral anticoagulation record, or months between oral anticoagulation records where the interval between records was 3 months or less); this definition followed an exploratory assessment of usual anticoagulation dispensing records which showed that, although oral anticoagulants were most frequently dispensed every month, the median interval between consecutive dispensing events for each participant was two calendar months (i.e. a prescription every 2 months), and a substantial minority had a prescription every 3 months. A schematic illustration of the intended calculations is shown in the table below.

| **Participant** | **Month 1** | **Month 2** | **Month 3** | **Month 4** | **Month 5** | **Month 6** | **Month 7** | **Month 8** | **Total months on anticoagulation** |
| --- | --- | --- | --- | --- | --- | --- | --- | --- | --- |
| **1** | AC | AC | AC | AC | AC | AC | AC |  | 7 |
| **2** | AC |  | AC |  | AC |  | AC |  | 7 |
| **3** | AC |  |  | AC |  |  | AC |  | 7 |
| **4** | AC |  |  |  | AC |  |  |  | 2 |
| **5** | AC | AC |  | AC |  |  | AC |  | 7 |
| **6** | AC |  | AC |  |  |  | AC |  | 4 |

*Months where oral anticoagulation exposure is assumed are marked in grey; AC – oral anticoagulation record*

## Other clinical outcomes

Long-term assessments of clinical outcomes (including hospitalisation, stroke, bleeding, and death) are currently planned as exploratory assessments only, and will be specified at a later stage.

# Other descriptive findings

## Adherence to patch intervention

Details on adherence to patch intervention will be reported for participants in the active arm, including:

- Numbers wearing patch (yes/no)
- Length of time patch was worn (in days)
- Proportion of patch wear time with analysable data
- Time from randomisation to patch activation (in days)

## Cardiac monitoring findings (detected through patch)

Heart rhythm findings detected by the ECG patch will be reported for participants in the active arm of the study who wore a patch, and can be extracted directly from the patch data loaded into the study database. These will include:

- AF detection (detected/not detected)
- Characteristics of AF detected via patch
  - Burden (i.e. proportion of time spent in AF during the monitoring period)
  - Longest AF episode duration (in hours)
  - Maximum heart rate during AF episodes
  - Minimum heart rate during AF episodes
  - Time to first AF episode during the monitoring period (in days)
  - Time between date of AF reporting to the GP (extracted from the date the urgent patch report was emailed to the study team, as they are assumed to be on the same day) and the date of the first AF record in primary care records (in days)
- Presence of other arrhythmias found through monitoring
  - Atrial flutter, manually extracted from PDF reports
  - Supraventricular tachycardias (SVT), as extracted from the patch monitoring data
  - Non-sustained ventricular tachycardias (VT) - defined as VT lasting less than 30 seconds, as extracted from the patch monitoring data
  - Sustained VT – defined as VT lasting more than 30 seconds, as extracted from the patch monitoring data
  - Pauses lasting over 6 seconds, as extracted from the patch monitoring data
  - Atrioventricular (AV) block (2^nd^ degree Mobitz II/high-grade/complete heart block), manually extracted from PDF reports.

Further details on the derivation of these endpoints are provided in Appendix III.

# Length and completeness of follow-up

Definitions of length and completeness of follow-up will vary depending on the specific outcome being assessed, as they rely on different datasets. These are outlined in detail in the SAP.

In order to calculate these estimates, the following events must be computed from the available data sources:

- Death
- Loss-to-follow-up in primary care records (known change of GP practice)

Withdrawal of consent will also be used to define length/completeness of follow-up (see section 4.6), and can be readily extracted from the study database.

## Death (fact and date)

Information on fact and date of death can be extracted from two sources: ONS mortality data and HES-APC (primary care data also contain information on death, but exploratory data analysis revealed it not to be reliable). However, only ONS data will be used for this purpose as they are the official national death registry, and trial analyses are only planned at large time intervals (which should allow all death events occurring during the time period of interest to be recorded in the official registry).

## Loss to follow-up in primary care records (known change of GP practice)

Primary care data can only be collected if trial participants remain registered and interacting with the GP practice in which they were recruited to the trial, or if they move to another GP practice also taking part in the trial (as the data extraction procedure is the same and is triggered by the presence of a study specific code, which should be copied across when records are moved from one practice to the other).

Censoring of outcomes based on collection of primary care data therefore requires identification of changes to GP practice. These can be notified by participants directly to the trial team and are therefore recorded in the trial database. An alternative method is to identify a change in GP practice code attached to dispensing records in the NHSBSA data (field *PatientGPODS*). The study population comprises individuals aged 65+ years, with at least one comorbidity, so that >99% of study participants have a dispensing record in NHBSA data. Medication dispensing in England usually occurs monthly or every 2-3 months (as identified from exploratory data analysis), and the data pipelines underlying the NHSBSA dataset are subject to extensive data quality controls. It is therefore expected that the GP practice code assigned to dispensing records should provide a reasonable surrogate to identify change of GP practice, with a temporal accuracy of approximately 30 days.

**Loss to follow-up in primary care records** due to change of GP practice (to a practice not taking part in AMALFI) will therefore be defined as earlier of:

- the date in which a new GP practice not taking part in the AMALFI trial is registered in the study database (using the date in which the new record was created), or
- the first day of the month in which a GP practice code for a GP practice not taking part in AMALFI first appears in NHSBSA data after randomisation (with the monthly processing period (in the format yyyymm) to be extracted from digits 2-7 in *BSAPrescriptionID*, as for other data items in NHSBSA data), as shown in the screenshot below.


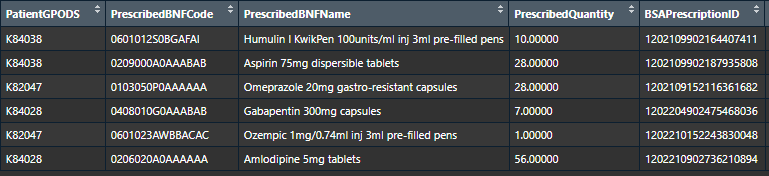


A list of GP practices taking part in AMALFI is included in Appendix II (“Participating GP Practice” tab).
