# Supplementary material for: Active Monitoring for AtriaL FIbrillation (AMALFI): Rationale, protocol, and pilot for a pragmatic, randomized, controlled trial of remote screening for asymptomatic atrial fibrillation
Source: Am Heart J. Author manuscript; Available in PMC 2026 Mar 10. (PMC7618845; doi:10.1016/j.ahj.2025.07.004)
Supplement: Protocol [file EMS212696-supplement-Protocol.pdf]

AMALFI: Active Monitoring for AtriaL Fibrillation

A randomized controlled trial of screening for silent atrial fibrillation in high-risk individuals.

Ethics Ref: 19/LO/0220
Date and Version No: 2021-06-08 V2.0

Chief Investigator: Professor Louise Bowman

Investigators: Professor Barbara Casadei
Dr Guilherme Pessoa-Amorim
Dr Nicholas Jones
Dr Christine A'Court
Mr Richard Bulbulia
Mrs Georgina Buck

Sponsor: University of Oxford

Funder: NIHR, Oxford Biomedical Research Centre (BRC)

Chief Investigator Signature:

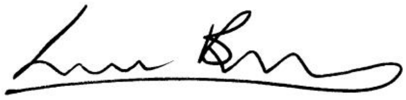

AMALFI Central Coordinating Office, Clinical Trial Service Unit,
Richard Doll Building, Old Road Campus, Roosevelt Drive, Oxford OX3 7LF, UK

Tel: Freephone 0808 164 5080
or: +44 (0) 1865 743884

Email: amalfi@ndph.ox.ac.uk, website: www.amalfitrial.org

TABLE OF CONTENTS

1. SYNOPSIS 4
2. ABBREVIATIONS 5
3. BACKGROUND AND RATIONALE 6
3.1 AF: A global health problem associated with substantial morbidity and mortality 6
3.2 Detecting SCAF 6
3.3 Is stroke risk different in SCAF? 7
3.4 Wilson & Jungner criteria for screening 7
3.5 Approaches to screening for SCAF 8
4. STUDY AIMS 9
5. STUDY DESIGN 10
6. PARTICIPANT IDENTIFICATION 11
6.1 Study Participants 11
6.2 Inclusion Criteria 11
6.3 Exclusion Criteria 11
7. STUDY PROCEDURES 11
7.1 Recruitment 11
7.2 Enrolment and Eligibility Assessment 12
7.3 Informed Consent 12
7.4 Randomization, blinding and code-breaking 12
7.5 Zio Patch administration 13
7.6 EQ-5D questionnaires 13
7.7 Discontinuation/Withdrawal of Participants from Study 13
7.8 Definition of End of Study 13
8. STATISTICS AND ANALYSIS 13
8.1 Initial assumptions 13
8.2 Sample size and predicted number of diagnoses 14
8.3 Analysis of outcome measures 15
8.5 Exploratory assessments 15
9. DATA MANAGEMENT 16
9.1 Access to Data 16
9.2 Data Recording and Record Keeping 16
10. ETHICAL AND REGULATORY CONSIDERATIONS 16
11. FINANCE AND INSURANCE 17

12. PUBLICATION POLICY 17
13. Appendix 1: Calculation of the CHA_2DS_2-VASc score 18
14. Appendix 2: Modelling of expected prevalence of AF over time and statistical power calculations . 19
* interim AF detection rate by patch 22
15. REFERENCES 23

1. SYNOPSIS

**Study Title**	AMALFI (Active Monitoring for AtriaL Fibrillation): A randomized controlled trial of screening for silent atrial fibrillation in high-risk individuals.
**Short title**	AMALFI
**Study Design**	Randomized controlled trial
**Study Participants**	Individuals with a CHA_2DS_2-VASc score ≥ 3 (men) or ≥ 4 (women), aged ≥ 65 years without known atrial fibrillation (AF) identified from primary care records.
**Planned Sample Size**	At least 2500 and up to 5000 (including up to 2500 randomized to the Zio Patch and 2500 to the control arm)
**Primary Objective**	To assess the effect of 2 weeks of continuous monitoring using Zio Patch on the proportion of participants diagnosed with AF compared to usual care at 2.5 years of follow up from randomization (i.e. at around the midpoint of a proposed 5-year screening interval).
**Secondary Objective**	To assess the effect of 2 weeks of continuous monitoring using Zio Patch on:<br>i) the proportion of participants diagnosed with AF compared to usual care at 2.5 years of follow-up in subgroups split by age (<80 and ≥80 years) and by sex;<br>ii) the mean times with AF diagnosed compared to usual care after 5 years of follow-up from randomization in the overall cohort, and in subgroups split by age (<80 and ≥80 years) and by sex.
**Protocol Number**	CTSU_AMALFI 1
**ISRCTN number**	15544176

2. ABBREVIATIONS

AF	Atrial Fibrillation
AHRE	Atrial High Rate Episodes
CHA_2DS_2-VASc	Ischaemic stroke risk prediction tool for patients with AF (see Appendix 1)
CTSU	Clinical Trial Service Unit & Epidemiological Studies Unit
ECG	Electrocardiogram
FDA	Food & Drug Administration
GCP	Good Clinical Practice
GP	General Practitioner
HRA	Health Research Authority
NHS	National Health Service
NIHR	National Institute for Health Research
SCAF	Silent/sub-clinical AF

3. BACKGROUND AND RATIONALE

3.1 AF: A global health problem associated with substantial morbidity and mortality

Atrial fibrillation (AF) is a rapidly growing public health and economic burden.[1] The global number of individuals with clinically diagnosed AF was estimated to be 33 million in 2010 and is expected to double by 2050.[1,2] Based on clinical cases, 9 million people above the age of 55 years are expected to have AF in Europe alone; this number is predicted to rise to about 18 million by 2060 due to demographic changes and the rising prevalence of risk factors for AF, such as obesity, diabetes and hypertension.[3] AF is associated with substantial morbidity and mortality (mostly secondary to an increased risk of stroke), reduced quality of life, and a considerable financial burden.[1,2,4] In the UK, the cost of caring for patients with AF and its complications in the year 2000 accounted for around 1-2% of total NHS expenditure.[5]

These figures are likely to underestimate the true prevalence of AF, as analysis of prolonged ECG monitoring in patients presenting in sinus rhythm has detected silent/sub-clinical AF (SCAF) episodes in around 6-45% of subjects, depending on the type and duration of monitoring, the characteristics of the study population (in particular, age and medical history), and the definition of AF.[6] SCAF may have a significant impact on health and disability;[7] for example, it may be linked to a significant proportion of strokes of undetermined aetiology (so-called "cryptogenic strokes" which represent approximately 25% of all ischaemic strokes).[8–10]

3.2 Detecting SCAF

A key limiting factor in the detection of SCAF is the short-lasting and infrequent nature of the arrhythmic episodes.[11,12] One opportunistic ECG recording or pulse palpation will therefore have low specificity and sensitivity for AF diagnosis. A systematic review, including 30 studies and over 122,000 participants, found that the prevalence of SCAF detected by single time-point screening (by ECG or pulse palpation) was modest (1.0%, 95% CI 0.89-1.04% in all participants and 1.4% (95% CI 1.2-1.6%) in those aged ≥65).[13] The STROKESTOP study of screening in 7000 individuals without known AF aged 75-76 detected SCAF in 0.5% of their cohort on the first ECG. However, with repeated intermittent ECGs (mean 26 per participant) over a two week period, this proportion increased to 3%.[14]

By contrast, long-term ECG monitoring using an implanted device in very high-risk older patients detected a SCAF rate (AF episodes lasting ≥ 5 minutes) of 34% per person year (95% CI, 28-43%). In these very high-risk patients, longer durations of SCAF were also common, with rates of AF ≥30 minutes, ≥6 hours and ≥24 hours being ~22%, ~7%, and ~3% per person year, respectively.[11] Similarly, in patients with a recent cryptogenic stroke (in whom AF was initially excluded by 12-lead ECG and 24 hours or longer Holter ECG recordings), long-term ECG monitoring using an implanted cardiac monitor detected AF in 8.9% of participants at 6 months (*vs.* 1.4% in those allocated to standard care).[10] The EMBRACE study found a 5-fold increase in AF detection using a 30-day event recorder compared to conventional ECG monitoring.[9]

In summary, whilst the reported prevalence of SCAF varies depending on the screening methodology, medical history, and characteristics of the participants, there appears to be a significant proportion of individuals with undetected AF across healthcare systems and for these individuals, stroke may be SCAF's first clinical manifestation.

3.3 Is stroke risk different in SCAF?

Patients with clinically diagnosed AF have a five-fold increase in the risk of ischaemic stroke. Strokes that are related to underlying AF have been associated with greater neurological deficit and an increased risk of permanent disability compared to non-embolic strokes.[9,15] Effective prevention of thromboembolic events is available and well-established. Oral anticoagulation with either a vitamin K antagonist or a direct oral anticoagulant (DOAC), when used appropriately in patients with AF and a CHA_2DS_2-VASc score ≥2 (see Appendix 1), can reduce stroke risk by up to 65%.[16] A key objective in AF management is therefore to identify patients with AF early and to assess them for oral anticoagulation.

There are limited data to allow a comparison of outcomes in patients with SCAF versus clinically diagnosed AF. A meta-analysis, which included only 2 randomized trials and 4 observational studies (involving 2500 patients with SCAF and 8000 patients with symptomatic AF) found no clear difference in outcomes (and wide confidence intervals) between those with SCAF vs those with symptomatic AF: all-cause death (RR 1.38, 95% CI 0.82-2.17); cardiovascular death (RR 0.85, 95% CI 0.53-1.36); and stroke/thromboembolism (RR 1.72, (95% CI 0.59-5.08).[17] However, the significant heterogeneity across these studies may account for the lack of difference in outcomes.

Rhythm data recorded by cardiac implanted electronic devices have also been used to assess the relative risk of stroke in patients with device-detected SCAF. In contrast to studies with documented 12-lead ECG evidence of AF, implanted devices may also detect other atrial tachyarrhythmias or be over-sensing and thus they refer to “atrial high rate episodes” (AHRE) with cut-off rates and durations varying between studies.[6] The ASSERT trial studied 2500 patients aged ≥65 years who had recently had a pacemaker or defibrillator inserted, but with no prior history of AF, and interrogated the devices for AHRE (defined as atrial episodes at a rate of >190 beat/min lasting at least 6 minutes) at 3 months after implantation.[18] After a mean follow-up of 2.5 years, those found to have subclinical AHRE in the first 3-months had a 2.5-fold increase in the risk of thromboembolic events (HR 2.49, 95%CI 1.28-4.85) compared to those without AHRE; about half of what would be expected in similar patients with persistent AF.[18] More recently, the ASSERT investigators have examined the relationship between duration of AHRE episodes and the occurrence of stroke or systemic embolism and found that patients with AHRE duration between 6 minutes and 24 hours were not significantly different from patients without SCAF. Only AHRE episodes >24 hours were associated with an increased risk of ischaemic stroke or systemic embolism.[19]

In summary, SCAF may carry a significant thromboembolic risk which appears to be dependent on the SCAF burden and the presence of established risk factors for ischaemic stroke.

3.4 Wilson & Jungner criteria for screening

Screening for AF would adhere to many of the Wilson & Jungner principles for a screening programme.[20] SCAF is a common, important and growing health problem, with a suspected prevalence between 1 and 35% depending on age, risk factors, duration of monitoring and definition of AF in terms of duration of arrhythmia. There is a range of potential approaches to screening, including non-invasive tests with a high degree of acceptability to participants. AF is an important risk factor for stroke and there is an established, highly effective preventative treatment for AF-associated thromboembolic events in the form of anticoagulation. Failure to identify and treat AF leaves patients at a considerably higher risk of stroke, disability and death.

There are precedents from other successful screening programmes. For instance, in 2003 the UK established the first national screening programme in the world for diabetic retinopathy. Retinal screening was felt to be important as early stages of the disease were usually asymptomatic and yet diabetic retinopathy was the leading cause of blindness in diabetic adults. Relying on primary care physicians or opportunistic detection of early retinopathy in clinic was proving ineffective as it was technically difficult and not given protected time in routine care. Retinal photography at that time required expensive equipment and highly skilled staff. As a result diabetic retinopathy was typically not diagnosed until late-stage, irreversible disease had developed. Nationwide systematic screening has made the retinal assessment cheap and standardised.[21] The information obtained from screening now allows the healthcare team to identify individuals who would benefit from early intervention, thereby slowing or preventing disease progression.[21,22]

3.5 Approaches to screening for SCAF

SCAF detection presents different challenges, particularly as arrhythmic episodes are short and infrequent and thus potentially difficult to capture. Screening for SCAF has previously been suggested and trialled, although a Cochrane review found only one study met their inclusion criteria.[23] This study found that both opportunistic screening with pulse palpation and systematic screening with a single ECG increased AF detection over usual care (OR 1.58, 95% CI 1.10 - 2.29; and OR 1.57, 95% CI 1.08 - 2.26, respectively).[23] Opportunistic screening appeared to be more cost-effective with an incremental cost per additional case detected of £337 compared to £1,514 for systematic screening.[24] However, opportunistic screening relies on healthcare professionals adding to their usual daily work a task which may often be overlooked. Furthermore, single time-point ECGs can considerably under-estimate the prevalence of SCAF and so a systematic approach with a prolonged period of screening may improve detection rates and reduce the number of subsequent adverse events (*e.g.* stroke).

More recently the REHEARSE-AF investigators randomized 1000 UK patients ≥65 years old with a CHADS-VASc score ≥2 to twice-weekly ambulatory ECG monitoring (30 seconds of single lead monitoring using the AliveCor Kardia monitor attached to a WiFi-enabled iPod, “iECG”) versus routine care. Over the 12-month study period there was a 4-fold increase in AF diagnosis in the iECG group compared with routine care (19 vs 5, HR 3.9, 95% CI 1.4-10.4, p=0.007) at a cost of around £8000 per AF diagnosis. The device was reported to be easy to use and acceptable to this group of patients.[25]

The Zio Patch (iRhythm Technologies, San Francisco CA) is a non-invasive single lead ECG monitoring device. Unlike a Holter monitor, the Zio Patch is discrete and water-resistant, and allows the person wearing it to participate in all routine daily activities with minimal disruption. In a pilot study among about 1600 participants of UK Biobank, acceptability was good and the median wear time was 12.7 days with a median analysable ECG time of 99.1%. iRhythm Technologies have developed and validated an automated system that processes and analyses the ECG data (the ZEUS system) using beat-by-beat QRS detection and a sophisticated analysis algorithm to detect rhythm disturbances such as AF and atrial flutter, their rate, frequency and duration and the total AF burden. The output of the automated analysis is checked by a trained operator and a detailed report is provided to the GP by mail. FDA approval has been received to use the Zio Patch and the ZEUS algorithm in clinical practice, and the system was launched in the UK in October 2014. The device has been subject to NICE evaluation.[26]

3.6 AF screening according to age

In addition to the variety of methods available for AF screening, there is considerable heterogeneity in suggested approaches with respect to the most suitable populations to be screened, in particular regarding the optimal age group in whom screening should be performed. The prevalence of clinically diagnosed AF is associated with increasing age (from 0.1% in adults <55 years, to 9% in those aged ≥80 years).[27] It is therefore reasonable to expect that subclinical atrial fibrillation is also more frequent in older populations – hence, screening yield (i.e. the proportion of new AF found in a particular group, with a particular screening intervention) may vary greatly with age.

Given that any downstream clinical benefits from screening will be determined by the amount of subclinical AF detected (versus a control group), screening older populations may yield larger differences in AF detection, initiation of anticoagulation, and reduction in embolic stroke (also as stroke risk is higher in older people). This "enrichment" based on age may dictate more favourable cost-effectiveness assessments.

However, older patients also have increased healthcare contact and are more likely to undergo cardiac monitoring for clinical purposes (other than screening), potentially increasing the likelihood of AF detection through usual care. On the other hand, elderly populations have less potential Quality-Adjusted Life years to be gained from medical interventions, may have physical or cognitive limitations that hinder their participation in screening programmes, and have increased frequency of bleeding risk factors that may prevent initiation of anticoagulation if AF were to be detected. Assessment of the impact of screening for SCAF in younger and older age groups will help inform the choice of population for any future screening programme.

3.7 AF screening according to sex

AF incidence is ~50% higher in males (potentially explained by higher cardiovascular risk factor prevalence), with women typically developing AF about 10 years later than men.[28,29] This difference could potentially mean that screening for AF would be more effective in men than women.[23] However, the lifetime risk of developing AF may be similar in both sexes (>30%), as women generally live longer. Moreover, any differences in screening uptake by sex or initiation of anticoagulation may also greatly impact the effectiveness of AF screening on downstream outcomes. For example, while men are more likely to attend colorectal screening than women,[30,31] they may be more reluctant to contact their healthcare providers and less likely to attend healthcare services.[32,33] Confirmation of screening efficacy and cost-effectiveness in both sexes is therefore important for future planning and policy decisions.

4. STUDY AIMS

The AMALFI trial aims to determine the effect of 2 weeks of continuous non-invasive ECG monitoring using the Zio Patch compared to usual care on rates and time diagnosed with AF over a follow up period of 5 years among high-risk individuals. Practice guidelines recommend oral anticoagulation based on the CHA_2DS_2-VASc score (see Appendix 1) and independently of AF burden.[34,35] By targeting the screening to those older than 65 years and with a CHA_2DS_2-VASc score ≥3 (men) or ≥4 (women) then the subsequent management of those individuals who are identified with SCAF can be directly informed by current guidelines.[35]

The primary outcome is <u>diagnosis of AF</u> (as recorded in the electronic GP record) by <u>2.5 years</u> of follow up from randomization (i.e. at around the midpoint of a proposed 5-year screening interval).

The secondary outcomes for the study include assessment of the primary outcome in subgroups split by age (<80 and ≥80 years) and by sex at 2.5 years, as well as the effect of monitoring on the times with AF diagnosed (as above) by 5 years of follow-up in the overall cohort and in the same subgroups.

Further analyses will also include cost-effectiveness assessments aimed at capturing differences in healthcare resources, jointly with differences in health-related quality of life, AF cases and future AF-related events.

5. STUDY DESIGN

AMALFI is a randomized controlled trial comparing a single 2-week screening period for AF with the Zio Patch to usual care in individuals with no history of AF and elevated risk of stroke, based on the CHA_2DS_2-VASc score (see Appendix 1). Eligible participants will be identified via an electronic search of GP practice data, approached by mail by their GP and offered the opportunity to participate. In total, at least 2500 and up to 5000 participants will be randomized to Zio Patch or usual care and will be followed-up for an average of about 25 years (Figure 1), initially via their GP records for the primary and secondary outcomes and then via NHS Digital only. A preliminary feasibility study will be conducted to identify and resolve potential practical issues, e.g. difficulties with patients self-administering devices.

Participants in the Zio Patch arm will be sent a barcoded Zio Patch to self-administer,[36] which will be returned by post directly to the UK iRhythm office for processing before the data is sent to iRhythm's laboratories in the USA for analysis. A report detailing any AF episodes and burden over the duration of ECG recording will be sent by iRhythm to the Central Coordinating Office, CTSU, who will be able to link the report to the individual participant via the patch barcode. The study team will then provide relevant information related to the presence and duration of AF to the participant's GP. The study team will not directly initiate treatment or act on the information from the Zio Patch data. Instead, a letter detailing relevant results related to the diagnosis of AF will be sent back to the participant's GP. The subsequent management will be at the discretion of the GP, allowing the doctor and participant to discuss the management strategy, including anticoagulation, independently.

Relevant outcome data from the electronic health record (including both GP-held data and centrally-held data from NHS Digital) data will be collected at about 1 year from randomization[*], at 2.5 years from randomization (for analysis of the primary outcome and respective subgroup analyses) and at 5 years from randomization (for analysis of the secondary outcome of mean times with AF and respective subgroup analyses). Data items will include documentation of AF diagnosis in the GP record, medication use (including anticoagulants) and hospital attendance and admission information. Further follow up, via NHS Digital central records, will then take place at intervals over the following 20 years.

[*] Although the primary and secondary outcomes will be based on data collected at 2.5 and 5 years respectively from randomization, a preliminary data collection will be undertaken at 1 year to ensure that full follow-up information is available for participants who move to a different GP practice or who die prior to the 2.5 year data collection.

Figure 1: Outline of trial design

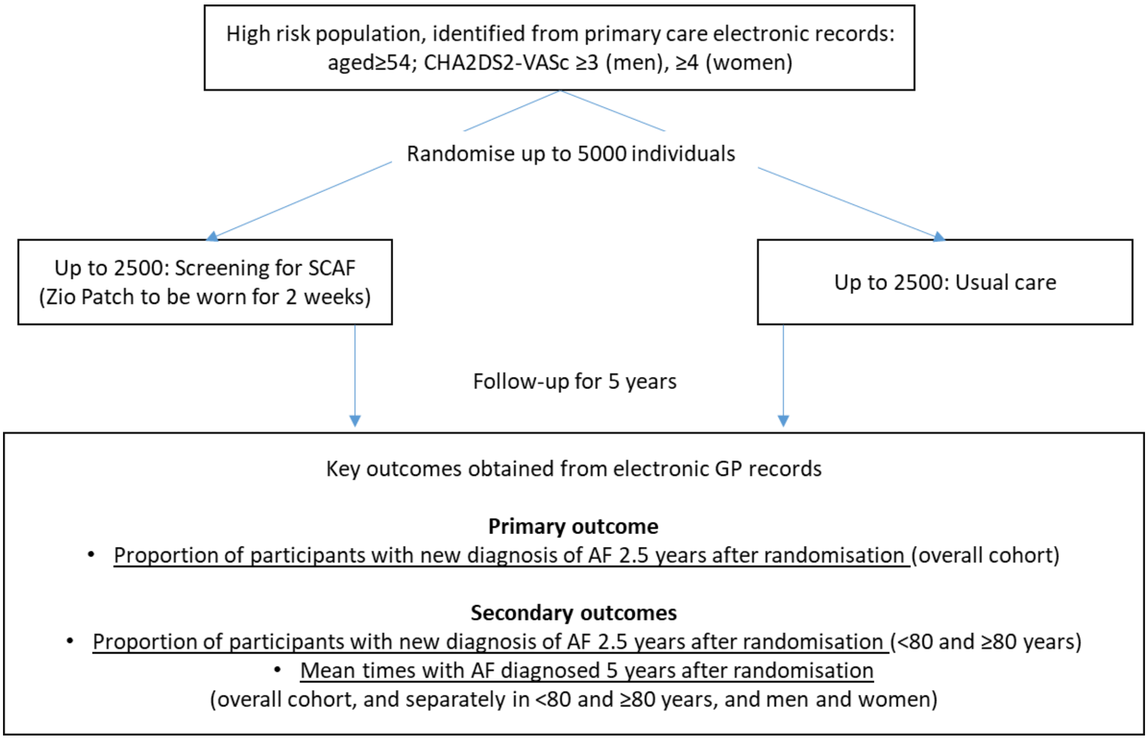

6. PARTICIPANT IDENTIFICATION

6.1 Study Participants

Participants are eligible for randomization if they satisfy all the inclusion and exclusion criteria and their GP is agreeable to their participation.

6.2 Inclusion Criteria

- Male or Female, aged ≥65 years old.
- CHA_2DS_2-VASc score ≥3 in men or ≥4 in women (see Appendix 1)
- Participant is willing and able to give informed consent for participation in the study.

6.3 Exclusion Criteria

The participant may not enter the study if ANY of the following apply:

- Known to have AF or atrial flutter
- Latex allergy (given the potential for an allergic reaction to the Zio Patch).

7. STUDY PROCEDURES

7.1 Recruitment

GP practices (identified via local Clinical Research Networks and by direct approach) will identify eligible patients by running a predetermined electronic search of the practice database using the relevant local primary care software e.g. EMIS, SystemOne. An invitation letter, including an information leaflet, consent form, and Freepost return envelope, will then be sent by post (or, if

practical, electronically via email) by the GP. Web-based and written study information will be available to provide potential participants with further information to help them make an informed decision as to whether to participate. A 24-hour Freephone service (established by CTSU for other large heart disease trials) will be available for study participants, their carers or their doctors to discuss any aspects of the study with experienced clinical staff at the coordinating centre.

7.2 Enrolment and Eligibility Assessment

There will be no face to face confirmation of eligibility. Participants will be asked to complete a baseline randomization questionnaire at the time of enrolment. This will include basic demographic information as well as medical information to confirm baseline CHA_2DS_2-VASc score (e.g. history of hypertension, stroke or diabetes, see Appendix 1).

7.3 Informed Consent

All participants will be required to provide written informed consent by returning a completed consent form to the coordinating centre. Information for participants regarding the study will be available on the study website and in written form. There will be the option for telephone or email enquiries to be made to the study team for any questions prior to enrolment. It will be clearly stated that the participant is free to withdraw from the study at any time for any reason without prejudice to future care, without affecting their legal rights, and with no obligation to give the reason for withdrawal. The participant will be allowed as much time as wished to consider the information, and the opportunity to question the study team, their GP or other independent parties to decide whether they will participate in the study. The original signed consent form will be retained at the central coordinating office and a copy will be returned to all participants who subsequently go on to be randomized into the trial.

7.4 Randomization, blinding and code-breaking

Participants who indicate on the randomization questionnaire that they are eligible and willing to join the trial, and provide written consent will be randomized by the central computer in CTSU, using a minimisation algorithm to ensure balance by important baseline variables.[37]

Eligible patients will be randomized in a 1:1 ratio to a single 2-week ECG monitoring period with the Zio Patch versus usual care. The study will not employ a sham-patch and neither the trial team nor the participants will be blinded to the intervention. All participants will receive a letter informing them which group them have been assigned to. The participant's GP will be notified that the participant has been randomized and the study arm; a copy of the participant's consent form (signature page) will also be included for reference. For all participants screened with the Zio Patch, GP's will be sent the ECG findings related to the presence and duration of AF episodes, along with any details that might be useful to interpret these results (such as the duration of monitoring). Participants will also be sent this results letter, along with a cover letter. Future management of the monitoring results will be at the GPs discretion. Additional findings will also be communicated to GPs if considered to be clinically important. A complete monitoring report (including all incidental findings) will not be routinely provided to GPs but will be available if required.

7.5 Zio Patch administration

For those participants randomized to receive the Zio Patch, the device will be posted to their home address with instructions on how to apply and care for it. Email and Freephone telephone advice lines will be available for those who need support or encounter problems.

Participants will be asked to wear the Zio Patch continuously for 2 weeks. A return envelope will be provided so that, once the two week period is completed, participants can post back the device for analysis. If participants do not return the device, efforts will be made by the research team to contact them to encourage them to return the device.

7.6 EQ-5D questionnaires

In order to inform the planned cost-effectiveness analyses, randomized participants will be asked to self-complete an EQ-5D questionnaire (either via postal or digital methods) once recruitment has been completed and at about 2.5 years afterwards.

7.7 Discontinuation/Withdrawal of Participants from Study

Each participant has the right to withdraw from the study at any time, which will be made clear during the consent process. Any withdrawals will be recorded in the study database, including, where available, the reason for doing so.

7.8 Definition of End of Study

The end of study is defined as the date of the last electronic data download (from either central registries (NHS Digital) or from a participating GP practice. For the primary comparison, data will be sought at 2.5 years after randomization. For the secondary outcome of mean times with AF, further data will be sought at 5 years after randomization. However it is intended to continue to request longer-term data from NHS Digital (for up to 25 years after randomization) in order to undertake longer-term exploratory assessments of the clinical impact of allocation to screening (see Section 8.4).

8. STATISTICS AND ANALYSIS

8.1 Initial assumptions

Study duration: Logically, the length of the study should be the proposed time between screenings if this were put into practice. This will be achieved through the assessment of the secondary outcome of mean times with AF diagnosed at 5 years. However, a follow-up period of 25 years is proposed in AMALFI to allow for further research.

AF detection with usual care (opportunistic screening): The rate of opportunistic detection of AF in this population is unclear. Data from a 2014/2015 screening initiative across Dorset Clinical Commissioning Group reported opportunistic detection of SCAF in 0.7% of 61,000 people over the age of 65 who had a one-off pulse check while attending a flu clinic.[38] However the proportion of attendees receiving a pulse check varied considerably between practices, ranging from 8% to 78% of individuals, with an average of 51%. So, among <u>all</u> those aged ≥65 years old SCAF may only be detected opportunistically in about 0.3-0.4% per annum.

The AMALFI population (with elevated CHA_2DS_2-VASc score) will tend to be at somewhat higher risk of SCAF, and so we anticipate that usual care with opportunistic screening by the GP may identify AF in about 0.7% per annum.

AF detection with screening: The incidence of SCAF detection by screening with Zio Patch is unknown in this high risk population. Among 7000 people aged 75 screened with intermittent ECGs over a 2-week period, the rate of detection of SCAF was about 3%.[14] However, in AMALFI the monitoring will be continuous for two weeks (likely to provide higher rates of detection). Taking into account a moderate level of non-compliance with the patch, we estimate that a two-week screening with Zio Patch may lead to a diagnosis of AF in around 3.75% participants. Details of the assumptions and modelling of the rates for the purposes of the statistical power calculations can be found in appendix 2.

AF detection after the screening period: In the screening group it is anticipated that further cases will continue to be identified by usual care after screening. These rates are assumed to increase with time from screening to converge with the rate found by usual care (0.7% per annum) and range from 0.2% during the first year following randomization, to 0.5% during the fifth year (see Appendix 2).

8.2 Sample size and predicted number of diagnoses

Given the assumptions above, the expected prevalence of AF over time in the AMALFI trial is shown in Figure 2 (see Appendix 2 for statistical model and calculations).

Figure 2: Estimated cumulative percent of participants diagnosed with AF

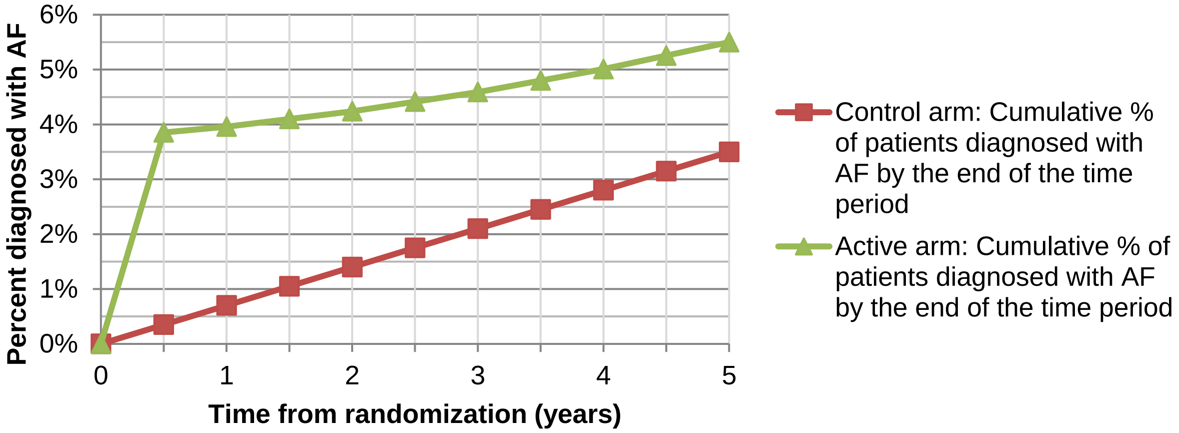

Based on these assumptions, a sample size of 1250 per arm (2500 participants) would give more than 90% power to detect the estimated difference in proportion with a diagnosis of AF at 2.5 years of 1.75% versus 4.4% (ratio of about 2.5) at 2p<0.05 (see Appendix 2). Therefore, the initial overall sample size was set at 2500 participants.

As recruitment progressed, it became apparent that the trial would be able to achieve its initial target sample size without exhausting the available pool of interested GP practices, offering the possibility of extending recruitment in a cost-effective way. Based on over 1250 participants randomized before 1st February 2021 and their observed adherence to wearing the patch and AF detection rates by patch, a larger sample size of up to 5000 participants provides increased reassurance regarding a lower than anticipated AF rate ratio (e.g. around 3000 participants would

be required to maintain >90% at 2p<0.05 if the ratio were to be 2 instead of 2.5 as predicted, with 4100 participants required to maintain >90% power at 2p<0.01). Furthermore an increase in sample size allows for reliable assessment of the effects of screening for SCAF in the proposed subgroups (by age and by sex).

8.3 Analysis of outcome measures

The fundamental assessments of efficacy will involve an "intention-to-treat" analysis among all randomized patients of allocation to screening versus allocation to usual care, irrespective of compliance.

Analysis of the primary outcome:

- The chi-square test will be used to compare the proportion of participants diagnosed with AF at 2.5 years of follow up in all those randomized to screening versus all those randomized to usual care.

Analysis of the secondary outcomes:

- The chi-square test will be used to compare the proportion of participants diagnosed with AF at 2.5 years of follow up in those randomized to screening versus those randomized to usual care separately by age subgroups of <80 and ≥80 years and by sex subgroups at randomization, with a test for heterogeneity of the results between the two subgroups.

- Mean durations of time with AF diagnosed after 5 years of follow-up from randomization in all those randomized to screening compared to all those randomized to usual care will be compared using a permutation test to assess statistical significance (in the overall cohort and, separately, by age subgroups of <80 and ≥80 years and by sex subgroups at randomization, with a test for heterogeneity between the two subgroups). Those with no diagnosis of AF by the end of the study will contribute zero time with AF.

8.4 Cost-effectiveness analyses

In addition to investigating the impact of screening on AF detection rates, the results from AMALFI will also inform a cost-effectiveness analysis aimed at capturing differences in healthcare resources (e.g. GP visits, medications, hospital visits), as well as differences in health-related quality of life (based on the standard EQ-5D questionnaire), AF cases, and AF-related events. Details of these analyses will be described in a separate analysis plan.

8.5 Exploratory assessments

8.5.1 Subgroup analysis based on an age cut-off of 75 years

Assessment of the effects of screening for SCAF in those ≥75 years old is of interest from a clinical perspective. This age threshold is included in the calculation of the CHA_2DS_2-VASc score and all individuals ≥75 years old would warrant consideration for initiation of anticoagulation if AF were to be detected. However, based on the initial randomization of 1250 participants and their observed adherence to wearing the patch, age distributions and AF detection rates by patch, even a trial of 5000 participants would have limited statistical power in the subgroup aged <75 years

at randomization. Therefore, the confirmation of the study results in these two subgroups will be performed as an exploratory assessment only.

8.5.2 Rates of anticoagulation and hospitalization between screen-detected and clinically-detected AF (non-randomized assessment)

With only about 500 cases of AF expected during the 5-year period of the trial, a randomized comparison of the effects of screening for SCAF on the prevention of AF-related morbidity and mortality would have very low statistical power. However, in order to explore the potential clinical impact of screening we will perform an exploratory retrospective non-randomized comparison of rates of anticoagulation and of hospitalization (inpatient and outpatient) among those who were diagnosed with AF/SCAF by the end of follow-up according to whether or not they were diagnosed by Zio Patch screening or by usual care. It is recognised that such non-randomized comparisons have considerable limitations. However it is hypothesised that the mode of diagnosis of AF may influence future management decisions (for example the decision whether to anticoagulate or not, or to refer to secondary care or not). These non-randomized comparisons may be informative in exploring such potential clinical management biases.

8.5.3 Long-term assessments

After the end of the scheduled study period, information about hospitalizations, medications, and other clinical events will continue to be collected from NHS Digital on all surviving participants (unless they have withdrawn consent) for up to an additional 20 years. These data will be used for longer-term exploratory assessments of the clinical impact of screening.

9. DATA MANAGEMENT

9.1 Access to Data

Direct access will be granted to authorised representatives from the Sponsor and host institution for monitoring and/or audit of the study to ensure compliance with regulations.

9.2 Data Recording and Record Keeping

Personal data will be entered electronically on a CTSU central restricted database that is password protected and following an operating procedure for data handling. Access to the data is granted on a ‘need-to-know’ basis, authorised by the study managers. All personal identifiers will be strongly encrypted and each participant allocated a study number at the point of enrolment. It will be possible to check back against the initial study number allocation to identify individual participants when necessary in the case of safety reasons or when needing to contact practices with results or request follow-up data.

All data will be held securely on computer servers managed by the University of Oxford, analysed by appropriately qualified staff with access restricted as appropriate for their role in the study, and used only for the medical research purposes outlined in this protocol. The data will be held for at least 25 years after the completion of the study and only used for the AMALFI study.

10. ETHICAL AND REGULATORY CONSIDERATIONS

The Chief Investigator will ensure that this study is conducted in accordance with the principles of the Declaration of Helsinki, and of Good Clinical Practice and in accordance with relevant

regulations. The protocol, informed consent form, participant information sheet and any proposed advertising material (including all relevant substantial amendments to the original approved documents) will be submitted to the Health Research Authority (HRA) for written approval. The Chief Investigator shall submit once a year throughout the study, or on request, an Annual Progress report to the REC Committee, HRA (where required) host organisation and Sponsor. In addition, an End of Study notification and final report will be submitted to the same parties.

11. FINANCE AND INSURANCE

The study is funded by the National Institute for Health Research (NIHR) Oxford Biomedical Research Centre (BRC). iRhythm is providing additional support by providing the Zio Patches and reporting service free of charge during the AMALFI study. The University has a specialist insurance policy in place which would operate in the event of any participant suffering harm as a result of their involvement in the research (Newline Underwriting Management Ltd, at Lloyd's of London).

12. PUBLICATION POLICY

The Investigators (see page 1) will be involved in reviewing drafts of the manuscripts, abstracts, press releases and any other publications arising from the study. Authors will acknowledge that the study was funded by the National Institute for Health Research (NIHR) Oxford Biomedical Research Centre (BRC). Authorship will be determined in accordance with the ICMJE guidelines and other contributors will be acknowledged.

13. Appendix 1: Calculation of the CHA_2DS_2-VASc score

	Condition	Points
C	Congestive heart failure (or Left ventricular systolic dysfunction)	1
H	Hypertension: blood pressure consistently above 140/90 mmHg (or treated hypertension on medication)	1
A_2	Age ≥75 years	2
D	Diabetes Mellitus	1
S_2	Prior Stroke or TIA or thromboembolism	2
V	Vascular disease (e.g. peripheral artery disease, myocardial infarction, aortic plaque)	1
A	Age 65–74 years	1
Sc	Sex category (i.e. female sex)	1

14. Appendix 2: Modelling of expected prevalence of AF over time and statistical power calculations

Primary outcome considerations:

Logically, the length of study should be the proposed time between screenings if put into practice (i.e. potentially 5 years). The ideal primary outcome would be the **time with AF diagnosed within 5 years of randomization**, with those not getting AF having a time of zero with AF. The rationale is that this represents the window of opportunity for intervention. However, a more pragmatic alternative outcome, which allows for a more timely assessment of the value of screening with the Zio Patch, would be the proportion with AF around the middle of the study. The areas under the curves shown in Figure 2 (see protocol Section 8) give the expected mean times spent with AF and are mostly driven by the proportions with AF in the middle of the trial. Hence the proportion of participants with AF at 2.5 years has been chosen as the primary outcome for AMALFI.

Assumptions for Figure 2 (see protocol Section 8):

The probability of being diagnosed with AF is a time dependent Poisson process with a parameter slightly increasing with time, a+bt. Additionally, we assume that if someone would have had AF diagnosed at time t, there is a decreasing probability e+ft that it would be pre-empted by patch related early detection of AF. We then chose a and b so that the AF diagnosis rates during years 1 and 2 were both 0.7% per annum, the anticipated detection rate with usual care.

e and f were chosen to allow for non-compliance and to give decreasing probabilities over time of AFs that would have been diagnosed during a time period being pre-emptively detected by patch (70% in the first year, 60% in the second year, 50% in the third, 40% in the fourth and 30% in the fifth year). An additional probability of 2% was assumed for patch related early detection of AF when no AF would have been detected within 5 years. It is assumed that patch related early detection of AF occurs evenly over the first 6 months from randomization. Table 1 below gives the estimated cumulative % of participants being diagnosed with AF at the end of each year, based on this model and the assumptions described in Section 8 of the protocol.

Sample size calculation:

Table 2 below gives a set of power calculations based on the expected proportions diagnosed with AF at half yearly points up to 5 years. It is assumed that participants are randomized 1:1 to give to equal numbers in the control and active arms. The 2.5 year row is highlighted as being a suitable mid-study comparison time point for a proposed screening interval of 5 years.

Table 3 provides a summary of power calculations for different samples sizes made possible by an extension in recruitment of up to 5000 participants for the whole cohort and under different assumptions regarding AF detection ratios. Table 4 provides the same calculations for different age and sex subgroups.

Table 1 – initial projections for proportions of new AF cases found in the active and control groups at different time points

	Placebo arm		Active arm				
Year end	% of patients diagnosed with AF within the year	Cumulative % of patients diagnosed with AF by end of year	% of patients who would have been found to have AF in year i, but found up front by Zio Patch	% of patients diagnosed by Zio Patch	Additional % of patients found to have AF within the year, but not with Zio Patch	% of patients diagnosed with AF within the year (including 2% extra found by Zio Patch, who wouldn't have been diagnosed with AF during years 1-5)	Cumulative % of patients diagnosed with AF by end of year
1	0.70%	0.70%	70%	0.49%	0.21%	3.96%	3.96%
2	0.70%	1.40%	60%	0.42%	0.28%	0.28%	4.24%
3	0.70%	2.10%	50%	0.35%	0.35%	0.35%	4.59%
4	0.70%	2.80%	40%	0.28%	0.42%	0.42%	5.01%
5	0.70%	3.50%	30%	0.21%	0.49%	0.49%	5.50%

Table 2 – sample size calculations based on the initial assumptions

Comparison time point	Expected proportion with AF at time point		Alpha	Power	Total sample size (continuity corrected)
	Control arm	Active arm			
1	0.70%	3.96%	0.05	90%	1019
1.5	1.05%	4.10%	0.05	90%	1261
2	1.40%	4.24%	0.05	90%	1565
2.5	1.75%	4.41%	0.05	90%	1914
3	2.10%	4.59%	0.05	90%	2348
3.5	2.45%	4.80%	0.05	90%	2825
4	2.80%	5.01%	0.05	90%	3406
4.5	3.15%	5.25%	0.05	90%	4004
5	3.50%	5.50%	0.05	90%	4711

Table 3 – updated power and sample size calculations under different assumptions regarding AF rates detected by patch and detection ratio between active and control groups

Assumptions						Resulting power and sample sizes						
**Group**	**Percent of population**	**AF rate detected through patch in active arm**	**Total AF rate in active arm at 2.5 years (patch rate x 1.177)**	**Ratio of AF rates in active vs control arms**	**Implied AF rate in controls (based on detection ratio)**	**n total needed at 90% power, p<0.05**	**n total needed at 90% power, p<0.01**	**Power for total of 2500, 2p<0.05**	**Power for total of 4000, 2p<0.05**	**Power for total of 4000, 2p<0.01**	**Power for total of 5000, 2p<0.05**	**Power for total of 5000, 2p<0.01**
Whole trial (original assumptions)	100%	3.75%	4.41%	2.52	1.75%	1914	2649	96%	100%	99%	100%	99%
Whole trial	100%	3.75%	4.41%	**2.5**	1.77%	1941	2687	96%	100%	99%	100%	100%
Whole trial	100%	**4.09%***	4.82%	**2.5**	1.93%	1773	2454	97%	100%	99%	100%	100%
Whole trial	100%	3.75%	4.41%	**2**	2.21%	2939	4088	84%	97%	89%	99%	96%
Whole trial	100%	**4.09%***	4.82%	**2**	2.41%	2684	3732	88%	98%	92%	99%	97%

* interim AF detection rate by patch

Table 4 – power and sample size calculations for subgroup analyses by age and sex

Assumptions						Resulting power and sample sizes					
**Group**	**Percent of population**	**AF rate detected through patch in active arm***	**Total AF rate in active arm at 2.5 years (patch rate x 1.177)**	**Ratio of AF rates in active vs control arms**	**Implied AF rate in controls (based on detection ratio)**	**n needed in subgroup for 90% power, p<0.05**	**n needed in subgroup for 90% power, p<0.01**	**Power for total of 4000, 2p<0.05**	**Power for total of 4000, 2p<0.01**	**Power for total of 5000, 2p<0.05**	**Power for total of 5000, 2p<0.01**
**Age <80**	64%	2.78%	3.27%	**2.5**	1.31%	2642	3658	89%	73%	95%	85%
**Age ≥80**	36%	6.28%	7.39%	**2.5**	2.95%	1135	1571	96%	87%	99%	95%
**Age <75**	25%	2.52%	2.96%	**2.5**	1.18%	2924	4048	41%	20%	52%	34%
**Age ≥75**	75%	4.62%	5.44%	**2.5**	2.18%	1563	2164	100%	98%	100%	100%
**Males**	54%	4.94%	5.82%	**2.5**	2.33%	1458	2018	98%	92%	99%	98%
**Female**	46%	3.09%	3.64%	**2.5**	1.46%	2367	3276	80%	60%	89%	75%

*** interim AF detection rate by patch**

15. REFERENCES

1. Chugh SS, Havmoeller R, Narayanan K, et al. Worldwide Epidemiology of Atrial Fibrillation: A Global Burden of Disease 2010 Study. *Circulation*. 2014;129(8):837-847. doi:10.1161/CIRCULATIONAHA.113.005119

2. Miyasaka Y, Barnes ME, Gersh BJ, et al. Secular Trends in Incidence of Atrial Fibrillation in Olmsted County, Minnesota, 1980 to 2000, and Implications on the Projections for Future Prevalence. *Circulation*. 2006;114(2):119-125. doi:10.1161/CIRCULATIONAHA.105.595140

3. Krijthe BP, Kunst A, Benjamin EJ, et al. Projections on the number of individuals with atrial fibrillation in the European Union, from 2000 to 2060. *Eur Heart J*. 2013;34(35):2746-2751. doi:10.1093/eurheartj/eht280

4. Ball J, Carrington MJ, McMurray JJV, Stewart S. Atrial fibrillation: Profile and burden of an evolving epidemic in the 21st century. *Int J Cardiol*. 2013;167(5):1807-1824. doi:10.1016/j.ijcard.2012.12.093

5. Stewart S, Murphy N, Walker A, McGuire A, McMurray JJV. Cost of an emerging epidemic: an economic analysis of atrial fibrillation in the UK. *Heart*. 2004;90(3):286-292. doi:10.1136/hrt.2002.008748

6. Freedman B, Camm J, Calkins H, et al. Screening for Atrial Fibrillation: A Report of the AF-SCREEN International Collaboration. *Circulation*. 2017;135(19):1851-1867. doi:10.1161/CIRCULATIONAHA.116.026693

7. Glotzer TV, Ziegler PD. Cryptogenic stroke: Is silent atrial fibrillation the culprit? *Heart Rhythm*. 2015;12(1):234-241. doi:10.1016/j.hrthm.2014.09.058

8. Sacco RL, Ellenberg JH, Mohr JP, et al. Infarcts of undetermined cause: The NINCDS stroke data bank. *Ann Neurol*. 1989;25(4):382-390. doi:10.1002/ana.410250410

9. Gladstone DJ, Spring M, Dorian P, et al. Atrial Fibrillation in Patients with Cryptogenic Stroke. *N Engl J Med*. 2014;370(26):2467-2477. doi:10.1056/NEJMoa1311376

10. Sanna T, Diener H-C, Passman RS, et al. Cryptogenic Stroke and Underlying Atrial Fibrillation. *N Engl J Med*. 2014;370(26):2478-2486. doi:10.1056/NEJMoa1313600

11. Healey JS, Alings M, Ha A, et al. Subclinical Atrial Fibrillation in Older Patients. *Circulation*. 2017;136(14):1276-1283. doi:10.1161/CIRCULATIONAHA.117.028845

12. Brambatti M, Connolly SJ, Gold MR, et al. Temporal Relationship Between Subclinical Atrial Fibrillation and Embolic Events. *Circulation*. 2014;129(21):2094-2099. doi:10.1161/CIRCULATIONAHA.113.007825

13. Lowres N, Neubeck L, Redfern J, Freedman SB. Screening to identify unknown atrial fibrillation. A systematic review. *Thromb Haemost*. 2013;110(2):213-222. doi:10.1160/TH13-02-0165

14. Svennberg E, Engdahl J, Al-Khalili F, Friberg L, Frykman V, Rosenqvist M. Mass Screening for Untreated Atrial Fibrillation: The STROKESTOP Study. *Circulation*. 2015;131(25):2176-2184. doi:10.1161/CIRCULATIONAHA.114.014343

15. Lamassa M, Di Carlo A, Pracucci G, et al. Characteristics, Outcome, and Care of Stroke Associated With Atrial Fibrillation in Europe: Data From a Multicenter Multinational Hospital–Based Registry (The European Community Stroke Project). *Stroke*. 2001;32(2):392-398. doi:10.1161/01.STR.32.2.392

16. Hart RG, Sherman DG, Easton JD, Cairns JA. Prevention of stroke in patients with nonvalvular atrial fibrillation. *Neurology*. 1998;51(3):674-681. doi:10.1212/WNL.51.3.674

17. Xiong Q, Proietti M, Senoo K, Lip GYH. Asymptomatic versus symptomatic atrial fibrillation: A systematic review of age/gender differences and cardiovascular outcomes. *Int J Cardiol*. 2015;191:172-177. doi:10.1016/j.ijcard.2015.05.011

18. H. Hohnloser S, Capucci A, Fain E, et al. ASymptomatic atrial fibrillation and Stroke Evaluation in pacemaker patients and the atrial fibrillation Reduction atrial pacing Trial (ASSERT). *Am Heart J*. 2006;152(3):442-447. doi:10.1016/j.ahj.2006.02.016

19. Van Gelder IC, Healey JS, Crijns HJGM, et al. Duration of device-detected subclinical atrial fibrillation and occurrence of stroke in ASSERT. *Eur Heart J*. 2017;38(17):1339-1344. doi:10.1093/eurheartj/ehx042

20. Wilson JMG, Jungner G, Organization WH. Principles and practice of screening for disease. Published online 1968. Accessed March 4, 2019. https://apps.who.int/iris/handle/10665/37650

21. Arun CS, Al-Bermani A, Stannard K, Taylor R. Long-term impact of retinal screening on significant diabetes-related visual impairment in the working age population. *Diabet Med*. 2009;26(5):489-492. doi:10.1111/j.1464-5491.2009.02718.x

22. Misra A, Bachmann MO, Greenwood RH, et al. Trends in yield and effects of screening intervals during 17 years of a large UK community-based diabetic retinopathy screening programme. *Diabet Med*. 2009;26(10):1040-1047. doi:10.1111/j.1464-5491.2009.02820.x

23. Moran PS, Teljeur C, Ryan M, Smith SM. Systematic screening for the detection of atrial fibrillation. *Cochrane Database Syst Rev*. 2016;(6):CD009586. doi:10.1002/14651858.CD009586.pub3

24. Fitzmaurice DA, Hobbs FD, Jowett S, et al. Screening versus routine practice in detection of atrial fibrillation in patients aged 65 or over: cluster randomised controlled trial. *BMJ*. 2007;335(7616):383. doi:10.1136/bmj.39280.660567.55

25. Halcox JPJ, Wareham K, Cardew A, et al. Assessment of Remote Heart Rhythm Sampling Using the AliveCor Heart Monitor to Screen for Atrial Fibrillation: The REHEARSE-AF Study. *Circulation*. 2017;136(19):1784-1794. doi:10.1161/CIRCULATIONAHA.117.030583

26. *NICE. Zio Service for Detecting Cardiac Arrhythmias. Medtech Innovation Briefing [MIB101] Published Date: March 2017. (Ed. Excellence, N.I.f.H.a.C.) (Https://Www.Nice.Org.Uk/Advice/Mib101, 2017).*

27. Go AS, Hylek EM, Phillips KA, et al. Prevalence of Diagnosed Atrial Fibrillation in Adults: National Implications for Rhythm Management and Stroke Prevention: the AnTicoagulation and Risk Factors In Atrial Fibrillation (ATRIA) Study. *JAMA*. 2001;285(18):2370-2375. doi:10.1001/jama.285.18.2370

28. Magnussen C, Niiranen TJ, Ojeda FM, et al. Sex Differences and Similarities in Atrial Fibrillation Epidemiology, Risk Factors, and Mortality in Community Cohorts: Results From the BiomarCaRE Consortium (Biomarker for Cardiovascular Risk Assessment in Europe). *Circulation*. 2017;136(17):1588-1597. doi:10.1161/CIRCULATIONAHA.117.028981

29. Benjamin EJ, Levy D, Vaziri SM, D'Agostino RB, Belanger AJ, Wolf PA. Independent risk factors for atrial fibrillation in a population-based cohort. The Framingham Heart Study. *JAMA*. 1994;271(11):840-844.

30. Friedemann-Sánchez G, Griffin JM, Partin MR. Gender differences in colorectal cancer screening barriers and information needs. *Health Expect Int J Public Particip Health Care Health Policy*. 2007;10(2):148-160. doi:10.1111/j.1369-7625.2006.00430.x

31. Wardle J, Miles A, Atkin W. Gender differences in utilization of colorectal cancer screening. *J Med Screen*. 2005;12(1):20-27. doi:10.1258/0969141053279158

32. Peate I. Men's attitudes towards health and the implications for nursing care. *Br J Nurs Mark Allen Publ*. 2004;13(9):540-545. doi:10.12968/bjon.2004.13.9.12967

33. Centre for Disease Control. *Utilization of Ambulatory Medical Care by Women: United States, 1997-98. Series Report 13, No. 149, 2001.*; 2001. Accessed May 25, 2021. www.cdc.gov/nchs/data/series/sr_13/sr13_149.pdf

34. January CT, Wann LS, Alpert JS, et al. 2014 AHA/ACC/HRS Guideline for the Management of Patients With Atrial Fibrillation. *J Am Coll Cardiol*. 2014;64(21):e1-e76. doi:10.1016/j.jacc.2014.03.022

35. Kirchhof P, Benussi S, Kotecha D, et al. 2016 ESC Guidelines for the management of atrial fibrillation developed in collaboration with EACTS. *Eur Heart J*. 2016;37(38):2893-2962. doi:10.1093/eurheartj/ehw210

36. Engel JM, Chakravarthy N, Katra RP, Mazar S, Libbus I, Chavan A. Estimation of patient compliance in application of adherent mobile cardiac telemetry device. In: *2011 Annual International Conference of the IEEE Engineering in Medicine and Biology Society*. IEEE; 2011:1536-1539. doi:10.1109/IEMBS.2011.6090448

37. Pocock SJ, Simon R. Sequential treatment assignment with balancing for prognostic factors in the controlled clinical trial. *Biometrics*. 1975;31(1):103-115.

38. *Opportunistic Screening for Atrial Fibrillation for over 65 Year Olds during Flu Clinics and Anticoagulation for the Prevention of Stroke in Primary Care. (Ed. Group, D.C.C.)*. (http://www.londonscn.nhs.uk/wp-content/uploads/2017/07/dorset-ccg.pdf, 2015).
