# Supplementary material for: Active Monitoring for AtriaL FIbrillation (AMALFI): Rationale, protocol, and pilot for a pragmatic, randomized, controlled trial of remote screening for asymptomatic atrial fibrillation
Source: Am Heart J. Author manuscript; Available in PMC 2026 Mar 10. (PMC7618845; doi:10.1016/j.ahj.2025.07.004)

Zio XT

Zio XT Final Report for
Sample Patient

iRhythm Technologies
www.zioreports.com

iRhythm

Date of Birth	Patient ID	Gender	Primary Indication
		Male	

Prescribing Clinician	Managing Location

Enrollment Period
14 days 0 hours
16/02/22, 12:50 to
02/03/22, 12:50

Analysis Time
13 days 23 hours
(after artifact removed)

Atrial Fibrillation/Flutter

▼ Fastest AF/AFL (HR Range 65-185 bpm, Avg 95 bpm)

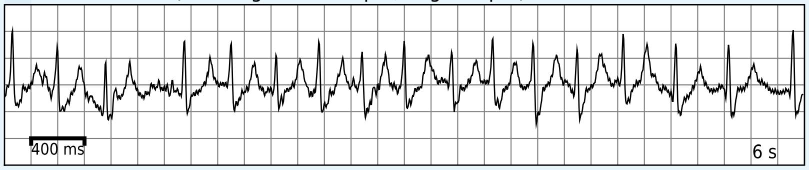

AF/AFL Burden
13%
Longest Duration
1 d 15 h
HR Range
62-185 bpm
Avg
95 bpm

Ventricular Tachycardia (4 beats or more)

None found

Pauses (3 secs or longer)

None found

AV Block (2nd° Mobitz II, 3rd°)

None found

Supraventricular Tachycardia (4 beats or more)

None found

Heart Rate

**Overall**	Max	**185 bpm**	09:27, 19/02
	Min	**53 bpm**	06:31, 22/02
	Avg	**85 bpm**	
**Sinus**	Max	**123 bpm**	08:19, 28/02
	Min	**53 bpm**	06:31, 22/02
	Avg	**84 bpm**	

Patient Events

Total Triggers: 0 **Total Diaries: 0**

Findings within ± 45 sec of triggered events or diary entries:

	Range	Trigger	Diary

None found

Ectopics

	Rare <1%	Occasional 1% to 5%	Frequent >5%

Supraventricular Ectopy (SVE/PACs)

Isolated	**Rare**	<1.0%
Couplet	**Rare**	<1.0%
Triplet	**Rare**	<1.0%

Ventricular Ectopy (VE/PVCs)

Isolated	**Rare**	<1.0%
Couplet	**Rare**	<1.0%
Triplet	**0**	

Longest Ventricular Bigeminy Episode	0 s
Longest Ventricular Trigeminy Episode	0 s

Preliminary Findings

Patient had a min HR of 53 bpm, max HR of 185 bpm, and avg HR of 85 bpm. Predominant underlying rhythm was Sinus Rhythm. Atrial Fibrillation/Flutter occurred (13% burden), ranging from 62-185 bpm (avg of 95 bpm), the longest lasting 1 day 15 hours with an avg rate of 95 bpm. Isolated SVEs were rare (<1.0%), SVE Couplets were rare (<1.0%), and SVE Triplets were rare (<1.0%). Isolated VEs were rare (<1.0%), VE Couplets were rare (<1.0%), and no VE Triplets were present.

Final Interpretation

SIGNATURE

© 2022 iRhythm Technologies, Inc. CE 0050

Events

Sinus rates > 100 bpm 6%

Sinus rates < 50 bpm 0%

Days 1 & 2

HR per 20 min period:

Max/Min — Avg

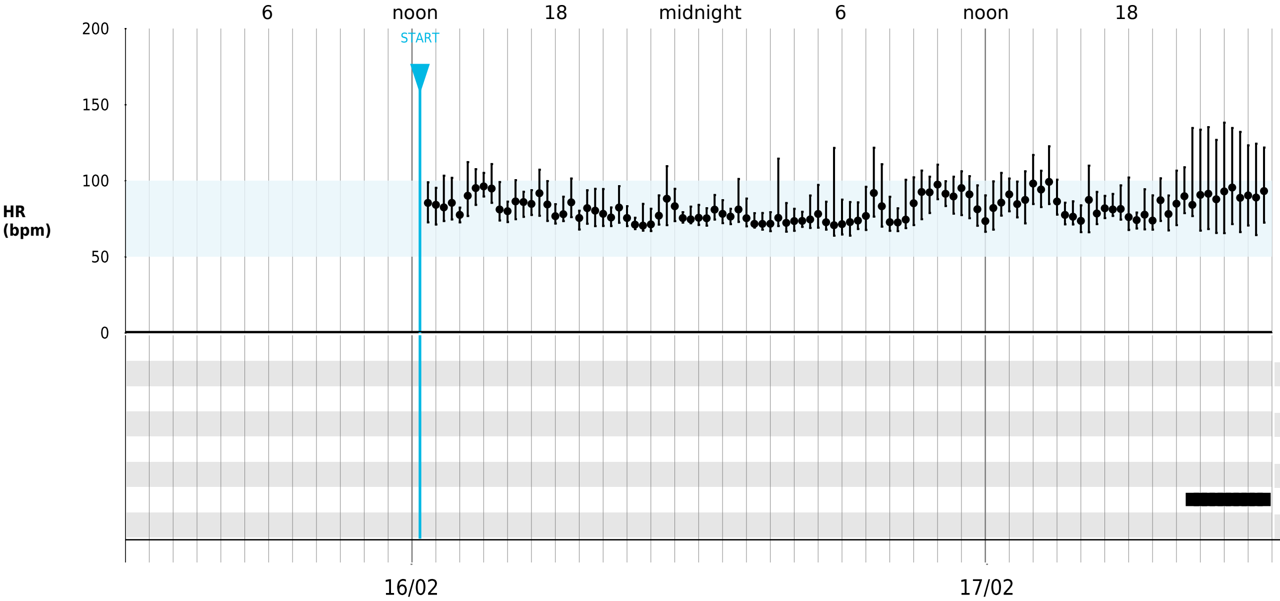

0 Patient diary events
0 Patient triggered
0 PVT, VF, TdP
0 VT
0 SVT
0 Pauses
< 1 % AF/AFL burden
0 AV Block
▼ Unanalyzable ECG

Days 3 & 4

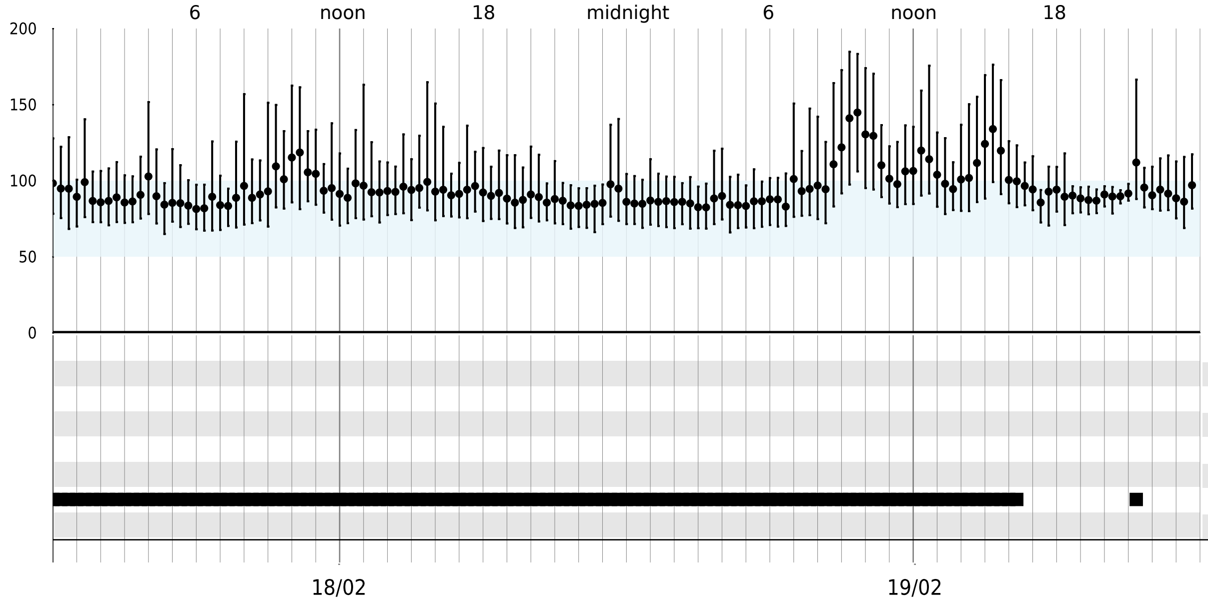

0 Patient diary events
0 Patient triggered
0 PVT, VF, TdP
0 VT
0 SVT
0 Pauses
83 % AF/AFL burden
0 AV Block
▼ Unanalyzable ECG

Days 5 & 6

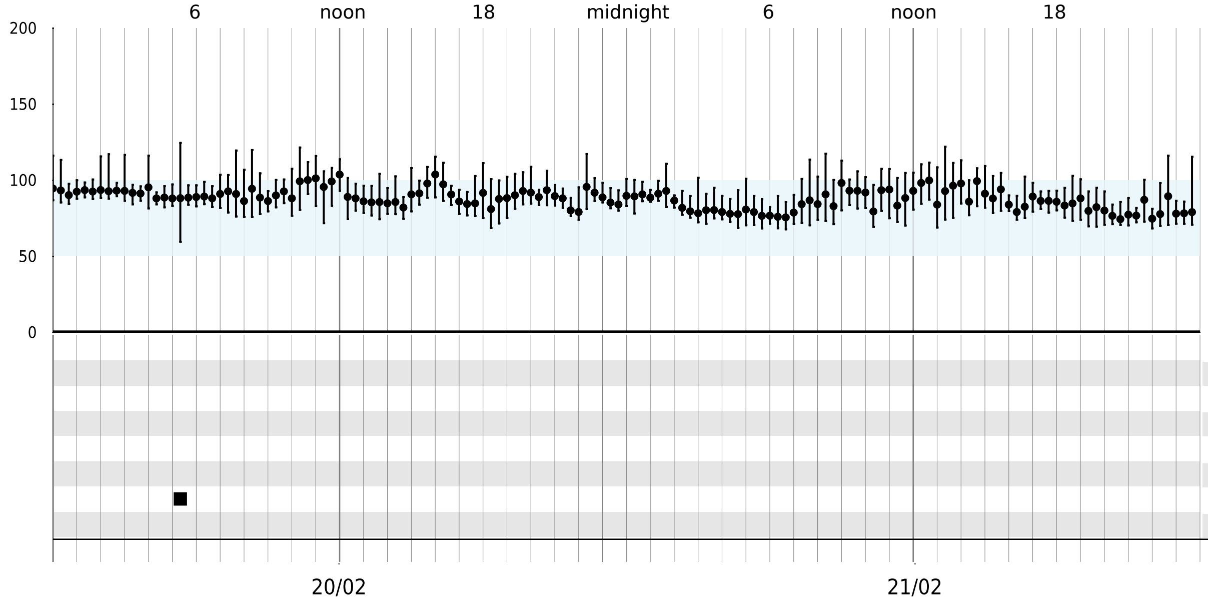

0 Patient diary events
0 Patient triggered
0 PVT, VF, TdP
0 VT
0 SVT
0 Pauses
< 1 % AF/AFL burden
0 AV Block
▼ Unanalyzable ECG

© 2022 iRhythm Technologies, Inc.

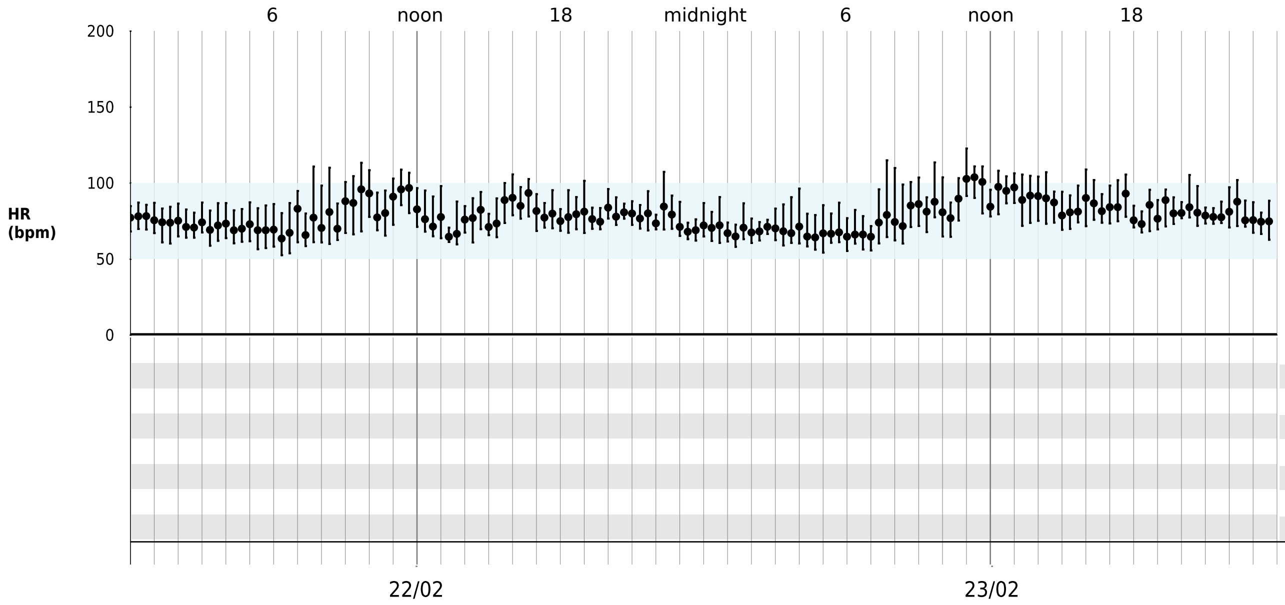

Days 7 & 8

HR per 20 min period:

Max/Min --- Avg

0 Patient diary events
0 Patient triggered
0 PVT, VF, TdP
0 VT
0 SVT
0 Pauses
0 % AF/AFL burden
0 AV Block
▼ Unanalyzable ECG

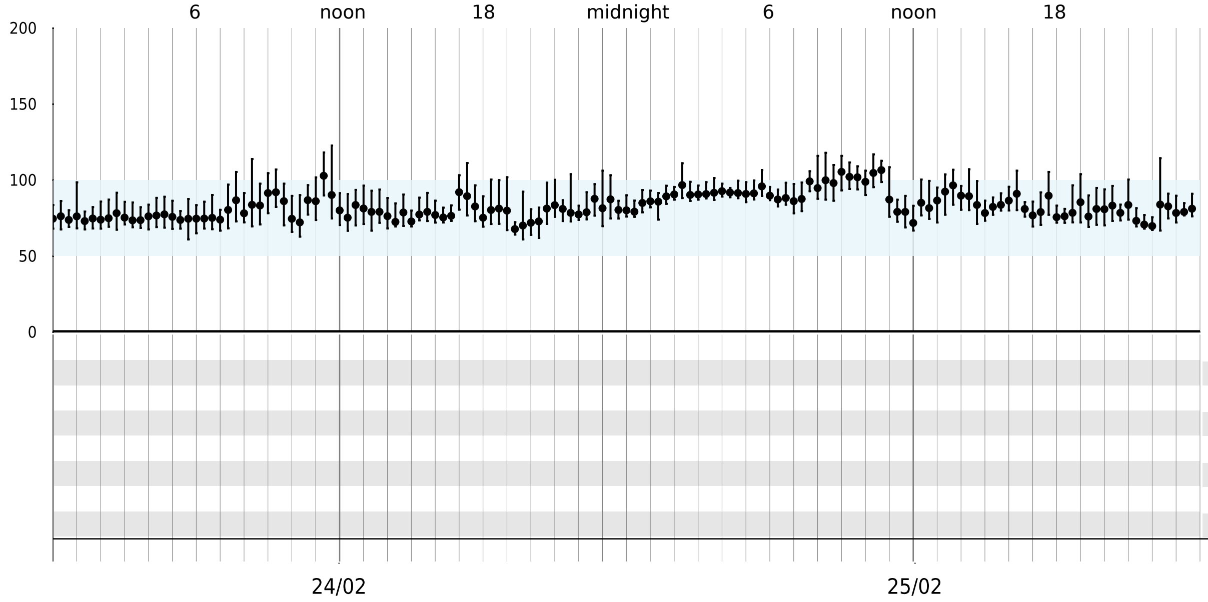

Days 9 & 10

0 Patient diary events
0 Patient triggered
0 PVT, VF, TdP
0 VT
0 SVT
0 Pauses
0 % AF/AFL burden
0 AV Block
▼ Unanalyzable ECG

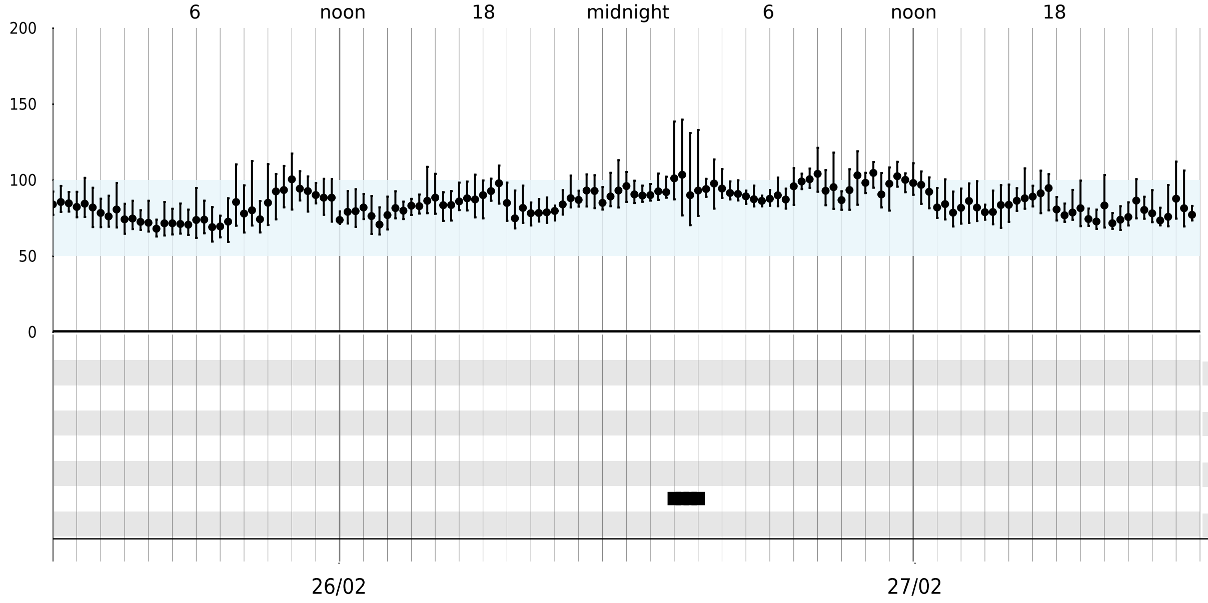

Days 11 & 12

0 Patient diary events
0 Patient triggered
0 PVT, VF, TdP
0 VT
0 SVT
0 Pauses
< 1 % AF/AFL burden
0 AV Block
▼ Unanalyzable ECG

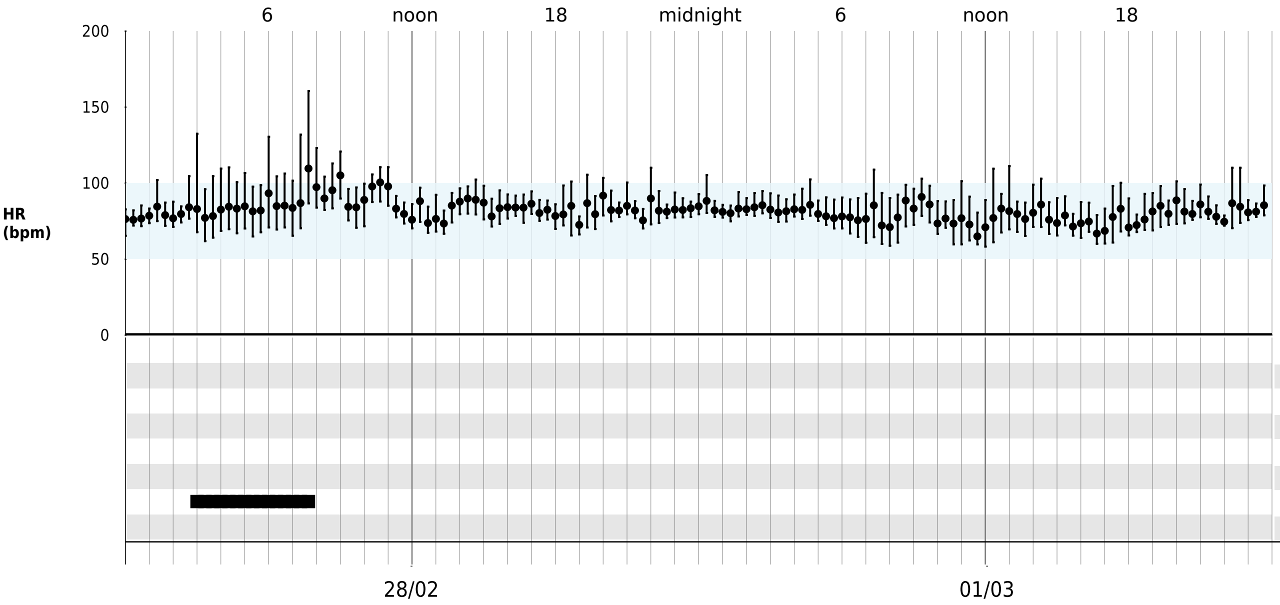

Days 13 & 14

HR per 20 min period:

Max/Min Avg

0 Patient diary events
0 Patient triggered
0 PVT, VF, TdP
0 VT
0 SVT
0 Pauses
10 % AF/AFL burden
0 AV Block
▼ Unanalyzable ECG

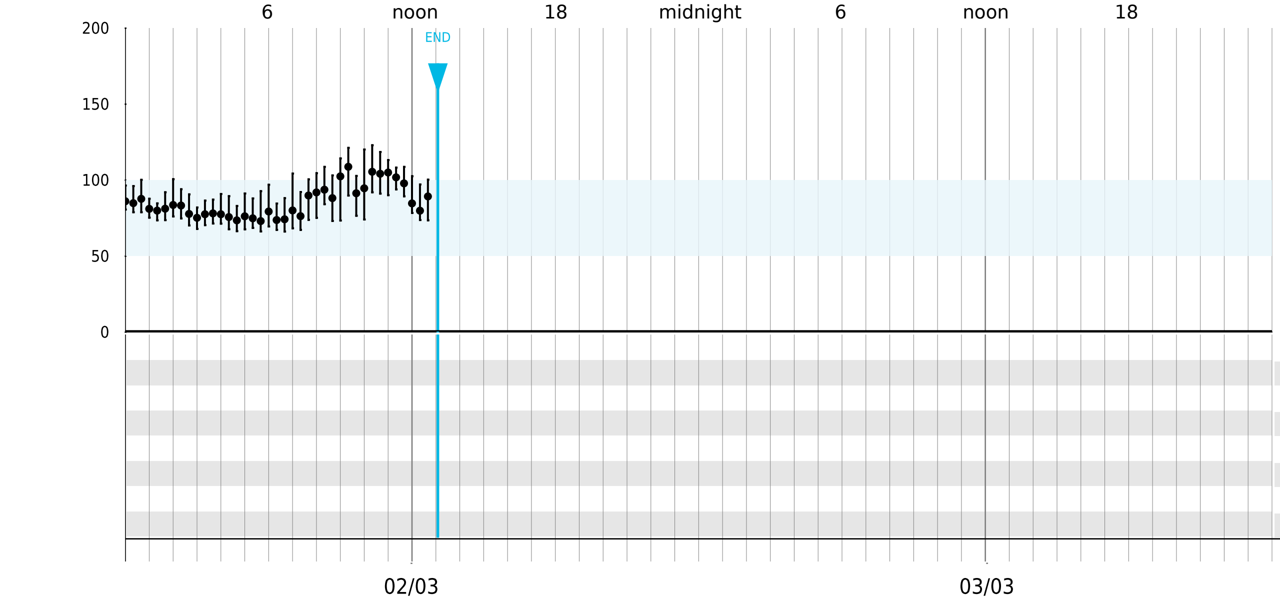

Day 15

0 Patient diary events
0 Patient triggered
0 PVT, VF, TdP
0 VT
0 SVT
0 Pauses
0 % AF/AFL burden
0 AV Block
▼ Unanalyzable ECG

© 2022 iRhythm Technologies, Inc.

Patient Events

Patient Triggered Events, Diary Entries

Note: iRhythm cannot verify the accuracy of the patient-reported information provided below.

= Patient Triggered Marker

= Vertical Zoom

*** = No heart rate will be displayed if insufficient beats of the rhythm are included in the strip.**

None found

© 2022 iRhythm Technologies, Inc.

AF/AFL Burden

Time(hr) in AF/AFL per
■ AF/AFL
□ Sinus/Other

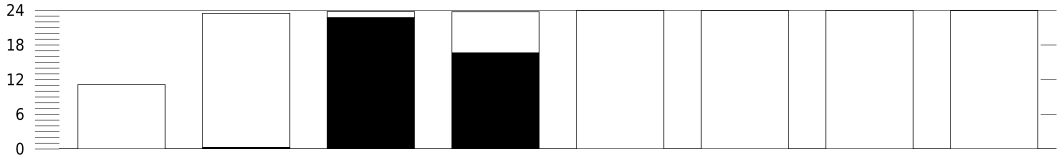

	16/02	17/02	18/02	19/02	20/02	21/02	22/02	23/02
Time in AF/AFL	0h 0m	0h 16m	22h 44m	16h 39m	< 1 min	0h 0m	0h 0m	0h 0m
Unanalyzable Time	12h 51m	0h 31m	0h 12m	0h 15m	0h 2m	0h 2m	0h 2m	0h 3m
**% AF/AFL Analyzable ECG**	**0%**	**1%**	**96%**	**70%**	**< 1%**	**0%**	**0%**	**0%**

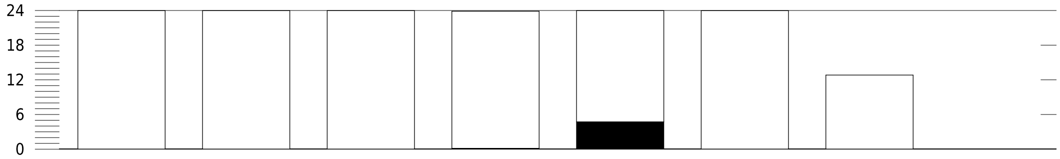

	24/02	25/02	26/02	27/02	28/02	01/03	02/03
Time in AF/AFL	0h 0m	0h 0m	0h 0m	0h 9m	4h 44m	0h 0m	0h 0m
Unanalyzable Time	0h 1m	0h 1m	< 1 min	0h 8m	< 1 min	0h 2m	11h 10m
**% AF/AFL Analyzable ECG**	**0%**	**0%**	**0%**	**< 1%**	**20%**	**0%**	**0%**

AF/AFL
Atrial Fibrillation/Flutter

Total AF/AFL during enrollment
13%
Average heart rate
95 bpm
Heart rate range
62-185 bpm

Note: Heart rates are calculated using a sliding window of 8 beat intervals.

Heart Rate during AF/AFL

Day 6:00 - 22:00 | **Night** 22:00 - 6:00

Heart Rate (bpm)

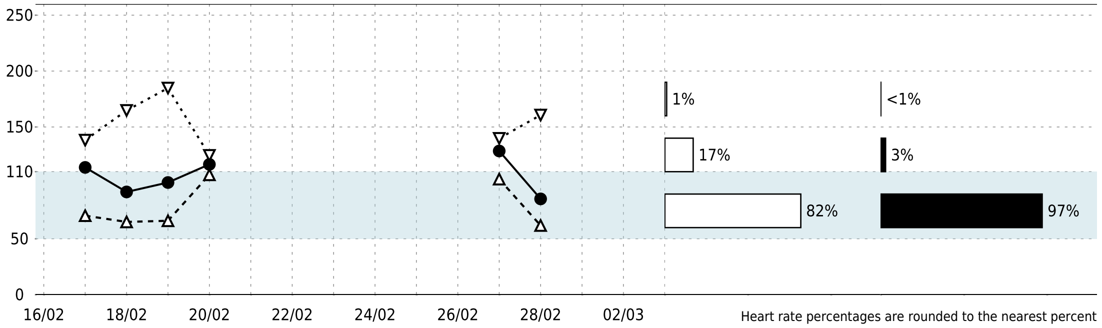

Heart rate percentages are rounded to the nearest percent

AF/AFL Duration

	% of AF/AFL Episodes (30 s)	Episodes	Avg. Duration
1 d+	4%	1	1d 15h
12 - <24 h	0%	0	0
1 - <12 h	4%	1	4h 44m
10 - <60 m	0%	0	0
6 - <10 m	0%	0	0
1 - <6 m	7%	2	2m 51s
30 - <60 s	86%	24	35s

Total Episodes (30 s)
28
Episodes of 6 mins or longer
2
Longest Duration
1 d 15 h

1

AF/AFL with Fastest Heart Rate
19/02/22 09:27:05

Average: 95 bpm
Range: 65-185 bpm

Pt Triggered? ☐ YES ☒ NO

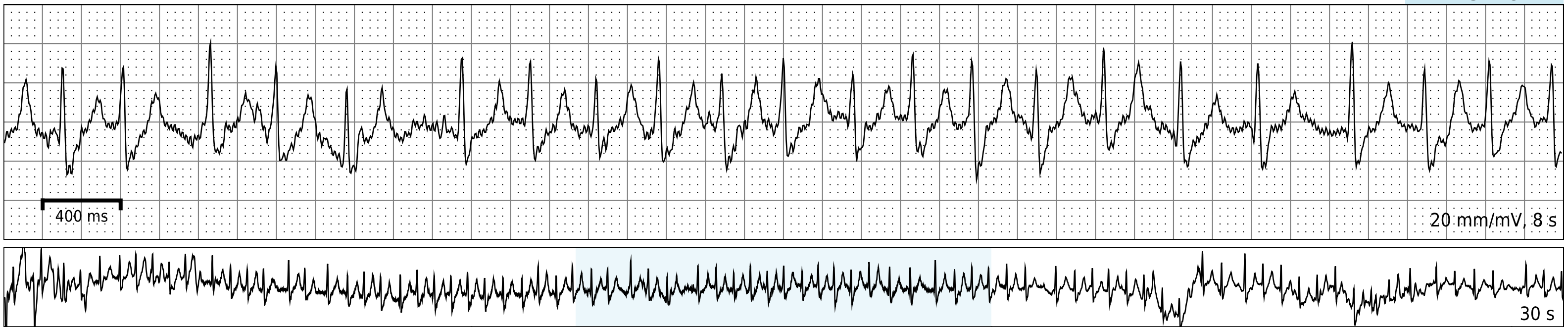

© 2022 iRhythm Technologies, Inc.

2 AF/AFL with Slowest Heart Rate

28/02/22 03:23:49

Average: 86 bpm | **Range:** 62-161 bpm | **Pt Triggered?** ☐ YES ☒ NO

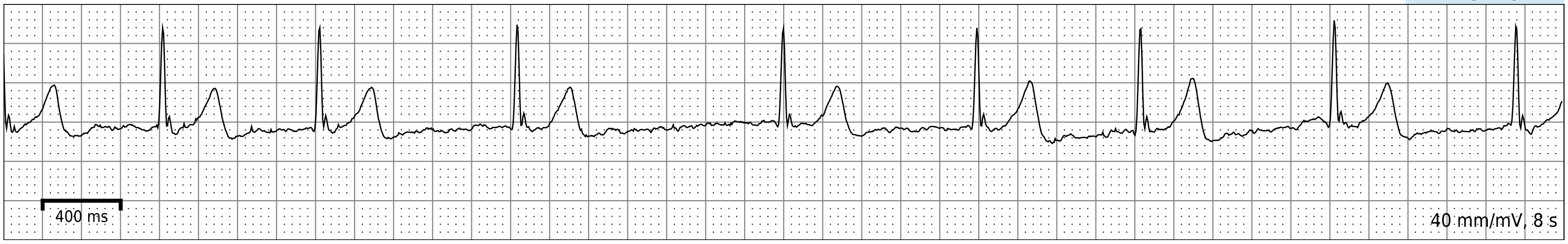

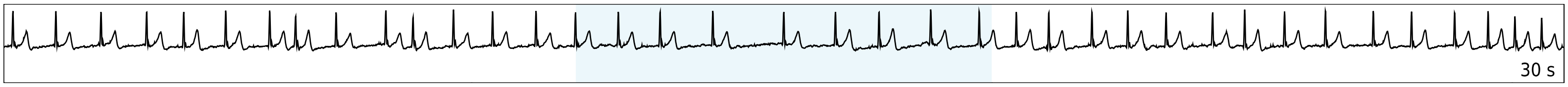

3 AF/AFL with Fastest Avg. Heart Rate

19/02/22 21:31:00

Average: 139 bpm | **Range:** 98-167 bpm | **Pt Triggered?** ☐ YES ☒ NO

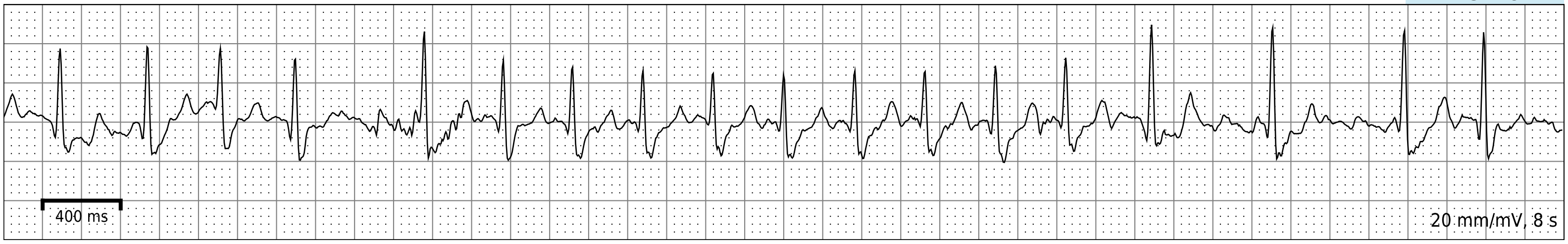

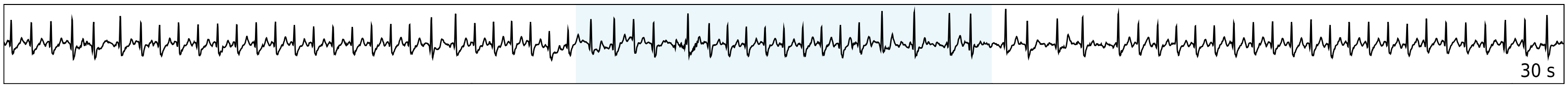

4 Longest AF/AFL Episode

18/02/22 01:12:59

Duration: 1 d 15 h | **Average:** 95 bpm | **Range:** 65-185 bpm | **Pt Triggered?** ☐ YES ☒ NO

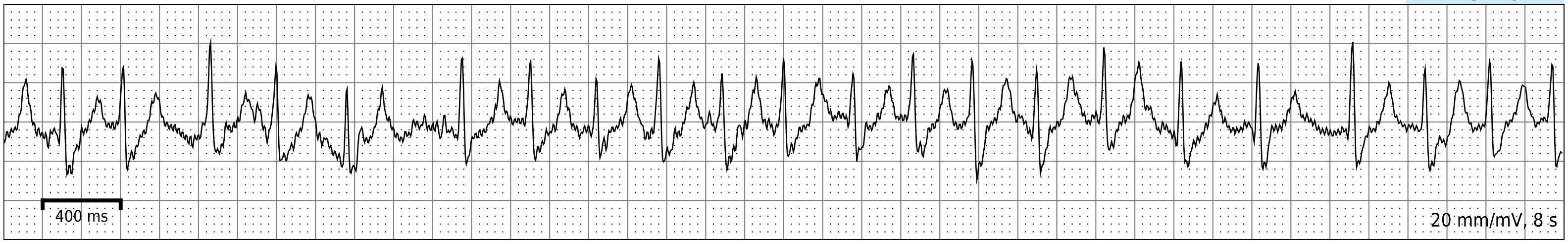

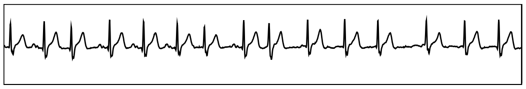
ONSET

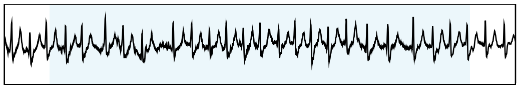
FASTEST

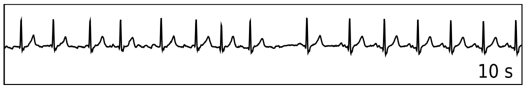

TERMINATION

5 Second Longest AF/AFL Episode

28/02/22 03:09:54

Duration: 4 h 44 m | **Average:** 86 bpm | **Range:** 62-161 bpm | **Pt Triggered?** ☐ YES ☒ NO

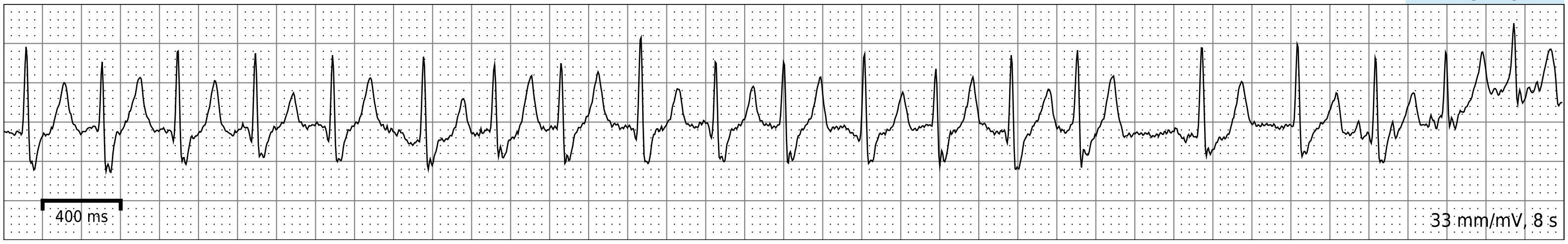

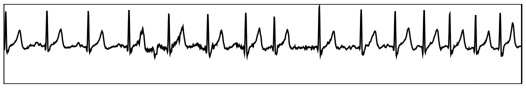
ONSET

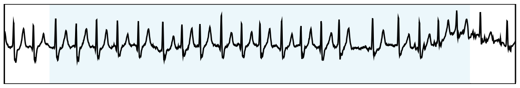
FASTEST

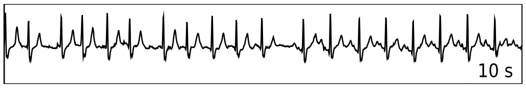

TERMINATION

 © 2022 iRhythm Technologies, Inc.

Additional Strips

1 Minimum Sinus Rate (53 bpm)

22/02/22 06:31:48

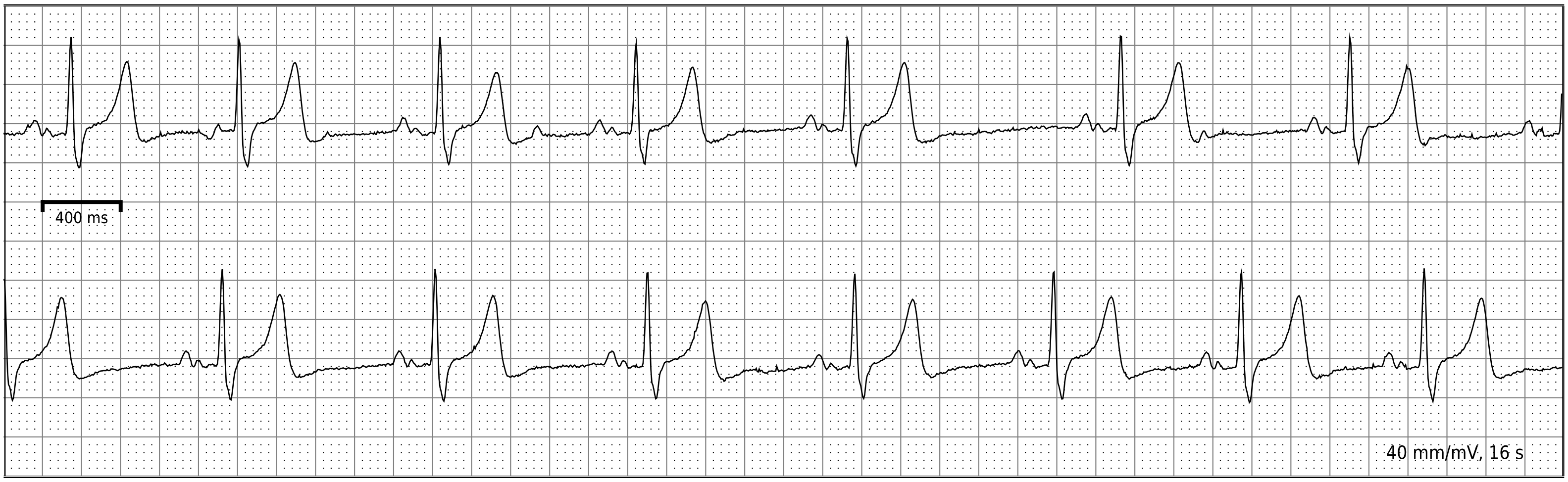

2 Maximum Sinus Rate (123 bpm)

28/02/22 08:19:04

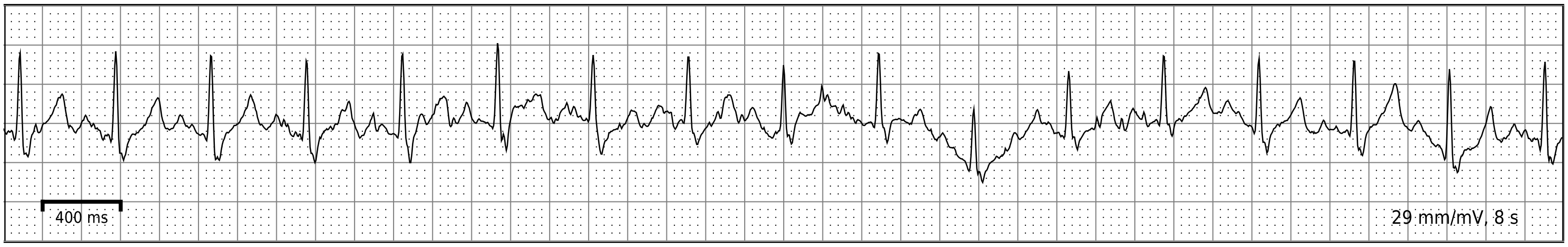

3 Sinus (105 bpm)

28/02/22 08:15:04

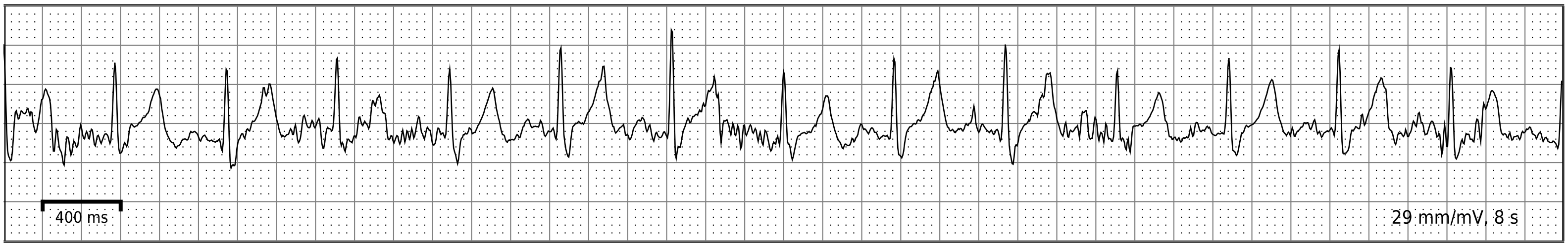

4 Sinus (103 bpm)

28/02/22 09:07:27

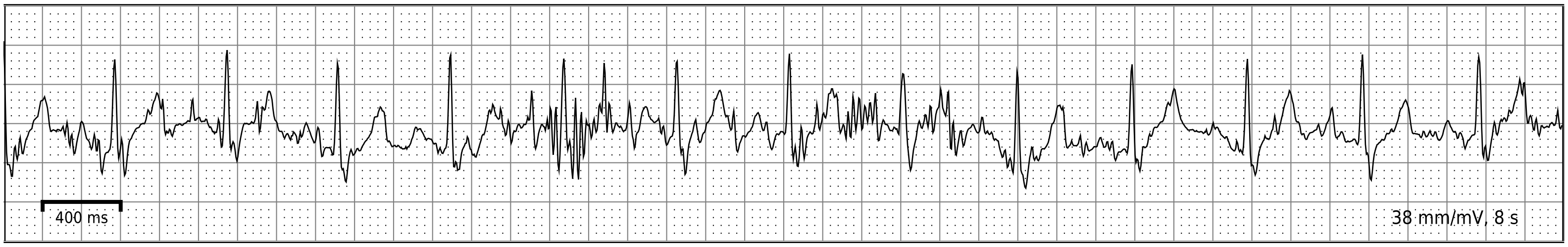

© 2022 iRhythm Technologies, Inc.

5 Initiation of Atrial Fibrillation (79-92 bpm)

18/02/22 01:12:55

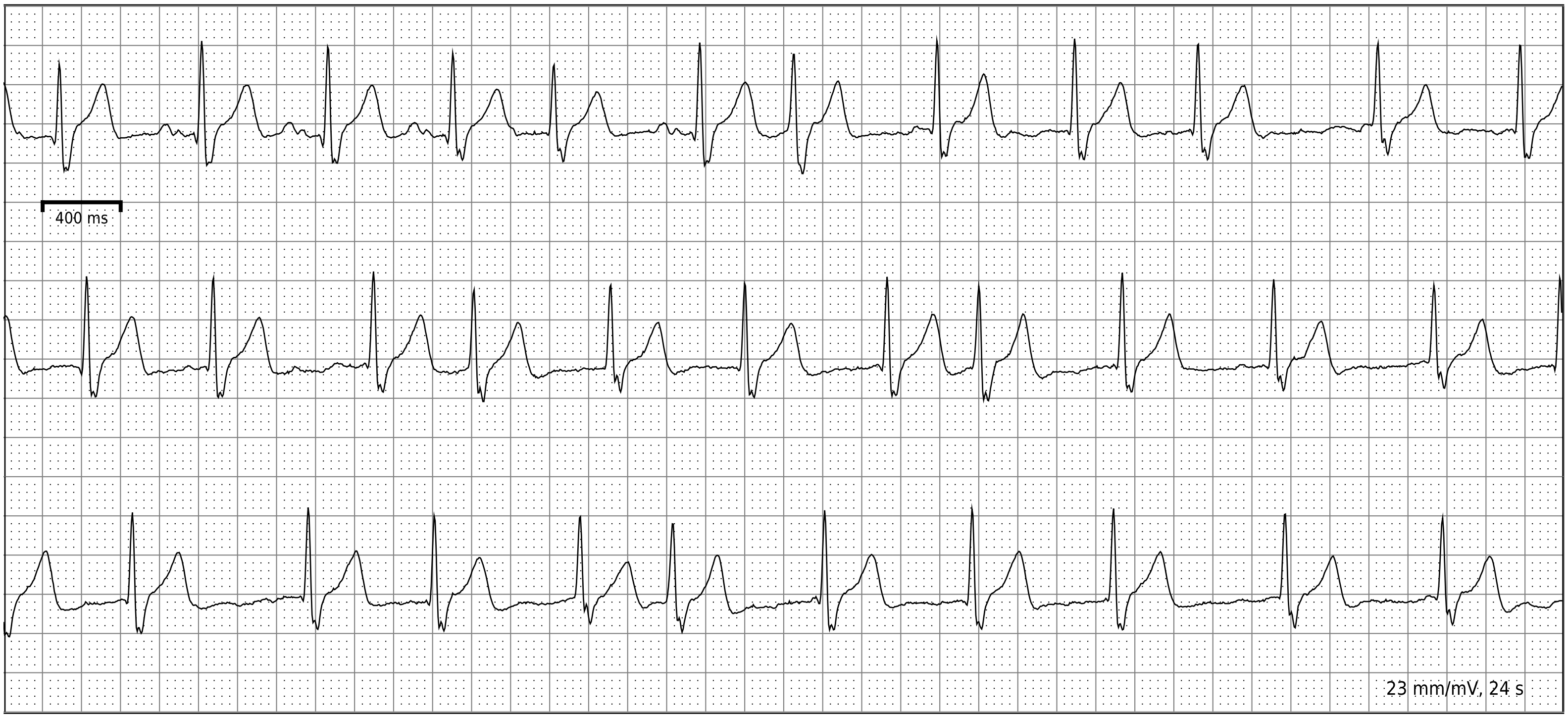

6 Atrial Fibrillation (150-185 bpm)

19/02/22 09:26:57

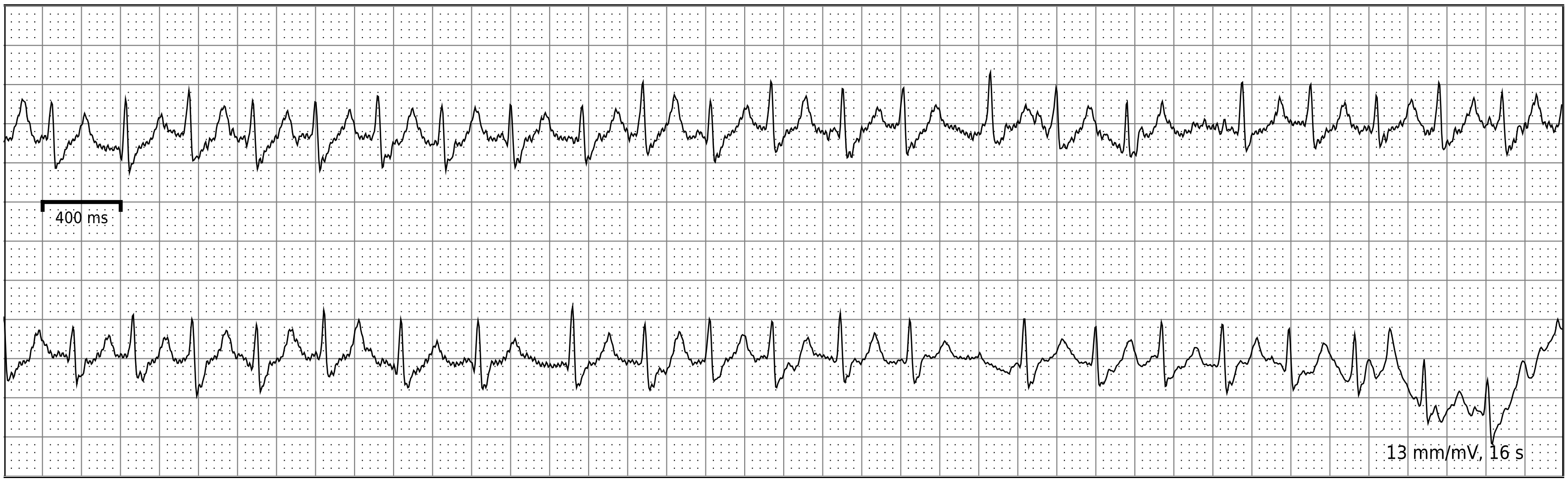

7 Termination of Atrial Fibrillation (95-119 bpm)

19/02/22 16:40:14

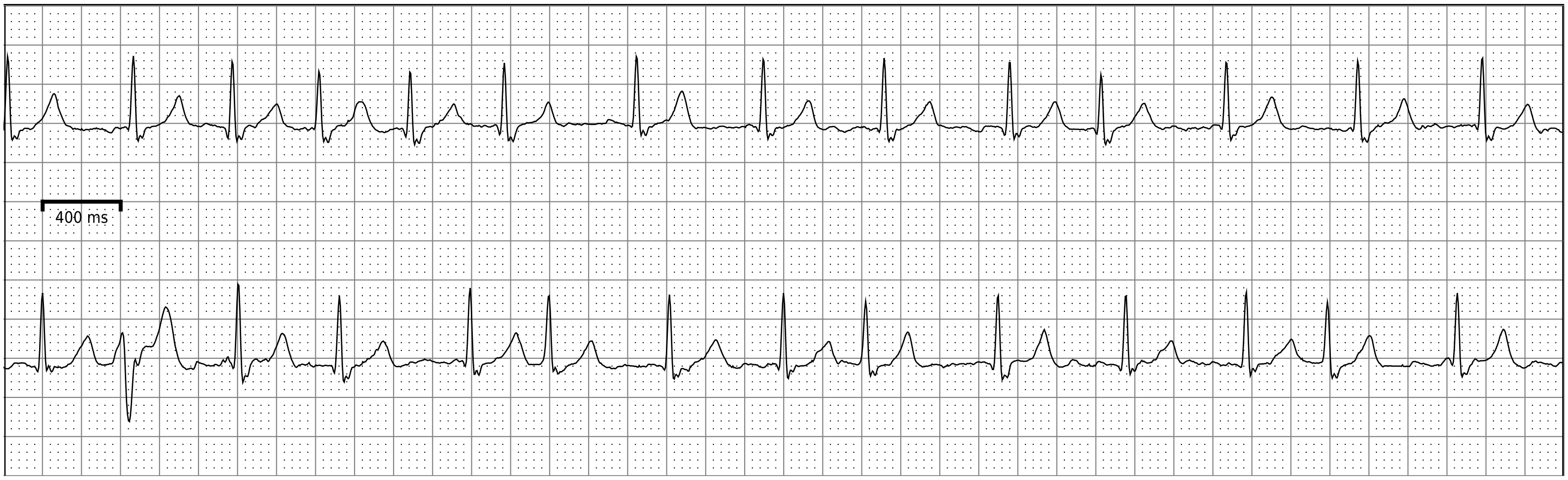

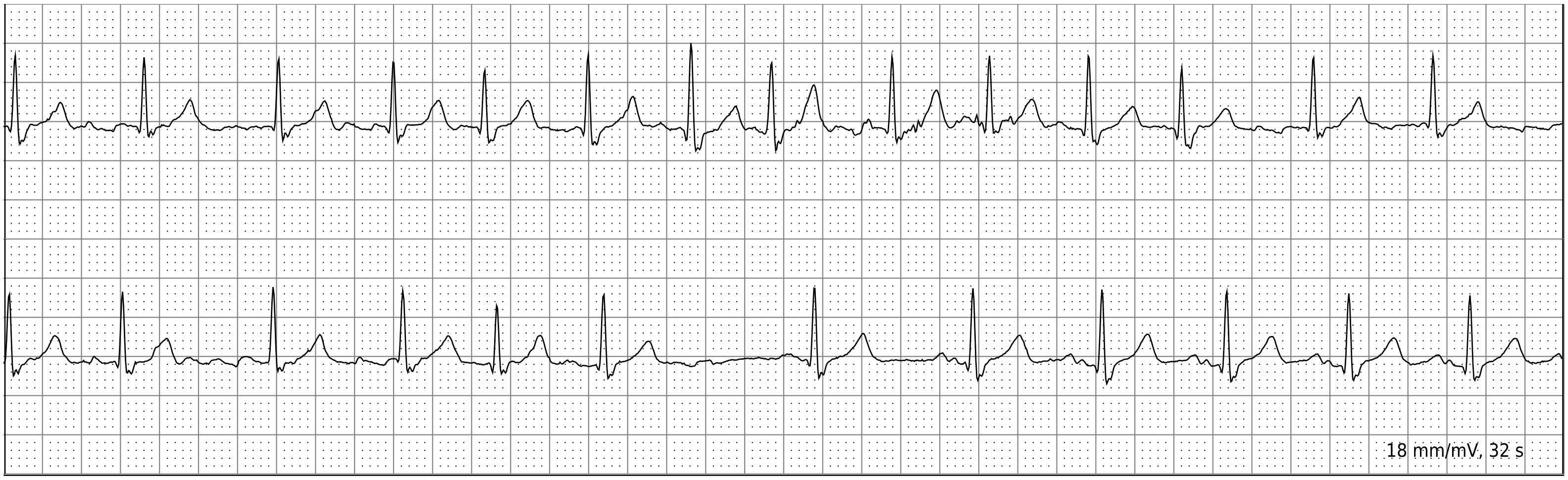

8 Initiation of Atrial Flutter (137-140 bpm)

27/02/22 02:24:30

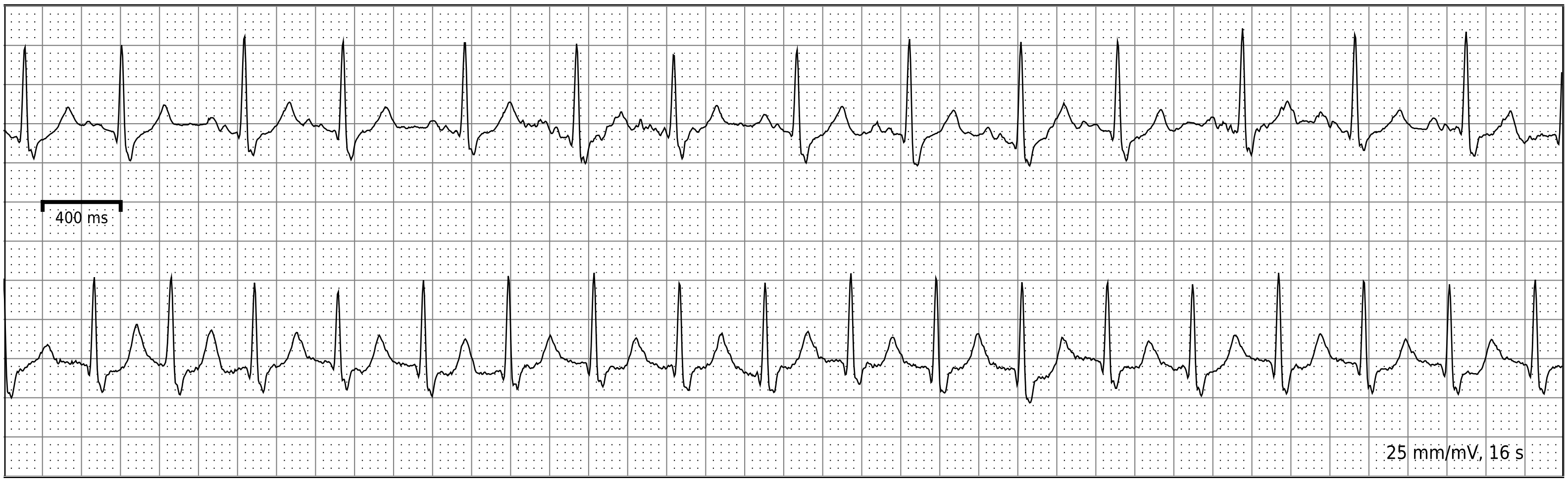

9 Sinus (88 bpm), Atrial Flutter (121-133 bpm)

27/02/22 02:39:13

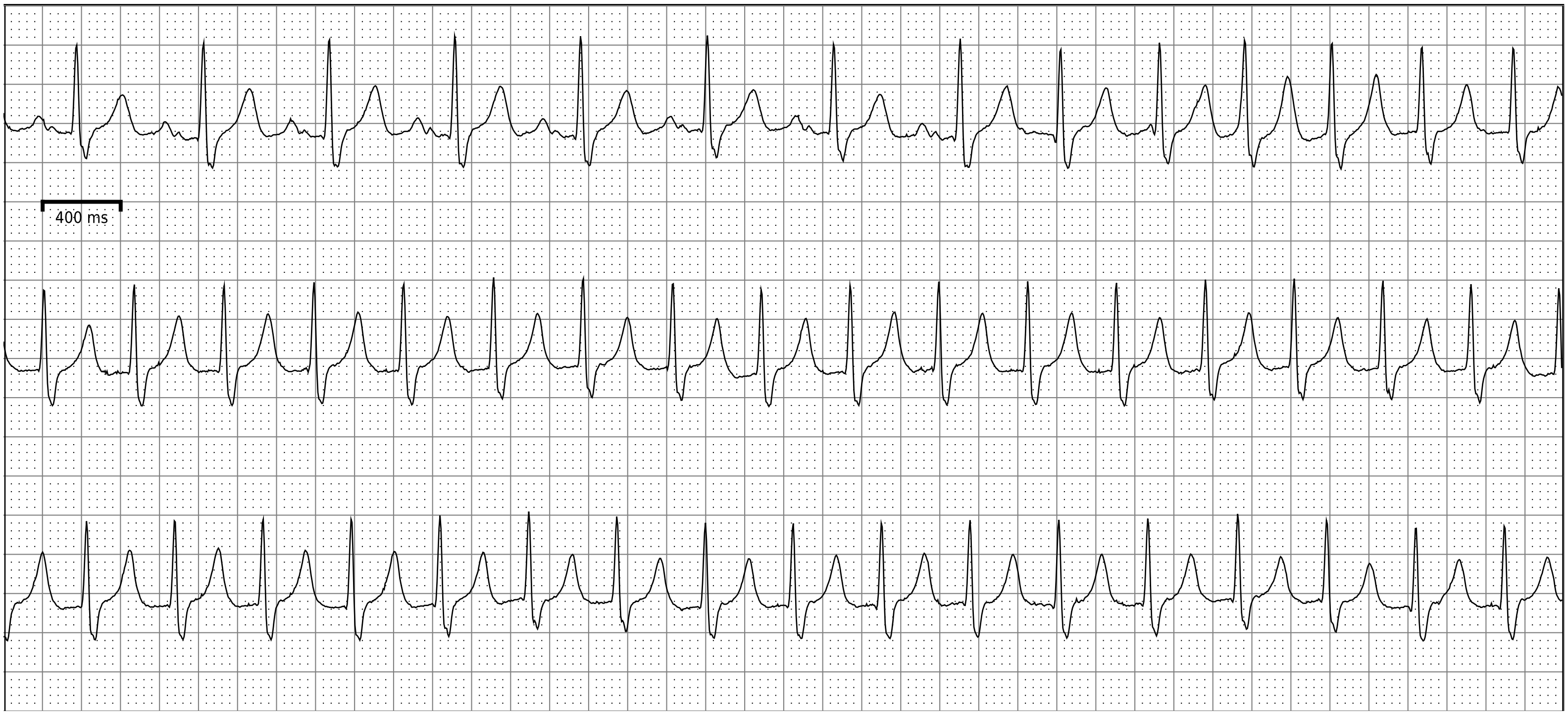

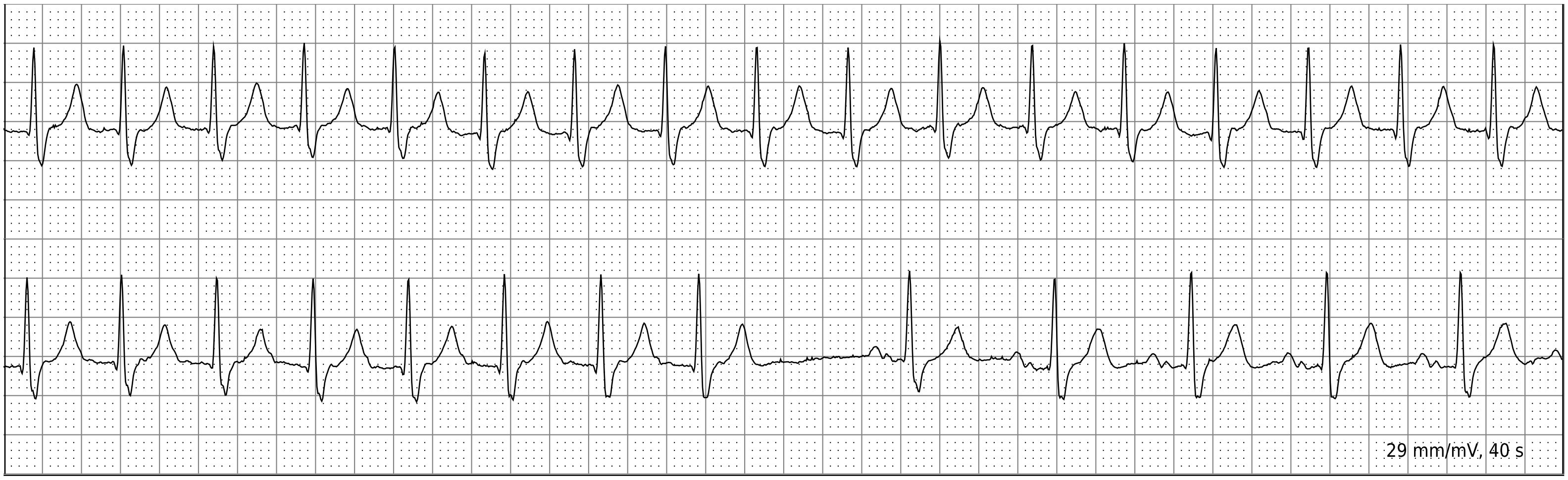

10 Atrial Fibrillation (62-83 bpm)

28/02/22 03:23:37

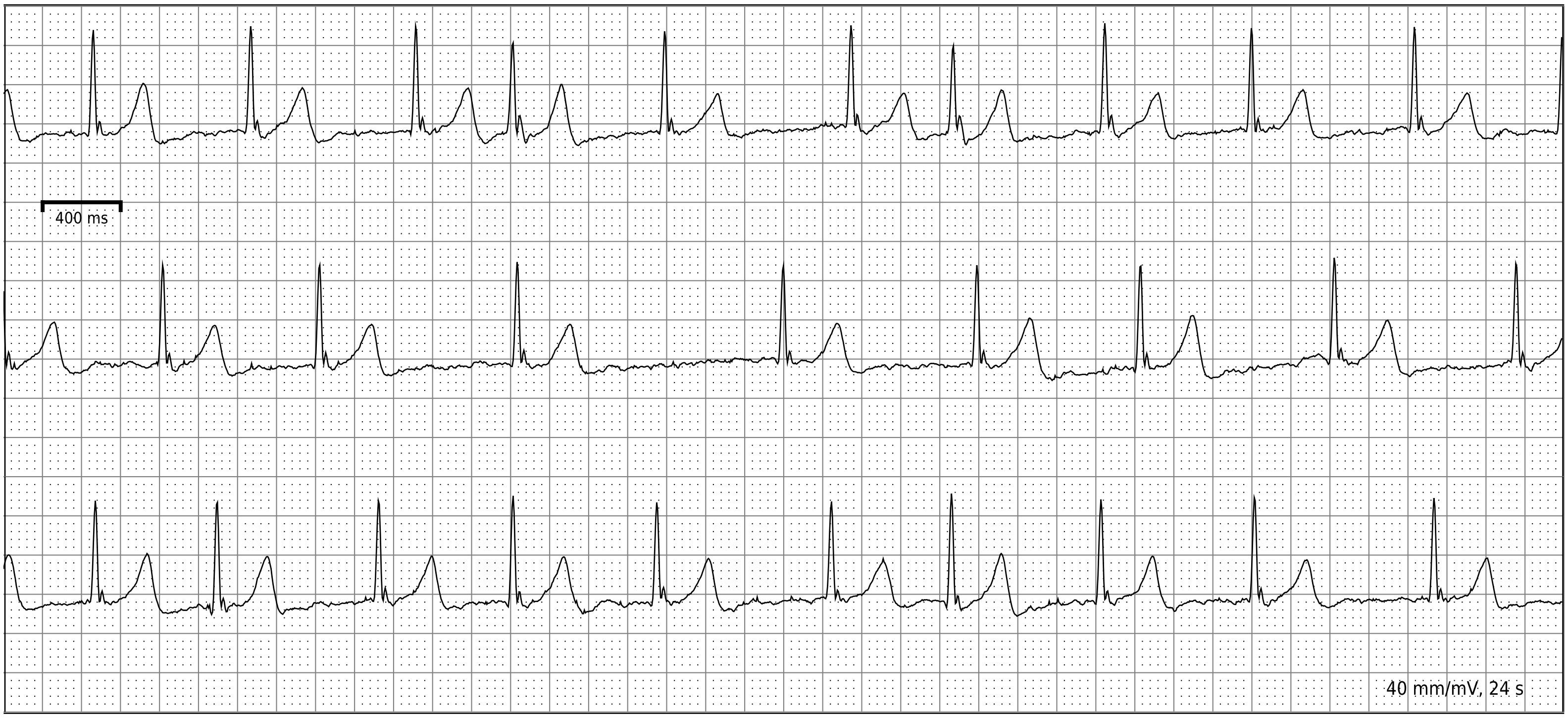

11 Atrial Fibrillation (133-161 bpm)

28/02/22 07:47:51

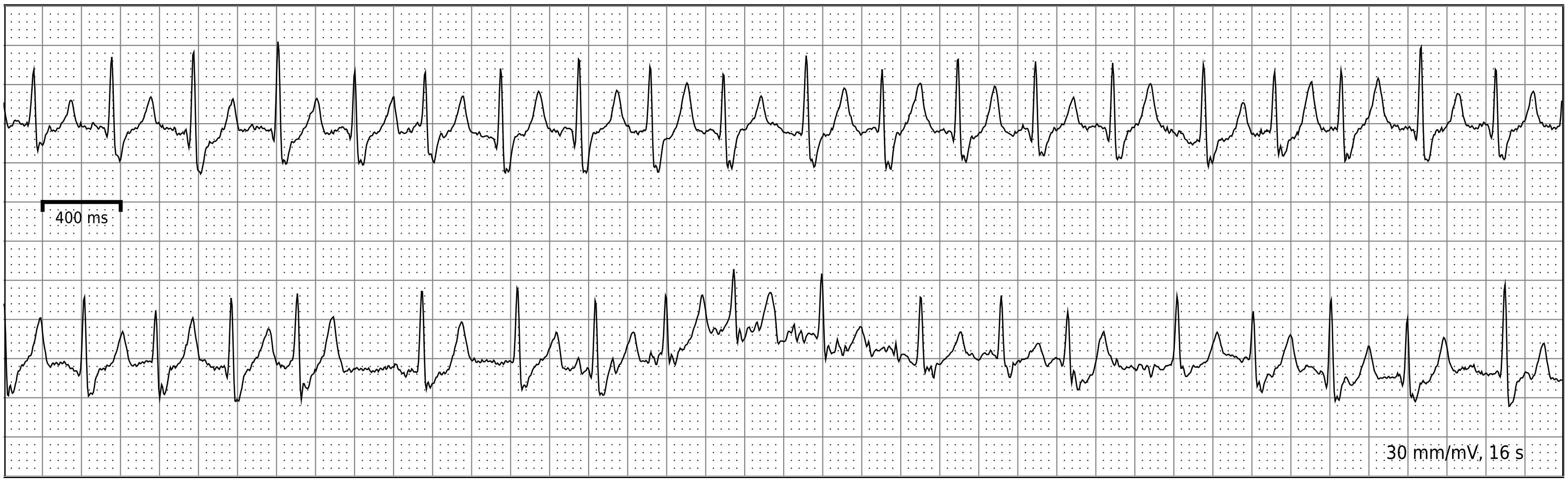

© 2022 iRhythm Technologies, Inc.

12 Sinus (91 bpm), SVE(s), VE(s)

17/02/22 23:06:22

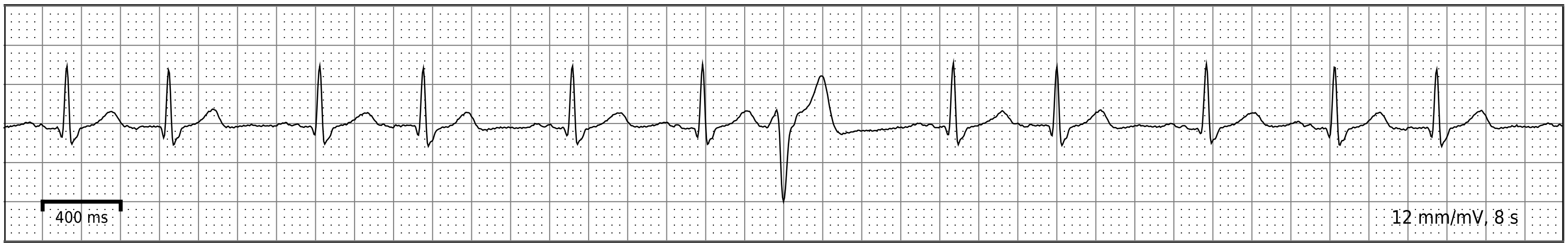

13 Sinus (91 bpm), VE Couplet(s)

20/02/22 17:00:58

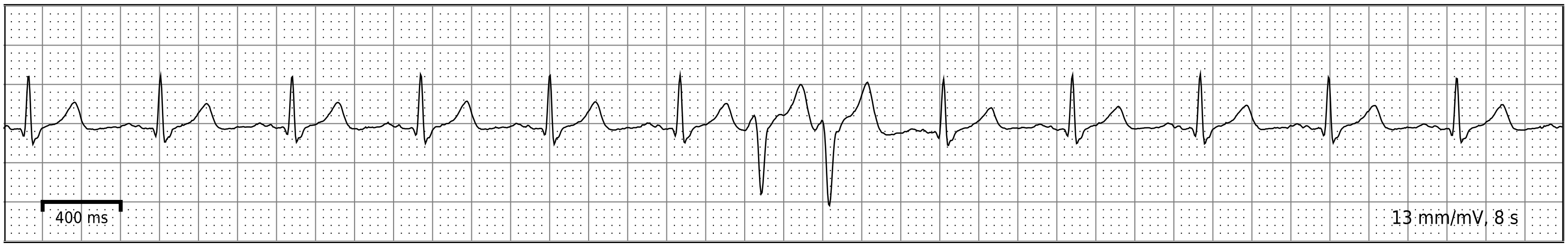

14 Sinus (66 bpm), SVE(s)

01/03/22 16:48:14

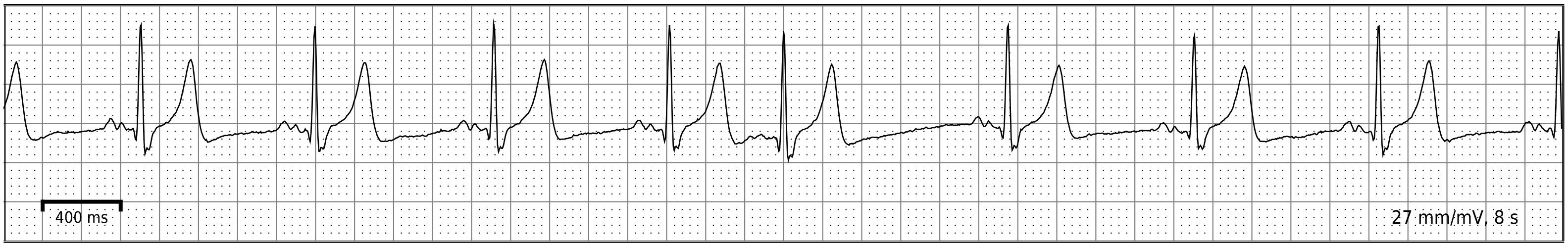

15 Sinus (81 bpm), Run of VEs (64 - 139 bpm)

02/03/22 03:33:31

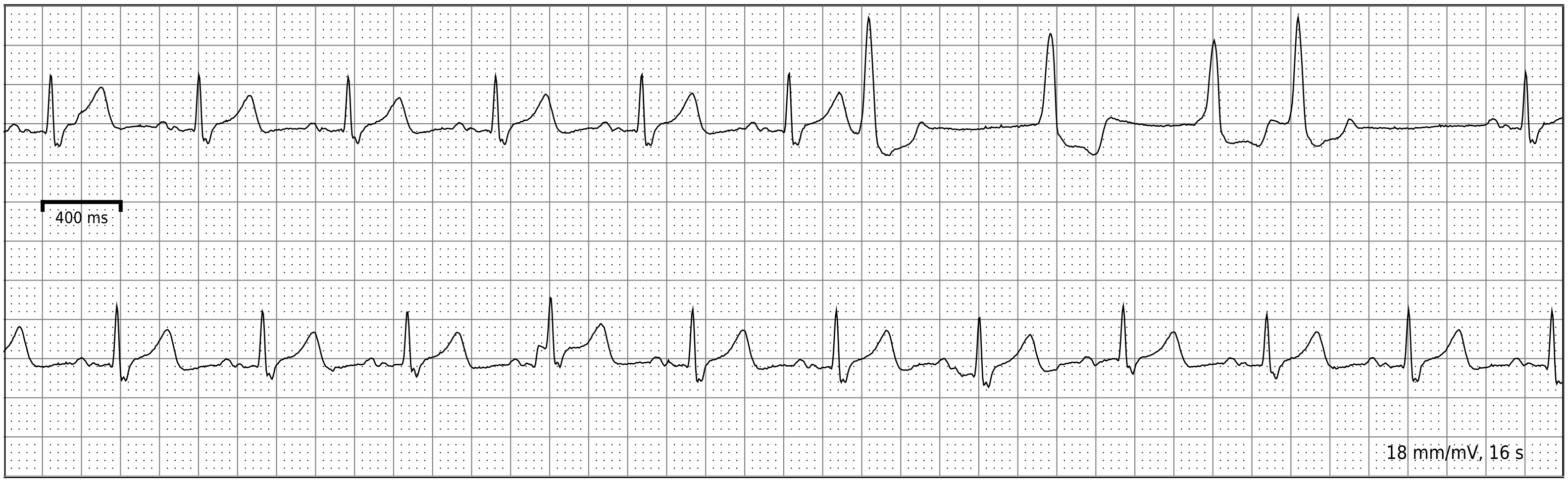

Supplement: Sample_Report [file EMS212696-supplement-Sample_Report.pdf]
