# Supplementary material for: Active Monitoring for AtriaL FIbrillation (AMALFI): Rationale, protocol, and pilot for a pragmatic, randomized, controlled trial of remote screening for asymptomatic atrial fibrillation
Source: Am Heart J. Author manuscript; Available in PMC 2026 Mar 10. (PMC7618845; doi:10.1016/j.ahj.2025.07.004)
Supplement: SAP [file EMS212696-supplement-SAP.docx]

xxxx


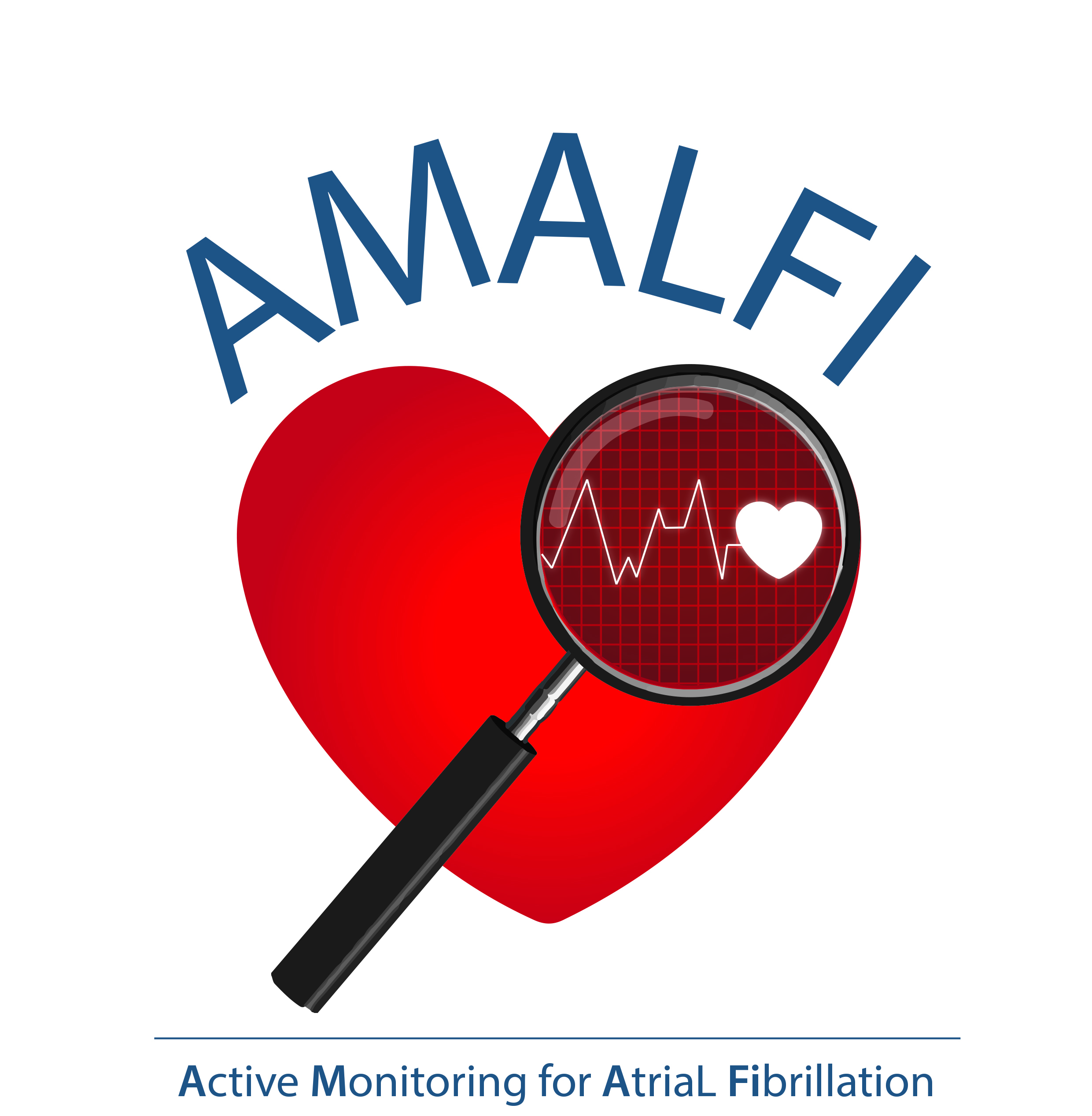


Statistical Analysis Plan

Version 1.2

Date: 26 June 2025

Aligned with protocol version: 2.0, 08 June 2021

IRAS no: 234837

REC ref: 19/LO/0220


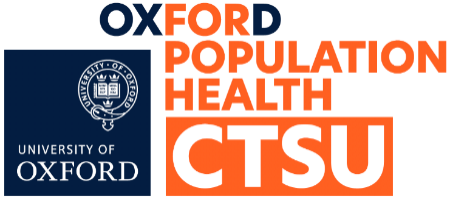
ISRCTN: 15544176

Table of Contents

[1 Roles and responsibilities 4](#_Toc189237835)

[2 Introduction 5](#_Toc189237836)

[3 Background 5](#_Toc189237837)

[3.1 Rationale 5](#_Toc189237838)

[3.2 Trial objectives 5](#_Toc189237839)

[3.3 Trial design 6](#_Toc189237840)

[3.4 Eligibility 6](#_Toc189237841)

[3.4.1 Inclusion criteria 6](#_Toc189237842)

[3.4.2 Exclusion criteria 6](#_Toc189237843)

[3.5 Intervention 6](#_Toc189237844)

[3.6 Outcome comparisons 7](#_Toc189237845)

[3.6.1 Primary outcome assessment 7](#_Toc189237846)

[3.6.2 Secondary outcome assessments 8](#_Toc189237847)

[3.6.3 Exploratory outcomes 8](#_Toc189237848)

[3.6.4 Outcome derivation 8](#_Toc189237849)

[3.7 Hypothesis framework 9](#_Toc189237850)

[3.8 Randomisation 9](#_Toc189237851)

[3.9 Blinding 9](#_Toc189237852)

[3.10 Data collection schedule 9](#_Toc189237853)

[3.10.1 Self-reported medical history 9](#_Toc189237854)

[3.10.2 Primary care data 9](#_Toc189237855)

[3.10.3 Healthcare systems data 10](#_Toc189237856)

[3.10.4 Health-related quality of life data 11](#_Toc189237857)

[3.10.5 End of study 11](#_Toc189237858)

[3.11 Data monitoring 11](#_Toc189237859)

[3.12 Trial reporting 11](#_Toc189237860)

[4 Analysis populations 12](#_Toc189237861)

[4.1 Population definitions 12](#_Toc189237862)

[4.2 Length of follow-up 12](#_Toc189237863)

[4.2.1 Primary care records 12](#_Toc189237864)

[4.2.2 National datasets (medication dispensing) 12](#_Toc189237865)

[4.3 Censoring 12](#_Toc189237866)

[4.3.1 Left-censoring (pre-randomisation records) 12](#_Toc189237867)

[4.3.2 Right-censoring (post-randomisation records) 13](#_Toc189237868)

[5 Descriptive analyses 14](#_Toc189237869)

[5.1 Participant throughput 14](#_Toc189237870)

[5.2 Baseline characteristics of randomised arms 14](#_Toc189237871)

[5.3 Completeness and length of follow-up 16](#_Toc189237872)

[5.4 Adherence to the study intervention 16](#_Toc189237873)

[5.5 Cardiac monitoring findings in the active arm 16](#_Toc189237874)

[6 Comparative analyses 17](#_Toc189237875)

[6.1 Primary outcome 17](#_Toc189237876)

[6.2 Secondary outcomes 17](#_Toc189237877)

[6.3 Sensitivity analyses 17](#_Toc189237878)

[6.4 Exploratory outcomes 18](#_Toc189237879)

[6.4.1 Time to AF detection within a period of 2.5 and 5 years after randomisation 18](#_Toc189237880)

[6.4.2 Time spent with a known diagnosis of AF within a period of 2.5 years after randomisation 18](#_Toc189237881)

[6.4.3 Rates of oral anticoagulation use 18](#_Toc189237882)

[6.4.4 Long-term assessments of the clinical impact of screening 19](#_Toc189237883)

[6.5 Non-randomised comparisons 19](#_Toc189237884)

[6.5.1 Rates of oral anticoagulation between screen-detected and clinically-detected AF (non-randomised assessment) 19](#_Toc189237885)

[6.6 Non-pre-specified exploratory analyses 20](#_Toc189237886)

[6.7 Health economics 20](#_Toc189237887)

[6.8 Power calculations 20](#_Toc189237888)

[6.8.1 Power calculations for the primary outcome 20](#_Toc189237889)

[6.9 Adjustment for baseline characteristics 21](#_Toc189237890)

[6.10 Significance levels and adjustment of p-values for multiplicity 21](#_Toc189237891)

[7 Statistical software 22](#_Toc189237892)

[8 References 22](#_Toc189237893)

[9 Appendices 23](#_Toc189237894)

[9.1 Appendix I: CHA_2_DS_2_-VASc Scoring system 23](#_Toc189237895)

# Roles and responsibilities

***Trial statisticians***

Mrs Georgina Buck, Professor Sarah Parish (up until March 2023), Dr Charlie Harper

Role: To develop the protocol, the statistical analysis plan, and the baseline characteristics and outcomes derivation document, to prepare analysis datasets, and conduct the final comparative analyses

***Trial investigators***

Professor Louise Bowman, Professor Barbara Casadei, Mr Richard Bulbulia, Dr Guilherme Pessoa-Amorim, Dr Rohan Wijesurendra, Dr Christine A’Court, Dr Nicholas Jones

Role: To develop the protocol and the statistical analysis plan, oversee the conduct of the trial, and collect trial data

***Trial IT systems and programmers***

Rijo Kurien, Ola Murawksa

Role: To develop and maintain the randomisation and trial management systems and the clinical databases

# Introduction

This document outlines the proposed analyses concerning results from the AMALFI (Active Monitoring for AtriaL FIbrillation) trial (ISCRTN 15544176), which is investigating the impact of remote screening for asymptomatic atrial fibrillation with 14-day continuous cardiac monitoring on long-term atrial fibrillation (AF) detection.

The planned analyses described here include the trial primary and secondary outcomes, as well as some pre-specified exploratory analyses. Presentation of these results will follow the strategy outlined here. Subsequent, additional, non-pre-specified exploratory analyses will not be bound by this strategy.

Any deviations from the statistical analysis plan will be described and justified in the final report. The analysis will be carried out by identified, appropriately qualified and experienced statisticians, who will ensure the integrity of the data during their processing.

# Background

## Rationale

AF screening has been proposed as a way to prevent stroke by increasing detection of asymptomatic AF, which might otherwise be missed in usual care. Opportunistic screening (e.g. pulse-palpation, 12-lead ECG) during healthcare consultations is recommended by some clinical practice guidelines^1^. However, international taskforces do not recommend systematic AF screening, citing the uncertainty regarding both clinical utility and cost-effectiveness.

AMALFI is testing the hypothesis that AF screening in people aged 65+ with cardiovascular risk factors using 14-day ECG monitoring (Zio XT, iRhythm Technologies, San Francisco, CA, USA) would be cost-effective and lead to increased and earlier AF detection over 5 years. The trial is comparing mail-based AF screening versus usual care in this population.

## Trial objectives

The primary objective is to provide reliable estimates of the effect of remote screening for asymptomatic AF on long-term AF prevalence among individuals aged 65 or more with additional cardiovascular risk factors. This aim is reflected in the primary outcome of proportion of participants with a diagnosis of AF (as recorded in primary care data) within a period of up to 2.5 years after randomisation in each allocation arm.

The secondary objectives are to determine the effect of screening on the mean time spent with a known diagnosis of AF during a period of up to 5 years after randomisation in each arm, i.e. the actionable time period for preventative intervention that would be gained by screening.

Separately, both outcomes specified above will also be assessed by subgroups of age (<80 vs ≥80 years) and sex.

## Trial design

This is a remote, streamlined, open-label, randomised controlled trial. Data collection will be performed mainly through healthcare systems data.

## Eligibility

Eligibility to the study was established based on primary care data available at each participating GP practice, as recorded at the time of recruitment to the trial (i.e. individuals were invited if their medical records suggested they were potentially eligible).

### Inclusion criteria

Individuals were eligible to enter the trial if all of the following were true (at the point of eligibility assessment):

- Male or Female, aged ≥65 years old.
- CHA_2_DS_2_-VASc score ≥3 in men or ≥4 in women (Appendix I)
- Willing and able to give informed consent for participation in the study (NB informed consent was collected remotely by post).

### Exclusion criteria

Individuals could not enter the study if EITHER of the following applied:

- Known to have AF or atrial flutter at the point of eligibility assessment (before invitation)
- Latex allergy (to reduce the risk of skin reactions in individuals with a history of previous reactions to latex, although this was not strictly necessary because latex is not a component of the Zio XT device); NB the existence of a potential previous latex allergy was identified based on an initial screening assessment using a self-reported question (following the initial invitation), and subsequent confirmation via telephone with a study clinician.

## Intervention

Participants allocated to the active (screening) arm of the study received a cardiac monitoring patch (Zio XT) by post, along with instructions on how to apply it, and were asked to wear it for two weeks. Once the monitoring period was over, participants were asked to return the patch by mail to iRhythm for analysis. A full cardiac monitoring report from the analysis of the patch was provided electronically to the study team by iRhythm, and shared with the participant’s GP immediately by email if AF or other findings considered to be clinically relevant (i.e. atrial flutter, sustained ventricular tachycardia or fibrillation; pauses lasting over 6s; complete heart block, Mobitz type II, or high grade atrioventricular block) were found. Separately, the study team received regular structured data extracts from iRhythm including all ECG findings. These data were used to automatically generate a summary results letter (focused on the presence and characteristics of any AF found, including AF burden and maximum and minimum AF rate), which was mailed to each participant and their GP whether or not AF was detected (i.e. GPs were contacted twice if AF was present). Subsequent care was at the discretion of the GP.

Participants allocated to the control (usual care) arm of the study were not requested to perform any actions directly related to the study, with clinical care remaining at the discretion of their GP.

## Outcome comparisons

All comparisons of primary, secondary, and exploratory outcomes involve comparing outcomes among all participants allocated at randomisation to screening versus all participants allocated to the usual care arm (i.e. “intention-to-treat” analysis), unless otherwise specified (i.e. non-randomised comparisons).

### Primary outcome assessment

The primary objective of this study is to provide reliable estimates of the long-term efficacy of AF screening. Conceptually, this may be interpreted as the actionable time window of opportunity for intervention which results, or is gained, from screening. This window is represented by the difference in areas under the cumulative incidence curves (i.e. time with a known diagnosis of AF) between the active and control arms, with those not having AF contributing zero time with known diagnosis (Figure 1). The timeframe for such an assessment of long-term efficacy ought to represent a proposed time interval between screening events if translated into clinical practice - for example 5 years. Hence, the ideal primary outcome for this study would be the amount of time spent with a known diagnosis of AF for each participant during a period of 5 years from randomisation onwards (i.e. the actionable time period). For example, if AF was diagnosed approximately 1 year after randomisation, then the time period spent with a known diagnosis of AF from randomisation up to 5 years is 4 years.


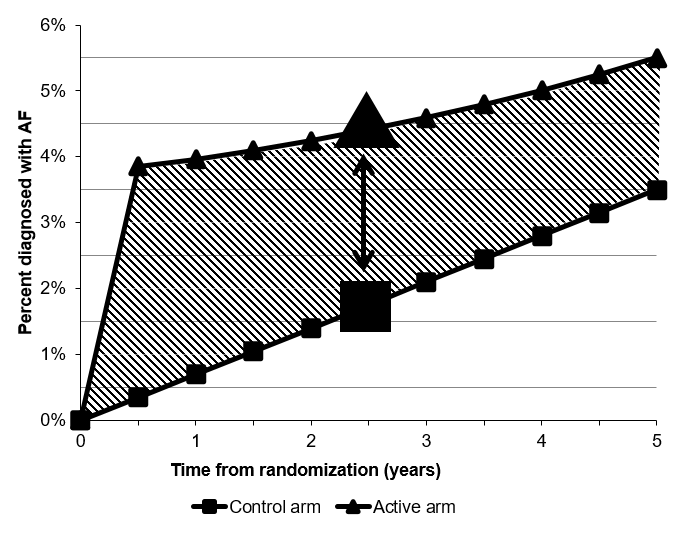
**Figure 1 - Estimated cumulative percent of participants diagnosed with AF**

*Figure legend - Each curve represents the cumulative proportion of new AF diagnoses in the active arm (small triangles) and control arm (small squares). The primary outcome is the proportion of people with AF recorded within primary care records within 2.5 years after randomisation in each arm (large solid triangle and square). The difference between both is represented by the double-headed arrow. The secondary outcome is the mean time spent with an AF diagnosis over a period of 5 years after randomisation (from the date of first post-randomisation AF record until right-censoring at 5 years after randomisation, or death/withdrawal if earlier); this is represented by the area-under-the-curve for each curve (active arm and control arm). The difference between both is the dashed area between the curves.*

However, a more easily interpretable and timely assessment of the expected value of the proposed screening approach can be performed by assessing the proportion with AF detected in each arm within a period of 2.5 years from randomisation – which, based on the statistical modelling performed of the expected AF rates during follow-up, is the largest driver of the expected mean time spent with a known diagnosis of AF within a period of 5 years from randomisation. Further details can be found in the study protocol.

Therefore, the primary outcome assessment will involve an intention-to-treat comparison among all randomised participants allocated to screening versus usual care on **the presence of a record of AF (as recorded in primary care data) occurring between randomisation and 2.5 years after randomisation** (i.e. at around the midpoint of a proposed 5-year screening interval).

### Secondary outcome assessments

The **secondary outcome assessments** for the study will involve intention-to-treat comparisons among all randomised participants allocated to screening versus usual care on:

- the presence of a post-randomisation record of AF within a period of 2.5 years from randomisation (as in the primary outcome assessment) by subgroups of age (<80 and ≥80 years) and, separately, sex
- time spent with a known AF diagnosis within a period of 5 years from randomisation (as described in the previous section), in the overall cohort and in the same subgroups mentioned above.

The secondary outcomes described will be assessed regardless of the results of the primary outcome.

### Exploratory outcomes

A number of exploratory outcomes will also be assessed to complement the information provided by the primary and secondary outcomes. These are listed in detail in Section 6.4.

### Outcome derivation

The detailed derivation of outcomes included in statistical analyses will be described in a separate document.

## Hypothesis framework

For each of the primary and secondary outcomes, the null hypothesis will be that there is no true difference in effect between the two study arms.

## Randomisation

Eligible patients were randomised in a 1:1 ratio to be sent an ECG monitoring patch (Zio XT) to be self-applied and worn for a single 2-week period, versus usual care (i.e. not being sent a patch), by the central computer in CTSU, using a minimisation algorithm to ensure balance by important baseline variables (age [<75, ≥75 years], sex, and residual CHA_2_DS_2_VASc score excluding age and sex [residual score 0-1, 2, 3-6]).^2^

## Blinding

This is an open-label study. As such, participants, their GPs, and the trial team are aware of study arm allocation. However, while the study is in progress, access to tabular results of study outcomes by randomised allocation will not be available to the research team, the chief investigators, trial statisticians, or GPs. Patch monitoring data may be interrogated to ascertain the rate of new AF diagnoses found via patch as the trial progresses. However, neither patch data nor records of study arm allocation will be combined with clinical data from other sources in a way that could potentially lead to unblinding prior to preparation and production of the main trial analyses.

## Data collection schedule

### Self-reported medical history

Upon inclusion in the trial, each participant was required to complete and return by post a questionnaire which asked about relevant parts of their medical history to establish eligibility. These included the components of the CHA_2_DS_2_-VASc score, and a question about previous allergies to latex (the latter used as a screening assessment to identify patients who might not be eligible for the trial). Self-reported information on the components of the CHA_2_DS_2_-VASc score was used to inform the minimisation randomisation algorithm and will be used in descriptive analyses.

### Primary care data

Extracts from electronic primary care records will be performed via each of the participating GP practices at three time points (counted from the date of randomisation of the last participant randomised at each practice):

- at least 1 year after randomisation (to establish data extraction feasibility and allow collection of preliminary data);
- at least 2.5 years after randomisation (to support analysis of the outcomes specified at 2.5 years after randomisation); and
- at least 5 years after randomisation (for analysis of the outcomes specified at 5 years after randomisation).

Data extracted from primary care records will include information on ethnicity, body mass index, medical diagnoses, symptoms, medication prescriptions, referrals to secondary care, procedures and diagnostic tests, and appointments/contacts with practice staff (for clinical or administrative reasons).

### Healthcare systems data

Healthcare systems data (in addition to primary care records) will be requested from medical databases including those held by NHS England and equivalent bodies. This will include the following datasets as specified in the table below.

| **Dataset** | **Event type** | **Reference** |
| --- | --- | --- |
| Medicines dispensed in Primary Care NHS Business Services Authority data product (Dispensing dataset) | Medication dispensing in the community | https://digital.nhs.uk/data-and-information/data-tools-and-services/data-services/medicines-dispensed-in-primary-care-nhsbsa-data |
| Civil Registrations of Death | Death registrations | https://digital.nhs.uk/services/data-access-request-service-dars/dars-products-and-services/data-set-catalogue/civil-registrations-of-death |
| Emergency Care Dataset (ECDS) | A&E department attendance | https://digital.nhs.uk/data-and-information/data-collections-and-data-sets/data-sets/emergency-care-data-set-ecds |
| Hospital Episode Statistics Outpatients (HESOP) | Outpatient appointments in secondary care | https://digital.nhs.uk/services/hospital-episode-statistics |
| Hospital Episode Statistics Admitted Patient Care (HESAPC) | Hospitalisations |  |
| Hospital Episode Statistics Critical Care (HESCC) | Critical care admissions |  |

These data will be used to complement primary care data to identify relevant baseline characteristics, characterise medication and healthcare use, and to collect other information of interest which is expected not be captured adequately in primary care data (such as deaths and need for hospitalisation, for example due to a bleed).

The datasets specified will be requested from NHS England or other institutions on an annual basis, starting from 2022 and at least until the end of 2027 (i.e. during the scheduled study period for the assessment of the primary and secondary outcomes). Data may continue to be requested for up to 25 years from randomisation for all surviving participants (unless they have withdrawn consent).

Further information on use of linked data, namely specific derivation procedures and which data points each dataset will contribute to, will be specified in a separate document.

### Health-related quality of life data

Health-related quality of life data will be collected using self-reported EQ-5D-5L questionnaires. These will be mailed to the entire cohort simultaneously, at two time points: 1) once recruitment to the trial is complete, and 2) after the results of the primary outcome are published. Further details on the use of these data will be specified in a separate health-economics analysis plan.

### End of study

The main trial period is scheduled up until 5 years from randomisation. The end of study is defined as the date of the last electronic data download at 5 years after randomisation (from either central registries, including NHS England or others, or from a participating GP practice). However, it is intended that data may continue to be requested from NHS England (for up to 25 years after randomisation) in order to undertake longer-term exploratory assessments of the clinical impact of allocation to screening.

## Data monitoring

The trial does not have a formal data monitoring committee, and no interim data monitoring assessments are planned (to investigate either safety or efficacy).

Specific procedures for storing and handling data collected as part of the trial will be described in a separate data management document.

## Trial reporting

The trial will be reported according to the principles of the CONSORT statement.^3^ It is anticipated that the study will produce one publication describing the study rationale, design, and baseline characteristics of recruited participants, one publication focused on analyses of the primary and secondary outcomes at 2.5 years after randomisation, and a subsequent publication with results from the secondary outcomes assessed at 5 years after randomisation. However, further publications may be produced.

# Analysis populations

## Population definitions

Unless otherwise specified, all comparative analyses between study arms will use the intention-to-treat principle – i.e. including all randomised participants, regardless of whether they subsequently adhered to the randomised allocation.

## Length of follow-up

### Primary care records

Length of follow-up in the primary care records will be calculated as time from randomisation to the earliest of:

- **death**;
- **withdrawal of consent** (complete withdrawal of consent, or withdrawal of permission for GP to share information about important illnesses);
- **loss to follow-up in primary care records**, defined for each participant as the date of a known change of GP practice to a practice not taking part in AMALFI (identified from either direct reporting to the study team, or GP practice codes assigned to dispensing records in the nationwide dispensing dataset); or
- **study day 913 or study day 1826** (where day of randomisation is study day 0) – i.e. after 2.5 or 5 years have elapsed – according to the timeframe specified for assessment of each outcome.

### National datasets

Length of follow-up in the national datasets (section 3.10.3) will be calculated as time from randomisation to the earliest of:

- **death**;
- **withdrawal of consent** (complete withdrawal of consent, or withdrawal of permission for NHS England) to share information about important illnesses);
- **study day 913 or study day 1826** (where day of randomisation is study day 0) – i.e. after 2.5 or 5 years have elapsed.

## Censoring

### Left-censoring (pre-randomisation records)

#### Primary and secondary outcomes

In the event that AF diagnoses occurring pre-randomisation are identified, such diagnoses will not count towards the primary or secondary outcomes, but their numbers will be reported. For the avoidance of doubt, individuals for whom a pre-randomisation AF diagnosis is identified will be included in the intention-to-treat analysis, in the study arm they were allocated to. These individuals, who had AF first recorded before randomisation, will only be counted as having an AF endpoint event if they also have a subsequent record of AF post-randomisation. Pre-randomisation records will be discarded as it is considered that at least some of these could have been wrongly entered into the primary care records or entered retrospectively, otherwise these patients would not have been identified as potentially eligible following the electronic eligibility search performed.

#### Outcomes of oral anticoagulation exposure

As the nationwide dataset on medication dispensing only provides the calendar month and year in which a dispensing record occurred, with no exact dates, it is not possible to discern whether anticoagulation during the month of randomisation is prior to or post randomisation. Therefore, for any outcomes assessing post-randomisation exposure to oral anticoagulation, only records occurring in the months after the month of randomisation will contribute. Records of medication use prior to or during the month of randomisation will be counted as medication prior to randomisation.

### Right-censoring (post-randomisation records)

#### Primary outcome and subgroup analyses of the primary outcome

No censoring will be applied when assessing the primary outcome (the presence of a record of AF, as recorded in primary care data, occurring between randomisation and 2.5 years after randomisation) or subgroup analyses of the primary outcome. Further details on the planned hypothesis testing approach for these outcomes are included in Section 6.1.

#### Secondary outcomes of time spent with AF diagnosis at 5 years

For the secondary outcome of time spent with a known AF diagnosis within a period of 5 years after randomisation, and its corresponding subgroup analyses, right-censoring will be applied at the earliest of death, study withdrawal, or on study day 1826.

For the avoidance of doubt, while information on fact and timing AF diagnoses will only be captured from primary care records, the analysis of time spent with (i.e. *after*) a known AF diagnosis spans from the date of diagnosis to date of death. Therefore, while the *length of follow-up* is defined by the length of primary care data collection, the relevant *right-censoring* time point is the earliest of date of death, withdrawal, or on study day 1826.

Additionally, a data imputation procedure will be specified at a later stage to handle loss to follow-up in primary care records in participants *without* a known diagnosis of AF by the time of loss to follow-up.

#### Outcomes of oral anticoagulation exposure

For any outcomes assessing exposure to oral anticoagulation, right-censoring will be applied at the earliest of calendar month of death, withdrawal, or the 30th calendar month (for 2.5 year analyses) or 60th calendar month (for 5 year analyses) post-randomisation.

# Descriptive analyses

## Participant throughput

The flow of participants through the trial will be summarised using a CONSORT diagram. The flow diagram will describe the numbers of participants invited, randomly allocated, adherence to patch, withdrawals of consent, and losses to follow-up (e.g. moved GP, death during follow-up).

## Baseline characteristics of randomised arms

The following characteristics, derived from self-reported medical history on the randomisation form and the electronic records, will be described separately for participants in each arm, at randomisation:

- Age (mean age, and grouped by <75, 75 to 79 and ≥80 years at randomisation)
- Sex
- Ethnicity (split into White, Black, Asian, Mixed/Other, and missing)
- Body mass index (mean and grouped by <25, 25 to <30, ≥30 kg/m^2^, and missing)
- CHA_2_DS_2_VASc score (median and grouped as score 3, 4, or 5 or more)
- CHA_2_DS_2_VASc components
- Chronic kidney disease (stage 3 or higher vs no CKD)
- Use of oral anticoagulation (prior to or in the month of randomisation)
- Use of other relevant treatments (prior to or in the month of randomisation):
  - Statins
  - Aspirin or dipyridamole
  - P2Y12 inhibition
  - RAAS inhibition
  - Beta-blockers
  - Diuretics
  - Calcium-channel blockers (diltiazem, verapamil, dihydropyridines)
  - Insulin
  - Proton-pump inhibitors or H2-antagonists

The number and percentage will be presented for binary and categorical variables. The mean and standard deviation or the median and the interquartile range will be presented for continuous variables, as appropriate. Counts of participants with missing data for each parameter will also be presented, with missing data defined as unavailability in the linked healthcare systems data required to ascertain each parameter (e.g. primary care data for body mass index; medication dispensing data for medications; both primary data and HES data for ethnicity). A shell table is provided below:

|  | | **Study allocation** | |
| --- | --- | --- | --- |
| **Characteristic** | **Overall,**  **N = 5,040** | **Patch,**  **N = 2,520** | **Control,**  **N = 2,520** |
| Age, mean (SD) |  |  |  |
| - <75 |  |  |  |
| - 75-79 |  |  |  |
| - ≥80 |  |  |  |
| Female sex |  |  |  |
| Ethnicity |  |  |  |
| - White |  |  |  |
| - Black |  |  |  |
| - Asian |  |  |  |
| - Mixed/Other |  |  |  |
| - Missing |  |  |  |
| Body mass index (BMI), mean (SD) |  |  |  |
| - <25 kg/m^2^ |  |  |  |
| - 25 to <30 kg/m^2^ |  |  |  |
| - ≥30 kg/m^2^ |  |  |  |
| - Missing |  |  |  |
| CHA_2_DS_2_VAsc score, median (IQR) |  |  |  |
| - 3 |  |  |  |
| - 4 |  |  |  |
| - ≥5 |  |  |  |
| Heart failure |  |  |  |
| Hypertension |  |  |  |
| Diabetes |  |  |  |
| Stroke or TIA |  |  |  |
| Thromboembolism |  |  |  |
| Myocardial infarction |  |  |  |
| Peripheral arterial disease |  |  |  |
| Chronic kidney disease (stage ≥3) |  |  |  |
| Medication exposure prior to randomisation |  |  |  |
| - Oral anticoagulation |  |  |  |
| - Statin |  |  |  |
| - Aspirin or dipyridamole |  |  |  |
| - P2Y12 inhibition |  |  |  |
| - RAAS inhibition |  |  |  |
| - Beta-blockers |  |  |  |
| - Diuretics |  |  |  |
| - Calcium-channel blockers |  |  |  |
| - Insulin |  |  |  |
| - Proton-pump inhibitors or H2-antagonists |  |  |  |

*Number of participants with no available primary care data: n= XX*

*Number of participants with no available nationwide dispensing data: n=XX*

## Completeness and length of follow-up

All reasonable efforts will be taken to minimise loss to follow-up. While data collection is entirely electronic, efforts undertaken to minimise loss to follow-up (in primary care records) include close communication with GP practices to ensure data are extracted and transferred in a timely manner, troubleshooting issues with data extraction and transfers, and cross-checking numbers of participants with data received from each practice with those expected based on recruitment records (and investigating and correcting any discrepancies). Losses to follow-up are therefore expected to be few given the use of remote data collection procedures and a combination of data from local primary care records and a wealth of nationwide datasets.

The number and percentage of participants with follow-up information to day 913 (2.5 years) and day 1,826 (5 years) will be reported for each relevant comparison, as well as the average length of follow-up time available (as further described in Section 4.2). These will be reported separately for primary care data (to be used for the primary and secondary outcomes) and nationwide dispensing data (to be used for exploratory outcomes of oral anticoagulation exposure).

## Adherence to the study intervention

The number and proportions of participants allocated to the active (screening) arm who wore a patch and for whom any length of patch monitoring data is available will be reported, and taken to represent overall adherence to the study intervention. The median time spent wearing a patch and the median proportion of patch data suitable for analysis will also be reported, as well as the median time between randomisation and the date the patch was activated (for those who wore it). Additionally, baseline characteristics of participants in the active arm will be reported separately for those who wore vs those who did not wear a patch. Reasons for non-adherence to patch will also be reported.

## Cardiac monitoring findings in the active arm

Heart rhythm findings detected by the ECG patch will be reported for participants in the active arm of the study who wore a patch. These will include:

- Rate of new AF detection via patch during the monitoring period (number and proportion)
- Characteristics of AF detected via patch
  - Burden (i.e. proportion of time spent in AF during the monitoring period)
  - Longest AF episode duration (in hours)
  - Maximum heart rate during AF episodes
  - Minimum heart rate during AF episodes
  - Time to first AF episode during the monitoring period (in days)
- Frequency of other arrhythmias found through monitoring (e.g. atrial flutter, supraventricular tachycardias, non-sustained and sustained ventricular tachycardias, atrioventricular block of different degrees)

Additionally, for participants with AF detected via patch, the time between patch reporting date and the date of the first AF record in primary care records will also be reported.

# Comparative analyses

## Primary outcome

The chi-square test will be used to compare the proportion of participants with a record of AF in primary care data within a period of 2.5 years after randomisation in all those randomised to screening versus all those randomised to usual care. Proportions in each arm will be presented, along with the ratio of proportions and the associated 95% confidence interval.

Individuals who had AF recorded before randomisation will only be counted as having an AF endpoint event if they also have a subsequent record of AF post-randomisation. Individuals with no record of AF during this time frame who died, were withdrawn, or lost to follow-up from the primary care records before 2.5 years from randomisation will be counted as having no record of AF.

## Secondary outcomes

The chi-square test will be used to compare the proportion of participants with AF recorded within a period of 2.5 years after randomisation in those randomised to screening versus those randomised to usual care, separately by age subgroups of <80 and ≥80 years and, separately, by sex subgroups at randomisation, with a test for heterogeneity of the results between the two subgroups.

As the characteristics of the distributions of mean time spent with a known AF diagnosis cannot be predicted beforehand, choice of a parametric statistical hypothesis test a priori could be inappropriate. Therefore, a permutation test^4^ will be used to compare the mean duration of time with AF diagnosed at 5 years after randomisation in all those randomised to screening compared to all those randomised to usual care (in the overall cohort and, separately, by age and by sex subgroups, as above). Duration of time with AF diagnosed will be defined as days from date of the first post-randomisation AF record in the primary care data to 5 years after randomisation (or date of death or withdrawal if this is earlier). Those with no diagnosis of AF by the end of the study will contribute zero time with AF. Details on a procedure to handle participants who withdrew or were lost to follow-up with no record of AF before 5 years will be specified at a later stage (but before any unblinded data analyses are undertaken). The empirical p value for the difference will be calculated as (1+s)/(n+1) where s = the number of simulations where the difference in mean time with a known AF diagnosis is at least the observed difference and n=total number of simulations.

## Sensitivity analyses

Sensitivity analyses of the primary and secondary outcomes will be performed using AF diagnoses captured in either primary care records or secondary care records (i.e. from Hospital Episode Statistics datasets) – i.e. an AF diagnosis will be counted regardless of where it was recorded in primary care or secondary care records.

## Exploratory outcomes

### Time to AF detection within a period of 2.5 and 5 years after randomisation

Cumulative incidence curves for AF detection up to 2.5 years and 5 years after randomisation will also be presented, and the difference between randomised arms will be assessed as an exploratory outcome. Time to AF detection will be defined as the time from randomisation to first post randomisation primary care record of AF, censoring at the earliest of date of death, withdrawal, loss to follow-up in the primary care records, or day 913 (2.5-year analysis) / day 1826 (5-year analysis). Statistical hypothesis testing will be performed using the log-rank test. The median time to first AF record after randomisation in primary records (i.e. from randomisation to first post-randomisation record) will also be presented for each arm for people who have a diagnosis of AF.

### Time spent with a known diagnosis of AF within a period of 2.5 years after randomisation

The outcome of time spent with a known diagnosis of AF within a period of 2.5 years after randomisation (i.e. replicating the secondary outcome of time spent with a known diagnosis of AF within a period of 5 years after randomisation, but within a shorter period) will be assessed as an exploratory outcome alongside analyses of the primary outcome (proportion of participants with a post-randomisation record of AF at 2.5 years after randomisation). The hypothesis testing framework and procedures for this exploratory outcome will follow those outlined for the secondary outcome of time spent with a known diagnosis of AF within a period of 5 years after randomisation (as specified in section 6.2).

### Rates of oral anticoagulation use

To explore the impact of AF screening on post-randomisation oral anticoagulation use (including direct oral anticoagulants and vitamin K antagonists, but excluding rivaroxaban 2.5mg twice daily^[[1]](#footnote-2)^), the following exploratory outcomes will be assessed:

1. Total number of participants with a post-randomisation record (presented using counts and proportions in each randomised arm, and tested using a chi-square test). Median time to first post-randomisation record (in calendar months) will also be presented for people with oral anticoagulation recorded, separately for each randomised arm.
2. Total quantitative post-randomisation exposure, defined as total number of distinct calendar months with evidence of likely exposure to oral anticoagulation, as further described in a separate document (presented using means in each randomised arm, and tested using a permutation test).

All outcomes will be calculated in both study arms, up to 30 and 60 months after randomisation (or month of death or withdrawal if earlier). These assessments will be performed regardless of whether AF was recorded during the follow-up period (as any impact of screening on clinical outcomes would depend on an increase in overall anticoagulation exposure, not just anticoagulation for AF).

Cumulative incidence curves for months to first post-randomisation oral anticoagulant record up to 30 and 60 months after randomisation will also be presented. These analyses will also be censored at the month of death or withdrawal. Statistical hypothesis testing will be performed using the log-rank test.

### Long-term assessments of the clinical impact of screening

Although the trial is not powered to show a statistically significant difference in clinical outcomes between study arms, numbers and proportions of the following events in each arm will be reported in future analyses focused on long-term impacts of screening (with no hypothesis testing performed):

- Hospitalisations (total and for cardiovascular causes)
- Ischaemic stroke
- Major bleeding
- All-cause and cardiovascular death

Details on the nature of such analyses and associated outcome identification will be specified at a later stage.

## Non-randomised comparisons

### Rates of oral anticoagulation between screen-detected and clinically-detected AF (non-randomised assessment)

In order to explore the potential impact of AF detection modality (screen vs clinically-detected) on clinical management, an exploratory, retrospective, non-randomised comparison of rates of post-randomisation oral anticoagulation use will be performed among the following non-randomised groups:

1. AF detected on patch + AF recorded in post-randomisation primary care records
2. AF recorded in post-randomisation primary care records only (amongst those randomised to either treatment arm)
3. AF detected on patch + no post-randomisation primary care record of AF

Anticoagulation use will be defined as:

- For participants with AF detected in primary care records (groups 1 and 2): presence of an anticoagulation record after the month of the first post-randomisation AF primary care record;
- For participants with AF detected on patch but not on primary care records (group 3): presence of an anticoagulation record after the month of reporting of an AF diagnosis to the GP.

For participants with AF detected on patch and AF recorded in post-randomisation primary care records (group 1), the time interval between AF diagnosis reporting to the GP and date of first post-randomisation AF record in primary care data will also be reported. The date of AF diagnosis reporting to the GP will be considered as the date on which the cardiac monitoring report was emailed directly to the GP by the study team, not the (later) date on which the automated results letter was mailed to both the participant and their GP (as described in Section 3.5).

## Non-pre-specified exploratory analyses

Additional analyses not specified in this statistical analysis plan may be performed if deemed to be of scientific interest. These will be clearly identified as exploratory and non-pre-specified, and due allowance will be made in their interpretation.

## Health economics

In addition to investigating the impact of screening on AF detection rates, the results from AMALFI will also inform a broader cost-effectiveness analysis aimed at capturing differences in healthcare resources (e.g. GP visits, medications, hospital visits), as well as differences in health-related quality of life (based on the standard EQ-5D-5L questionnaire), AF cases, and AF-related events in combination with data from external cohorts. Details of these analyses will be described in a separate analysis plan.

## Power calculations

### Power calculations for the primary outcome

The sample size for this study was initially specified as 2,500 but was later increased to 5,000. The underlying assumptions are as follows:

***AF detection via usual care:*** A 2015 UK study on opportunistic AF screening during flu vaccinations suggests the yearly rate of asymptomatic AF detection through usual care is ~0.7% among those who receive a pulse check (with approximately half those who had a flu vaccination receiving a pulse check).^5^ Thus, if considering the entire population aged 65 years or more (of whom only some will have pulse checks), the rate of newly-detected AF is expected to be half, at ~0.3-0.4%/year. As AMALFI participants will generally have a higher cardiovascular risk than the general population, the yearly AF detection rate was estimated to be ~0.7%/year. Over a 2.5 year period, the new AF detection rate via usual care is expected to be 1.75% (0.7% x 2.5 years), based on a time dependent Poisson process (a+bt) with a parameter b slightly increasing with time.

***AF detection via screening:*** In STROKESTOP, screening with twice-daily, 30 second ECG over 14 days in 75 year-olds detected new AF in 3.0%.^6^ It is expected that continuous monitoring with the Zio XT during a similar period of time in AMALFI will result in higher AF detection rates. However, patch screening will not detect all AF cases in the active arm, due to non-compliance with the patch, false negatives, or AF episodes not occurring at the time of screening; thus, additional AF cases will continue to be detected through usual care in this arm. Taking into consideration a moderate level of non-adherence and false-negatives (i.e. AF missed during the monitoring period by chance), it was estimated that screening would lead to newly-detected AF in about 3.75% of individuals, with additional new AF cases found via usual care throughout the duration of study. Over a 2.5-year period, it is estimated that the total new AF detection rate in the active arm will be 4.4%. This estimate takes the same Poisson process used in the usual care arm (with expected annual AF incidence 0.7%) and assumes that 70% of AFs that would have been detected in year 1 were found by patch, 60% year 2, and 50% year 3.

***Sample size calculations:*** Based on these assumptions, an initial sample size of 2,500 individuals (1,250 in each arm) was set to provide >90% power to detect the expected difference in proportions of newly-detected AF between arms at 2.5 years (1.75% versus 4.4%; ratio: approximately 2.5) at two-sided p (2p) <0.05.

***Modifications to the sample size:*** AMALFI completed recruitment of the initial sample size of 2,500 in mid-2021, without exhausting the available pool of interested GP practices or the funding available for recruitment activities. Based on an interim analysis of baseline characteristics and Zio XT AF detection rates in 1,250 randomised participants (but blinded to total AF detection rates in both arms), the investigators decided to extend the target study size to 5,000. This extension allowed:

1. Improved precision to detect the estimated 2.5-fold difference in AF proportions between the two arms at 2.5 years (>90% power at 2p<0.01 vs 0.05);

2. Preserved power if the difference between arms were smaller than expected (i.e. >90% power at 2p<0.01 for a 2-fold difference) – e.g. due to high AF detection rates through usual care in the control arm;

3. Good power to analyse the primary outcome by subgroups for age (<80 and ≥80 years) and, separately, sex at randomisation, with ~90% power at 2p<0.05 in both assessments;

4. Improved power to detect differences in health-related quality of life (health economics analyses).

## Adjustment for baseline characteristics

No adjustment of the primary or secondary outcomes based on baseline characteristics is planned a priori. Adjustments may be performed if significant imbalances between arms (for the overall cohort or any of the pre-specified subgroup analyses) are later found. However, it is expected that the minimisation randomisation algorithm employed (based on age, sex, and self-reported residual CHA_2_DS_2_VASc score) will prevent or at least reduce the possibility of such imbalances.

## Significance levels and adjustment of p-values for multiplicity

The statistical significance level will be set as a two-sided alpha<0.05. Formal statistical adjustment for multiple hypothesis testing will not be made for the primary, secondary and exploratory outcomes (including subgroup analyses). However, due allowance for multiple testing will be made in the interpretation of the secondary and exploratory results: the larger the number of events on which a comparison is based, and the more extreme the P-value after any allowance has been made for the nature of the particular comparison (i.e. primary or secondary; pre-specified or exploratory), the more reliable the comparison and, hence, the more definite any finding will be considered. 95% confidence intervals will be presented for comparisons of the primary and secondary outcomes.

# Statistical software

SAS version 9.4 and/or R version 4.2.2 in RStudio version 2024.12.1. Build 563 (or later) for Windows will be used for all statistical analyses and plotting.

# References

1. Hindricks G, Potpara T, Dagres N*, et al.* 2020 ESC Guidelines for the diagnosis and management of atrial fibrillation developed in collaboration with the European Association for Cardio-Thoracic Surgery (EACTS). *European Heart Journal* 2021;**42**:373-498. doi: 10.1093/eurheartj/ehaa612

2. Pocock SJ, Simon R. Sequential treatment assignment with balancing for prognostic factors in the controlled clinical trial. *Biometrics* 1975;**31**:103-115. doi:

3. Schulz KF, Altman DG, Moher D. CONSORT 2010 Statement: updated guidelines for reporting parallel group randomised trials. *BMC Medicine* 2010;**8**. doi: 10.1186/1741-7015-8-18

4. Lachin JM. Statistical properties of randomization in clinical trials. *Controlled Clinical Trials* 1988;**9**:289-311. doi: 10.1016/0197-2456(88)90045-1

5. Arathoon N. Opportunistic Screening for Atrial Fibrillation for over 65 Year Olds during Flu Clinics and Anticoagulation for the Prevention of Stroke in Primary Care. . <https://aftoolkit.co.uk/wp-content/uploads/2020/04/dorset-ccg.pdf>

6. Svennberg E, Friberg L, Frykman V*, et al.* Clinical outcomes in systematic screening for atrial fibrillation (STROKESTOP): a multicentre, parallel group, unmasked, randomised controlled trial. *The Lancet* 2021;**398**:1498-1506. doi: 10.1016/s0140-6736(21)01637-8

# Appendices

## Appendix I: CHA_2_DS_2_-VASc Scoring system

| Item | Condition | Points |
| --- | --- | --- |
| C | Congestive heart failure (or Left ventricular systolic dysfunction) | 1 |
| H | Hypertension: blood pressure consistently above 140/90 mmHg (or treated hypertension on medication) | 1 |
| A_2_ | Age ≥75 years | 2 |
| D | Diabetes Mellitus | 1 |
| S_2_ | Prior Stroke or TIA or thromboembolism | 2 |
| V | Vascular disease (e.g. peripheral artery disease, myocardial infarction, aortic plaque) | 1 |
| A | Age 65–74 years | 1 |
| Sc | Sex category (i.e. female sex) | 1 |

1. Rivaroxaban 2.5mg BD is the dose used for cardiovascular risk reduction alongside antiplatelet therapy in people with established cardiovascular disease, and not for clinical anticoagulation purposes, hence excluded here [↑](#footnote-ref-2)
