# Supplementary material for: Active Monitoring for AtriaL FIbrillation (AMALFI): Rationale, protocol, and pilot for a pragmatic, randomized, controlled trial of remote screening for asymptomatic atrial fibrillation
Source: Am Heart J. Author manuscript; Available in PMC 2026 Mar 10. (PMC7618845; doi:10.1016/j.ahj.2025.07.004)
Supplement: Supplement [file EMS212696-supplement-Supplement.docx]

# Supplementary materials

**Contents**

S1. Statistical assumptions underlying the expected cumulative AF incidence rate curve 2

S2. Sample size calculations 2

Supplementary Tables

Supplementary Table S1: List of GP Practices taking part in AMALFI 3

Supplementary Table S2. Initial projections for proportions of new atrial fibrillation 4
(AF) cases found in the active and control groups at different time points

Supplementary Table S3. Sample size calculations based on the initial trial 5 assumptions

Supplementary Table S4. Updated power and sample size calculations under 6 different assumptions regarding AF rates detected by patch and detection
ratio between active and control groups

Supplementary Table S5. Power and sample size calculations for subgroup 7 analyses by age and sex

Supplementary Appendix S1. Sample Zio Report [separate file]

Supplementary Appendix S2. Study Protocol [separate file]

Supplementary Appendix S3. Definition and Derivation of Baseline Characteristics and Outcomes [separate file]

Supplementary Appendix S4. Statistical Analysis Plan [separate file]

## S1. Statistical assumptions underlying the expected cumulative AF incidence rate curve

The probability of being diagnosed with AF is a time dependent Poisson process with a parameter slightly increasing with time, a+bt. Additionally, we assumed that if someone would have had AF diagnosed at time t, there is a decreasing probability e+ft that it would be pre-empted by patch related early detection of AF. We then chose a and b so that the AF diagnosis rates during years 1 and 2 were both 0.7% per annum, the anticipated detection rate with usual care. e and f were chosen to allow for non-compliance and to give decreasing probabilities over time of AFs that would have been diagnosed during a time period being pre-emptively detected by patch (70% in the first year, 60% in the second year, 50% in the third, 40% in the fourth and 30% in the fifth year). An additional probability of 2% was assumed for patch related early detection of AF when no AF would have been detected within 5 years. It is assumed that patch related early detection of AF occurs evenly over the first 6 months from randomization. Supplementary Table S2 gives the estimated cumulative % of participants being diagnosed with AF at the end of each year, based on this model and the assumptions described in Section 8 of the protocol.

## S2. Sample size calculations

Supplementary Table S3 gives a set of power calculations based on the expected proportions diagnosed with AF at half yearly points up to 5 years. It is assumed that participants are randomized 1:1 to give to equal numbers in the control and active arms. The 2.5 year row is highlighted as being a suitable mid-study comparison time point for a proposed screening interval of 5 years.

Supplementary Table S4 provides a summary of power calculations for different samples sizes made possible by an extension in recruitment of up to 5000 participants for the whole cohort and under different assumptions regarding AF detection ratios. Supplementary Table S5 provides the same calculations for different age and sex subgroups.

## Supplementary Table S1: List of GP Practices taking part in AMALFI

| **GP practice name** | **GP practice code (ODS)** |
| --- | --- |
| 19 Beaumont Street Surgery | K84016 |
| 3W Health - Norden House Surgery | K82070 |
| 3W Health - Whitchurch Surgery | K82070 |
| 3W Health - Wing Surgery | K82070 |
| Banbury Cross Health Centre | K84028 |
| Bicester Health Centre | K84052 |
| Broadshires Health Centre | K84075 |
| Courtside Surgery | L81024 |
| Eynsham Medical Group | K84006 |
| Gosford Hill Medical Centre | K84045 |
| Iver Medical Centre | K82006 |
| Montgomery House Surgery | K84038 |
| New Wokingham Road Surgery | K81080 |
| Portishead Medical Group | L81004 |
| River Brook Medical Centre | M85156 |
| St Bartholomew's Medical Centre | K84013 |
| Summertown Group Practice | K84011 |
| The Boat House Surgery | K81012 |
| The Hall Practice | K82008 |
| The Swan Practice | K82007 |
| Unity Health | K82047 |
| Watlington & Chalgrove Surgeries | K84008 |
| West Heath Surgery | M85007 |
| White Horse Medical Practice | K84051 |
| Windrush Medical Practice | K84017 |
| Wokingham Medical Centre | K81022 |
| Woodley Centre Surgery | K81051 |

## Supplementary Table S2. Initial projections for proportions of new atrial fibrillation (AF) cases found in the active and control groups at different time points

|  | **Placebo arm** | | **Active arm** | | | | |
| --- | --- | --- | --- | --- | --- | --- | --- |
| **Year end** | **% of patients diagnosed with AF within the year** | **Cumulative % of patients diagnosed with AF by end of year** | **% of patients who would have been found to have AF in year i, but found up front by patch** | **% of patients diagnosed by patch** | **Additional % of patients found to have AF within the year, but not with patch** | **% of patients diagnosed with AF within the year (including 2% extra found by patch, who wouldn’t have been diagnosed with AF during years 1-5)** | **Cumulative % of patients diagnosed with AF by end of year** |
| 1 | 0.70% | 0.70% | 70% | 0.49% | 0.21% | 3.96% | 3.96% |
| 2 | 0.70% | 1.40% | 60% | 0.42% | 0.28% | 0.28% | 4.24% |
| 3 | 0.70% | 2.10% | 50% | 0.35% | 0.35% | 0.35% | 4.59% |
| 4 | 0.70% | 2.80% | 40% | 0.28% | 0.42% | 0.42% | 5.01% |
| 5 | 0.70% | 3.50% | 30% | 0.21% | 0.49% | 0.49% | 5.50% |

## Supplementary Table S3. Sample size calculations based on the initial trial assumptions

| **Comparison time point** | **Expected proportion with AF at time point** | | **Alpha** | **Power** | **Total sample size (continuity corrected)** |
| --- | --- | --- | --- | --- | --- |
|  | **Control arm** | **Active arm** |  |  |  |
| 1 | 0.70% | 3.96% | 0.05 | 90% | 1019 |
| 1.5 | 1.05% | 4.10% | 0.05 | 90% | 1261 |
| 2 | 1.40% | 4.24% | 0.05 | 90% | 1565 |
| 2.5 | 1.75% | 4.41% | 0.05 | 90% | 1914 |
| 3 | 2.10% | 4.59% | 0.05 | 90% | 2348 |
| 3.5 | 2.45% | 4.80% | 0.05 | 90% | 2825 |
| 4 | 2.80% | 5.01% | 0.05 | 90% | 3406 |
| 4.5 | 3.15% | 5.25% | 0.05 | 90% | 4004 |
| 5 | 3.50% | 5.50% | 0.05 | 90% | 4711 |

## Supplementary Table S4. Updated power and sample size calculations under different assumptions regarding AF rates detected by patch and detection ratio between active and control groups

| **Assumptions** | | | | | | **Total number of participants needed**  **(with 90% power)** | | **Power for a total number of n participants at different levels of statistical significance** | | | | |
| --- | --- | --- | --- | --- | --- | --- | --- | --- | --- | --- | --- | --- |
| **Group** | **Percent of population** | **AF rate detected through patch in active arm** | **Total AF rate in active arm at 2.5 years (patch rate x 1.177)** | **Ratio of AF rates in active vs control arms** | **Implied AF rate in controls (based on detection ratio)** | **At 2p<0.05** | **At 2p<0.01** | **n=2500, 2p<0.05** | **n=4000, 2p<0.05** | **n=4000, 2p<0.01** | **n=5000, 2p<0.05** | **n=5000, 2p<0.01** |
| Whole trial (original assumptions) | 100% | 3.75% | 4.41% | 2.52 | 1.75% | 1914 | 2649 | 96% | 100% | 99% | 100% | 99% |
| Whole trial | 100% | 3.75% | 4.41% | **2.5** | 1.77% | 1941 | 2687 | 96% | 100% | 99% | 100% | 100% |
| Whole trial | 100% | **4.09%*** | 4.82% | **2.5** | 1.93% | 1773 | 2454 | 97% | 100% | 99% | 100% | 100% |
| Whole trial | 100% | 3.75% | 4.41% | **2** | 2.21% | 2939 | 4088 | 84% | 97% | 89% | 99% | 96% |
| Whole trial | 100% | **4.09%*** | 4.82% | **2** | 2.41% | 2684 | 3732 | 88% | 98% | 92% | 99% | 97% |

* interim AF detection rate by patch (blinded to total AF rates in both groups)

## Supplementary Table S5. Power and sample size calculations for subgroup analyses by age and sex

| **Assumptions** | | | | | | **Number of participants needed in subgroup**  **(with 90% power)** | | **Power for a total number of n participants at different levels of statistical significance** | | | |
| --- | --- | --- | --- | --- | --- | --- | --- | --- | --- | --- | --- |
|  |  |  |  |  |  |  |  | **n=4000** | | **N=5000** | |
| **Group** | **Percent of population** | **AF rate detected through patch in active arm*** | **Total AF rate in active arm at 2.5 years (patch rate x 1.177)** | **Ratio of AF rates in active vs control arms** | **Implied AF rate in controls (based on detection ratio)** | **At 2p<0.05** | **At 2p<0.01** | **At 2p<0.05** | **At 2p<0.01** | **At 2p<0.05** | **At 2p<0.01** |
| **Age <80** | 64% | 2.78% | 3.27% | **2.5** | 1.31% | 2642 | 3658 | 89% | 73% | 95% | 85% |
| **Age ≥80** | 36% | 6.28% | 7.39% | **2.5** | 2.95% | 1135 | 1571 | 96% | 87% | 99% | 95% |
| **Age <75** | 25% | 2.52% | 2.96% | **2.5** | 1.18% | 2924 | 4048 | 41% | 20% | 52% | 34% |
| **Age ≥75** | 75% | 4.62% | 5.44% | **2.5** | 2.18% | 1563 | 2164 | 100% | 98% | 100% | 100% |
| **Males** | 54% | 4.94% | 5.82% | **2.5** | 2.33% | 1458 | 2018 | 98% | 92% | 99% | 98% |
| **Female** | 46% | 3.09% | 3.64% | **2.5** | 1.46% | 2367 | 3276 | 80% | 60% | 89% | 75% |

* interim AF detection rate by patch (blinded to total AF rates in both groups)
